# Cobalt-catalyzed direct carbonylative 3-acylation of (N–H)Indoles with alkyl halides

**DOI:** 10.1039/d5sc05810d

**Published:** 2025-09-05

**Authors:** Chao Xu, Zhi-Peng Bao, Sufang Shao, Xiao-Feng Wu

**Affiliations:** a Dalian National Laboratory for Clean Energy, Dalian Institute of Chemical Physics, Chinese Academy of Sciences Dalian China Xiao-Feng.Wu@catalysis.de xwu2020@dicp.ac.cn; b University of Chinese Academy of Sciences Beijing China; c Leibniz-Institut für Katalyse e.V. Rostock Germany

## Abstract

A cobalt-catalyzed approach to intermolecular carbonylative 3-acylation of heterocycles *via* C–H functionalization is described. This transformation enables the direct C–H acylation of indoles and pyrroles with alkyl halides. Notably, this procedure is also compatible with indoles containing unprotected NH groups. Overall, this methodology represents an atom-economical and general strategy for synthesizing alkyl-(hetero)aryl ketones.

## Abstract 2

A cobalt-catalyzed approach to intermolecular carbonylative 3-acylation of heterocycles *via* C–H functionalization is described.

## Graphical abstract

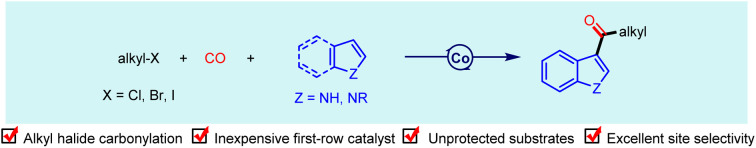

## Introduction

**Scheme 1:**
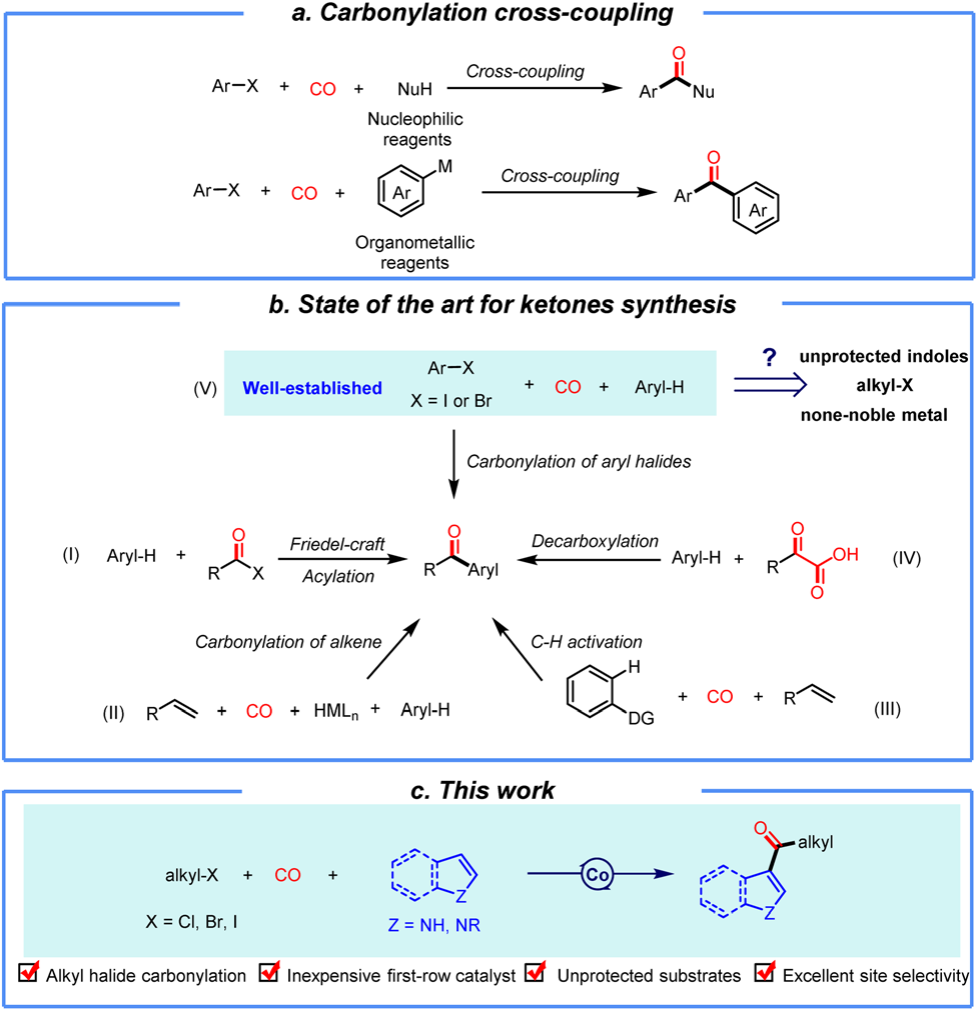
(a) Ketone formation *via* traditional carbonylative methods. (b) C–H functionalization of arenes. (c) This work.

Metal-catalyzed carbonylation reactions represent an important class of transformations that efficiently afford carbonyl-containing compounds by using CO as a C1 building block.^1^ Among them, carbonylative cross-coupling can form various products such as amides, esters, and thioesters by using different stable and readily available nucleophiles (Scheme 1a).^2^ However, in contrast to the well-established construction of C–X bonds (*e.g.*, C–N, C–O, and C–S),^2^ the synthesis of ketones with carbon nucleophiles *via* C–C bond formation remains relatively underexplored. This situation is mainly due to the decreased nucleophilicity of the reaction partner which leads to different reaction pathways. Among diverse carbonyl containing compounds, (hetero)aromatic ketones serve as crucial motifs in industrial chemistry, drug discovery, and the development of advanced materials and polymers.^3^ Since the pioneering work of Heck and co-workers in the 1970s,^4^ efforts towards carbonylative cross-coupling for ketone synthesis have primarily focused on the use of organometallic reagents as the *C*-nucleophiles, such as organoboron, organozinc, and organosilicon compounds.^5^ Unfortunately, cross-coupling reactions involving organometallic reagents present two problems: on the one hand, pre-preparation of organometallic reagents is required, and most organometallic reagents have poor stability and are difficult to store (sensitive to oxygen and moisture); on the other hand, organometallic reagents require stoichiometric amounts of metals, which results in additional waste.

In view of these challenges, it is reasonable to have an idea that synthesis of ketones can be achieved by directly activating C–H bonds and using them as the coupling partners. In this reaction mode, the Friedel–Crafts acylation reaction is one of the most well-known strategies for constructing carbonyl-containing (hetero)aryl ketones (Scheme 1, b-I).^6^ However, electrophilic acylation reagents necessitate prior preparation and frequently rely on activating agents (*e.g.*, SOCl_2_, PCl_3_, and strong Lewis acids), which are accompanied by substantial chemical waste generation. Additionally, the high reactivity of these electrophilic reagents results in poor functional group tolerance, thereby restricting the widespread application of Friedel–Crafts acylation reactions. To address these limitations, strategies for *in situ* generation of active acyl species have been developed. For example, in Reppe-type carbonylation reactions, aryl ketones are constructed *via* a metal hydride-mediated pathway involving alkenes and CO (Scheme 1, b-II).^7^ This process can be achieved with only a catalytic amount of protic acid; however, the regioselectivity between linear and branched derivatives increases the reaction complexity. In C–H bond activation strategies, either pre-installed directing groups are required or reactions are confined to intramolecular carbonylation, significantly limited the substrate generality (Scheme 1, b-III).^8^ Moreover, decarboxylative acylation relies on keto acid substrates, which increase substrate accessibility challenges (Scheme 1, b-IV).^9^ One frequently investigated approach is metal-catalyzed cross-coupling carbonylation between aryl halides and (hetero)aromatic rings (Scheme 1, b-V).^10^ Nevertheless, carbonylative coupling of alkyl halides with (hetero)aromatic rings remains unreported, and studies on indole substrates are restricted to N-substituted derivatives. Furthermore, such transformations are still limited to noble palladium catalysis.

To address the discussed challenges in carbonylation reactions, we developed an abundant cobalt-catalyzed carbonylative coupling reaction between alkyl halides and (hetero)arenes. This innovation has two key features: first, it achieves the first direct carbonylative coupling of alkyl halides with (hetero)arenes, overcoming the traditional reliance on aryl halides and extending the substrate scope to N–H indoles which significantly enhance the reaction's generality. Second, it replaces precious palladium with low-cost cobalt, drastically reducing costs while aligning with the sustainability imperatives of green chemistry. By eliminating the need for pre-prepared reactive reagents, this reaction enables efficient construction of key (hetero)aryl alkyl ketone structures, providing a streamlined strategy for pharmaceutical and material synthesis. This advancement represents an improvement in driving carbonylation reactions toward greater environmental benignity and versatility.

## Results and discussion

To establish this reaction, chloroacetonitrile (1a) and *N*-methyl indole (2a) were selected as model substrates to explore the optimal conditions under CO (40 bar) pressure. The carbonylated product 3a was produced in 31% yield when the reaction was carried out at 60 °C in acetonitrile with 10 mol% of CoCl_2_·6H_2_O as the catalyst, 5 mol% of 5-(methoxycarbonyl) picolinic acid L2 as the ligand, 5 mol% of Mn as the reductant, and Na_2_CO_3_ as the base (Table 1, entry 1). Then a series of pyridine carboxylic acid and pyridine amide ligands were evaluated, among which L2 exhibited the best performance (Table 1, entry 2). Increasing the temperature was beneficial for improving the conversion rate of raw materials; when the reaction temperature was raised to 80 °C, the yield of the target product 3a increased to 62% (Table 1, entry 3). Alternating solvents had an adverse effect on the reaction; most of them inhibited the reaction. The yields of 3a in tetrahydrofuran and dimethoxyethane were 14% and 25%, respectively (Table 1, entries 4–5). Various cobalt salts were evaluated to regulate the yields, Co(acac)_2_ provided the highest yield of 3a of 73% (Table 1, entry 6). Metallic cobalt and manganese powder reducing agents were proven necessary (Table 1, entries 7–8). Increasing the equivalent of 1a further improved the conversion rate. When 4.0 equivalents of 1a were used, the reaction yield of 3a reached 95%, and product 3a was obtained with a 91% isolated yield (Table 1, entry 9). When the pressure of CO was reduced to 30 bar, a similar yield was obtained (Table 1, entry 10). However, the yield drops significantly if we further decrease the pressure of CO.

**Table 1:** Optimization studies^a^^,^^b^

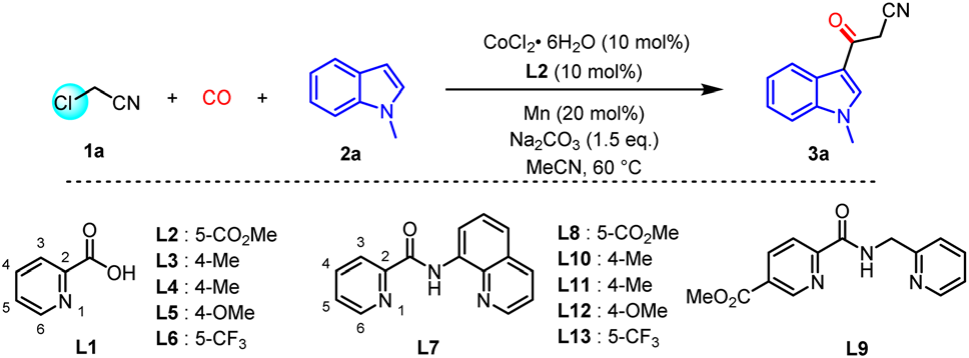		
Entry	Deviation from above	Yield^a^ (%)
1	None	31
2	Other ligands	0–30
3	80 °C	62
4	THF	14
5	DME	25
6	Co(acac)_2_	73
7	W/o [Co]	0
8	W/o Mn	0
9	1a (4.0 eq.)	95 (91^b^)
10	30 bar	95

a Reaction conditions: 1a (0.45 mmol), 2a (0.3 mmol), CO (40 bar), CoCl_2_·6H_2_0 (10 mol%), L2 (10 mol%), Mn (10 mol%), Na_2_CO_3_ (0.45 mmol), MeCN (1.5 mL), 80 °C, 12 h. Yields were determined by GC with dodecane as an internal standard.

b Isolated yield.

**Scheme 2:**
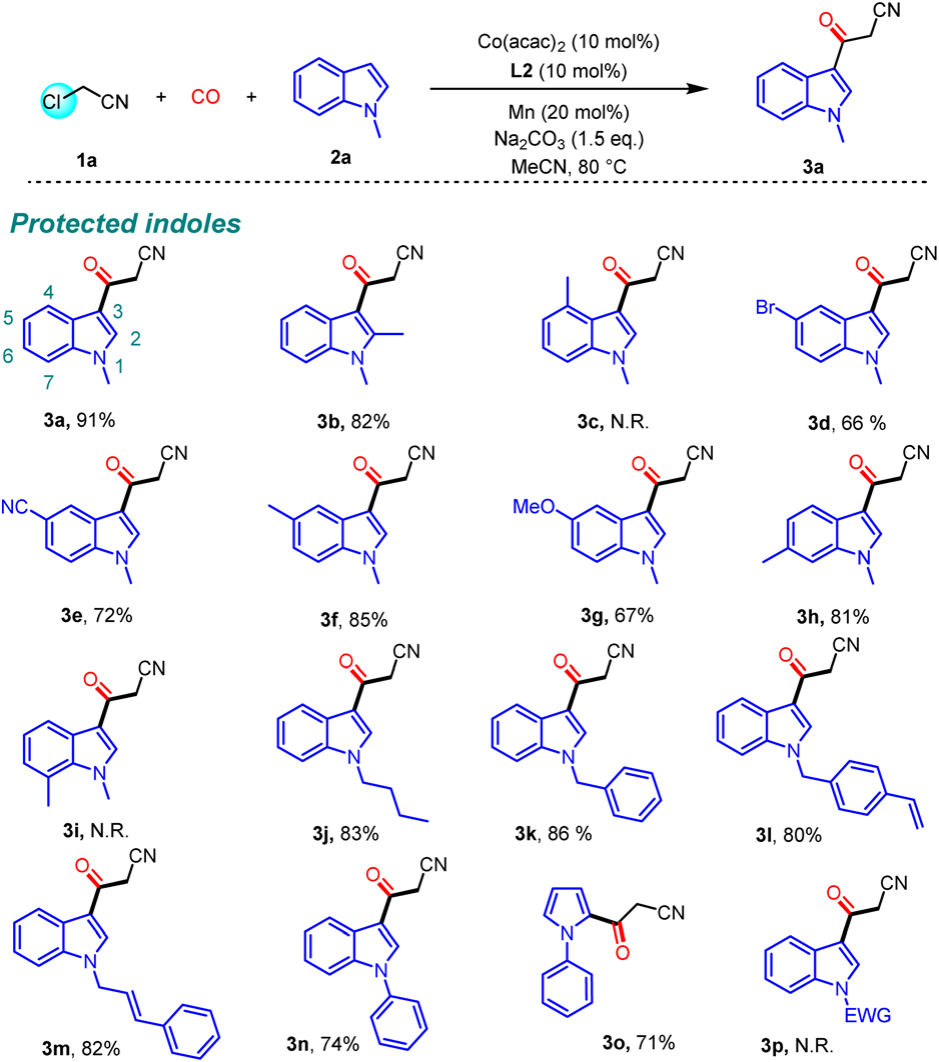
Substrate scope of N-protected indoles.

With the optimized conditions in hand, the generality of this new protocol was investigated (Scheme 2). A series of N-substituted indoles were evaluated and afforded medium to good yields of the desired 3-acylated products. In general, electron-donating groups provided higher yields, which may be due to the higher nucleophilicity of the electron-rich aromatic rings (3a–3i). In addition, substitutions at the 4-position and 7-position of the indole ring completely inhibited the reaction (3c and 3i). Various indoles with electron-donating substituents were investigated, among which alkyl, alkenyl, cinnamyl, and phenyl groups were all well compatible (3j–3n). It is worth mentioning that the product from *N*-phenylpyrrole was also obtained with a yield of 71% (3o) under the same reaction conditions. Unfortunately, indoles substituted with various electron-withdrawing protecting groups failed to yield the target product. On the other hand, the need for N-protection will undoubtedly limit the application of this strategy as deprotection is usually tedious.

**Scheme 3:**
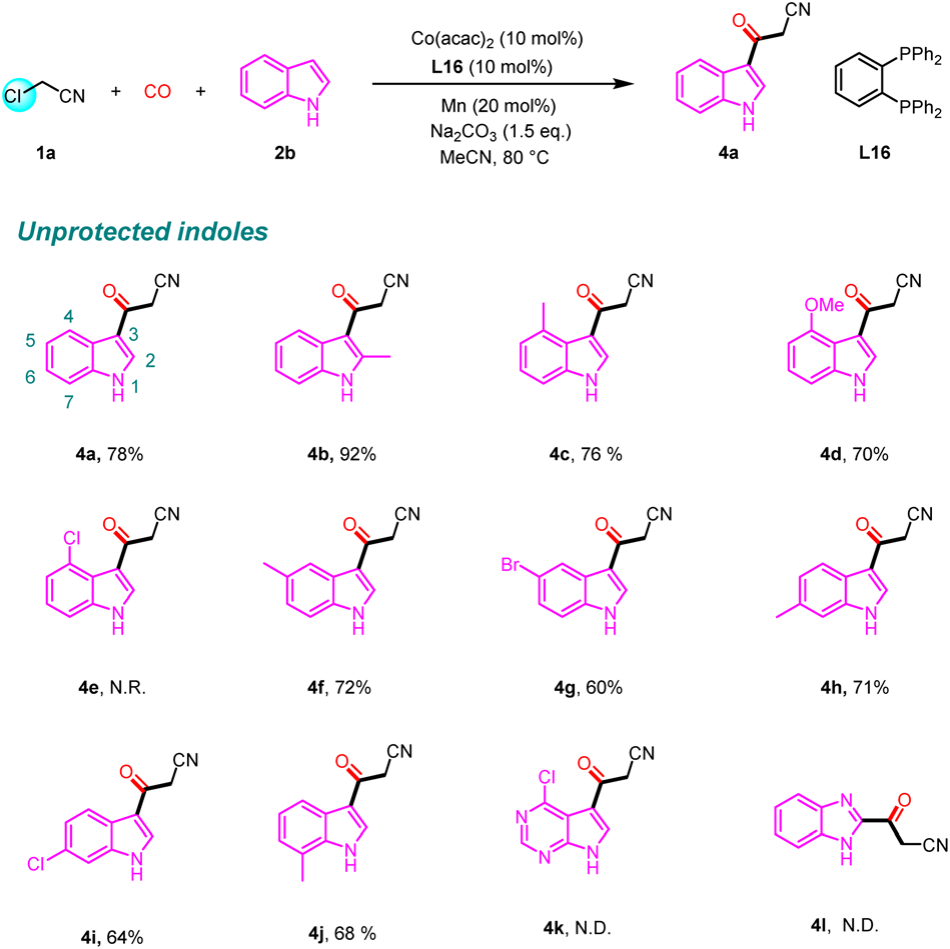
Substrate scope of unprotected indoles.

To expand the scope of this protocol, we become interested in overcoming the mentioned limitation and realize using unprotected indoles as substrates. Fortunately, the target product 4a was successfully obtained when a bulky phosphine ligand (L16) was used with the nucleophilic NH group unprotected (Scheme 3). When the 4-position of indole was substituted, the corresponding product can be obtained in good yield (76%, 4c and 70%, 4d), which was previously restricted (3c). However, when electron-withdrawing chloro-substituted substrates are used, the reaction was inhibited (4e). The reactivity of substrates substituted at the 5-position and 6-position of indole was less affected by electronic properties (4f–4i). When indoles substituted at the 7-position were used, the target product was obtained (68%, 4j), to overcome the limitation of 3i. Regrettably, when other heterocycles were used, the target product was not detected in this protocol (4k–4l).

**Scheme 4:**
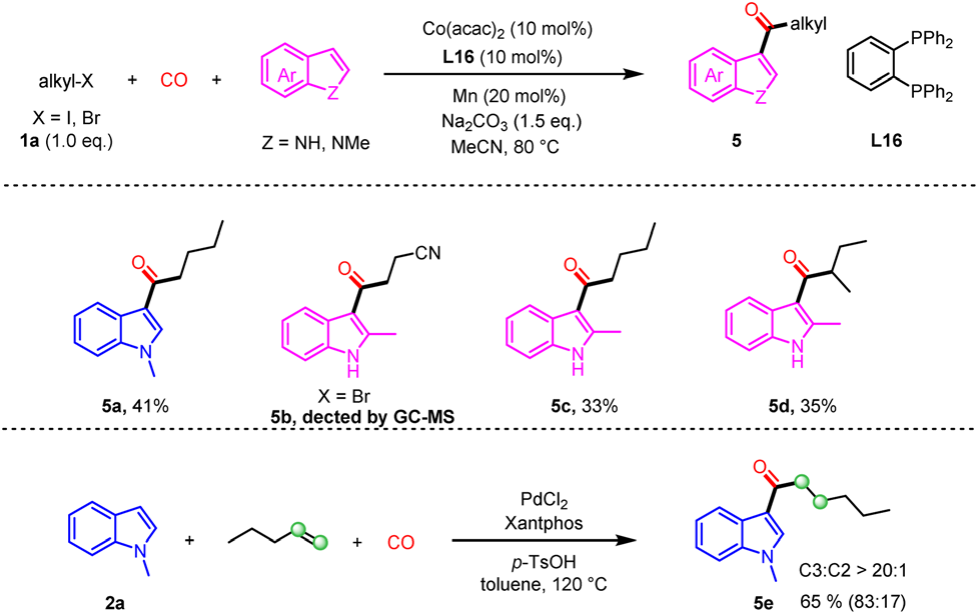
Substrate scope of alkyl iodides.

Subsequently, long-chain alkyl halides were brought into the scope of investigation (Scheme 4). Despite modest reaction yields, the reaction exhibited exceptional selectivity. The combination of CO and alkyl halides demonstrated selectivity profiles consistent with those observed using acyl chlorides. In the palladium-catalyzed carbonylation transformations reported in the literature,^7^ limitations exist in the selectivity between linear and branched products, which poses challenges for the separation and purification of the target compounds. However, the tests with α-bromocarbonyl esters and difluoroalkyl halides lead to no desired product detectable.

**Scheme 5:**
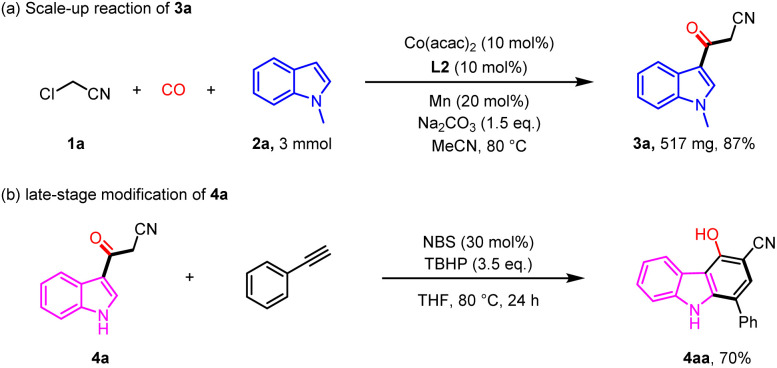
Scale-up reaction and late-stage modification of 4a.

To further demonstrate the synthetic potential of the carbonylation protocol, we carried out a 3 mmol scale experiment and successfully obtained 571 mg of compound 3a with 87% yield (Scheme 5a). Carbazole is widely used in fields such as dyes, lubricants, and pesticides.^11^ Substituted carbazole compound 4aa can be conveniently obtained from 4a in 70% yield (Scheme 5b).^12^

**Scheme 6:**
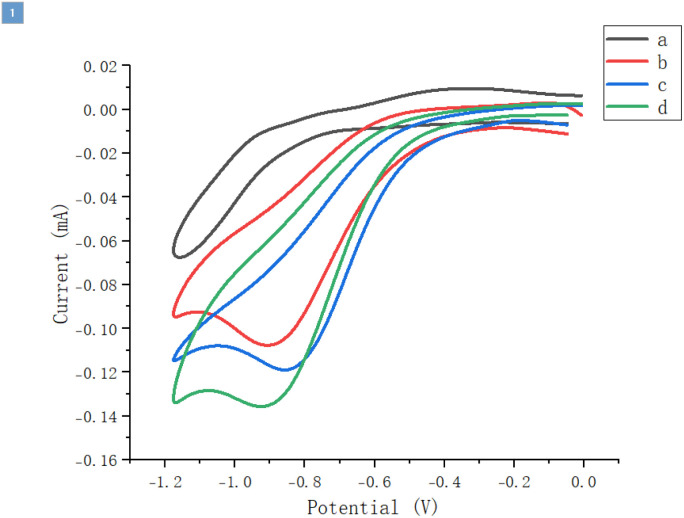
Cyclic voltammograms recorded on a Pt electrode at 100 mV s^−1^ in (a) MeCN containing 0.1 M of ^*n*^Bu_4_NPF_6_, black; (b) solution (a) with 0.2 mM of chloroacetonitrile added, red; (c) solution (a) with 0.05 mM of Co(OAc)_2_·4H_2_O and L2 added, blue; (d) solution (c) with 0.2 mM of chloroacetonitrile added, green.

To gain an understanding of the reaction mechanism, preliminary cyclic voltammetry tests were carried out (Scheme 6).^13^ The reduction peak of chloroacetonitrile was approximately at −0.9 V, and the reduction potential of Co(ii) to Co(i) was around −0.84 V, with the two being relatively well-matched. When chloroacetonitrile was added to the cobalt catalytic system, the catalytic current was significantly enhanced (by comparing curve d with curves b and c), which might be due to the one-electron reduction of chloroacetonitrile by the *in situ* generated Co(i).

**Scheme 7:**
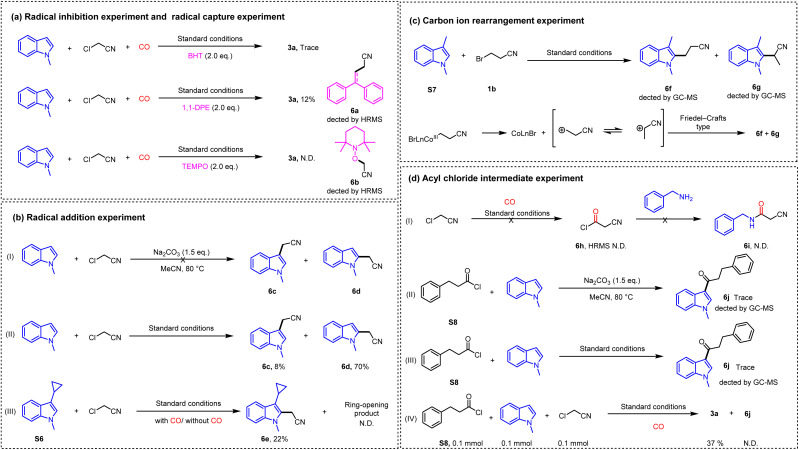
Mechanistic studies.

Subsequent mechanistic studies (Scheme 7) began with radical trapping experiments. Addition of various scavengers to the standard reaction significantly inhibited the product formation, supporting a radical pathway. HRMS analysis confirmed the formation of trapping products 6a and 6b, though in low yields. This was rationalized by competitive trapping: the rapid coordination of acetonitrile radicals to the cobalt catalyst likely outcompeted scavenger addition (Scheme 7, eq. a).

To validate radical addition, we first excluded nucleophilic substitution by demonstrating that no reaction occurred in the absence of a catalyst (Scheme 7b-I). Under catalytic conditions, 6c and 6b were isolated in a ratio of <10 : 1 (Scheme 7b-II),^14^ suggesting an alternative mechanism. Testing substrate S6 revealed that the unopened product 6e was isolated in 22% yield regardless of CO presence, while the ring-opened isomer remained undetected (Scheme 7b-III). These results cast doubt on a radical addition pathway.

The formation of 6c and 6d instead implicated a Friedel–Crafts-type process. Given the propensity of such reactions for carbocation rearrangements, we designed experiments using S6 and 1b as substrates. Detection of rearranged products 6f and 6g (Scheme 7c) provided strong evidence for a carbocation intermediate.

Based on the reports on electrophilic acyl chloride intermediates,^15^ verification experiments targeting such intermediates were conducted (Scheme 7d). In the absence of N-substituted indoles, 6h was not detected under standard reaction conditions; further addition of an equivalent amount of benzylamine also failed to yield the corresponding amide 6i (Scheme 7d-I). When S7 was employed, only trace amounts of 6j could be detected (Scheme 7d-II and d-III). Subsequent competitive reactions with equimolar substrates afforded 3a in 37% yield, while 6j remained undetected (Scheme 7d-VI). Evidently, in this catalytic system, chloroacetonitrile exhibits significantly higher reactivity than acyl chloride, and this protocol does not proceed *via* an acyl chloride intermediate.

**Scheme 8:**
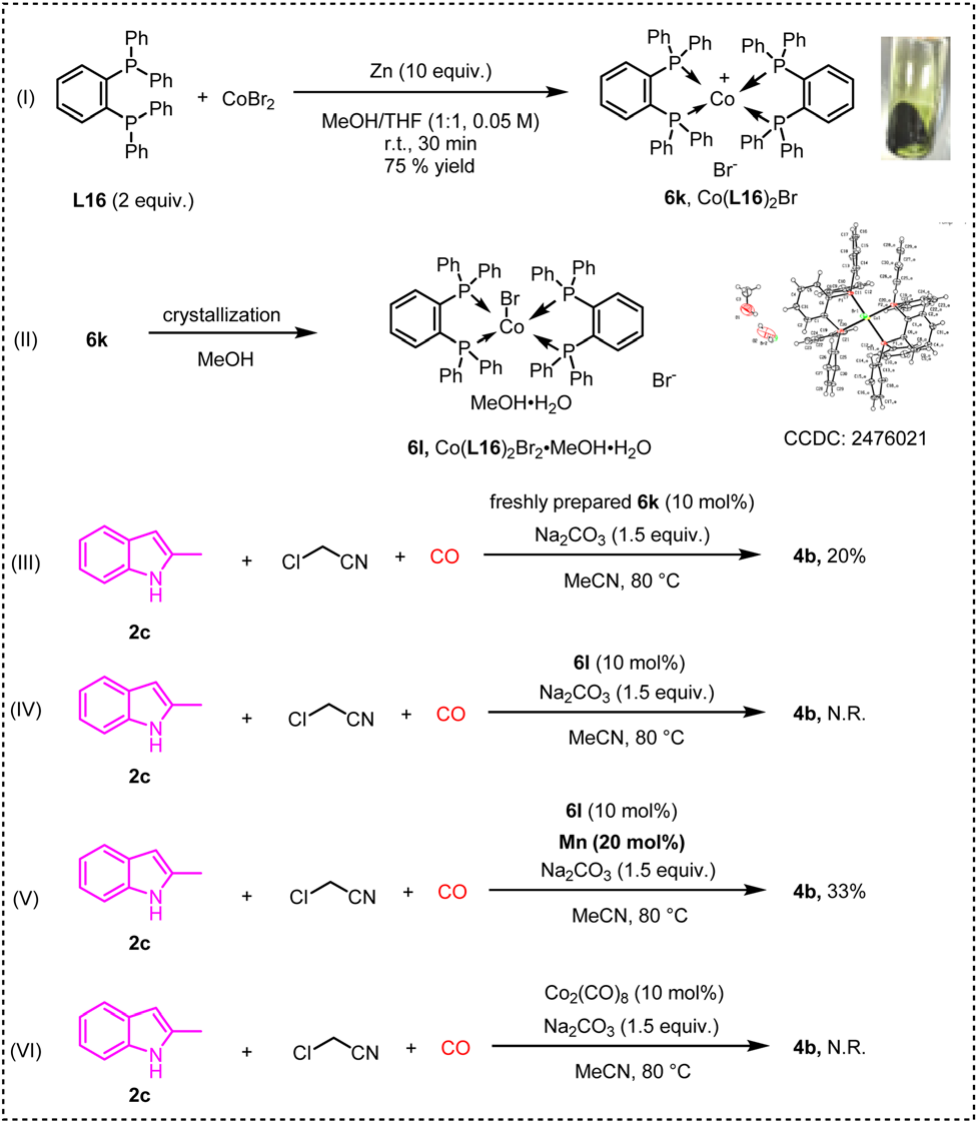
Synthesis of [Co] and its reactivity.

According to the protocol described in the literature, a cobalt complex was synthesized (Scheme 8).^16^ The newly prepared 6l was a dark green solid, consistent with the literature. Unfortunately, during the process of single crystal cultivation, 6l turned from dark green to brownish red. Single crystal diffraction indicates that the brownish red solid was a cobalt(ii) complex, oxidized from cobalt(i) 6l. Next, a set of control experiments were conducted to verify this hypothesis. Using the newly prepared 6k, 4b could be obtained with a 20% yield, demonstrating the catalytic activity of 6k, whereas no target product was observed when 6l was used in the reaction. When a catalytic amount of manganese powder was added, 6l was found to regain its catalytic activity. Meanwhile, the possible zero-valent cobalt pathway was preliminarily ruled out.

**Scheme 9:**
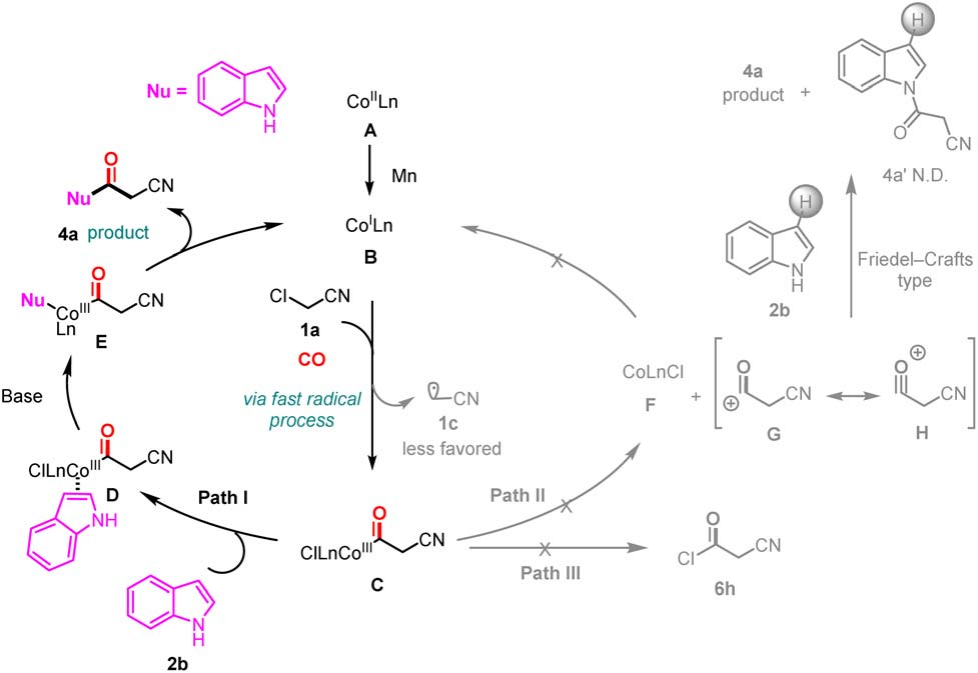
Plausible mechanism.

Based on our results and related literature,^17^ a plausible reaction mechanism is proposed (Scheme 9). First, the cobalt(ii) catalyst was reduced to cobalt(i) by manganese powder. Subsequently, chloroacetonitrile and species B undergo a rapid single-electron transfer, accompanied by migratory insertion of CO to form the acyl metal complex C. Subsequently, 2a coordinated with intermediate C, went through intermediate D, and then generated intermediate E with the assistance of a base. The product 4a was obtained *via* reductive elimination, and the metal cobalt entered the next catalytic cycle. The free acyl cations G or H were considered undesirable because no 4a′ product was detected, thus ruling out the possibility of pathways II and III.

## Conclusions

In conclusion, we have reported a new approach for cobalt-catalyzed carbonylative 3-acylation of indoles. This approach utilizes stable and readily available alkyl halides and avoids the pre-functionalization of heterocycles such as installation of directing groups or protection of active NH groups. It provides an efficient route with high atom economy for the synthesis of functionalized heterocyclic compounds. The excellent selectivity of this method serves as a valuable complement to mild and green Friedel–Crafts acylation reactions.

## Author contributions

C. X. performed all the experiments and prepared the manuscript and SI. Z. P. B. and S. S. prepared some substrates. X. F. W. conceived the project, supervised the research, and revised the manuscript.

## Conflicts of interest

There are no conflicts to declare.

## Supplementary Material

SC-016-D5SC05810D-s001

SC-016-D5SC05810D-s002

## Data Availability

CCDC 2476021 contains the supplementary crystallographic data for this paper.^18^ The data supporting this article have been included as part of the SI. Supplementary information: General comments, general procedure, analytical data, and NMR spectra. See DOI: https://doi.org/10.1039/d5sc05810d.
