# Supplementary material for: Cobalt-catalyzed direct carbonylative 3-acylation of (N–H)Indoles with alkyl halides
Source: Chem Sci. 2025 Sep 5;16(40):18838–43. doi: 10.1039/d5sc05810d (PMC12435503; doi:10.1039/d5sc05810d)

Supplementary Information (SI) for Chemical Science.
This journal is © The Royal Society of Chemistry 2025

Supporting Information

1 General remarks **2**
2 Structures of *N*-Substituted Indoles and Ligands **3**
3 General procedure for the synthesis of starting materials **4**
4. Optimization of reaction conditions **7**
5. General procedure for *N*-protected indoles **10**
6. General procedure for unprotected indoles **11**
7. General procedure for alkyl iodides **11**
8. Scale-up reaction of 3a **12**
9. late-stage modification of 4a **12**
10. Cyclic voltammetry **13**
11. Mechanism experiments **14**
11.1 Radical inhibition experiment and radical capture experiment 14
11.2 Radical addition experiments 16
11.3 Carbon ion rearrangement experiment 18
11.4 Acyl chloride intermediate experiment 19
11.5 Synthesis of Co and its reactivity 21
12. Characterization data of products **24**
13. Reference **31**
14. Spectra of compounds **32**

1 General remarks

All chemicals and reagents were obtained from Macklin, Bidepharm and Sigma-Aldrich, and were used without further purification. All solvents were dried by standard techniques and distilled prior to use. Column chromatography was performed on silica gel (200-300 meshes) using petroleum ether (bp. 60~90 °C), ethyl acetate and acetone as eluent. ^{1}H and ^{13}C NMR spectra were taken on Bruker AVANCE III 400 MHz or 700 MHz spectrometers and spectral data were reported in ppm relative to tetramethylsilane (TMS) as the internal standard and $CDCl_3$ or DMSO-D_6 as solvent. All coupling constants (*J*) are reported in Hz with the following abbreviations: s = singlet, d = doublet, dd = double doublet, t = triplet, dt = double triplet, q = quatriplet, m = multiplet, br = broad. Gas chromatography (GC) analyses were performed on an Agilent HP-7890A instrument with a FID detector and HP-5 capillary column (polydimethylsiloxane with 5% phenyl groups, 30 m, 0.32 mm i.d. 0.25 μm film thickness) using argon as carrier gas. Gas chromatography mass spectrometer (GC-MS) analyses were performed on a Shimadzu QP2020 NX instrument. High resolution mass spectra (HRMS) were recorded on Agilent 8890-7250 and Agilent Q-TOF 6540. Unless otherwise noted, all reactions were carried out under carbon monoxide (CO) or nitrogen atmosphere. Because of the high toxicity of carbon monoxide, all the reactions should be performed in an autoclave. The laboratory should be well equipped with a CO detector and alarm system.

2 Structures of *N*-Substituted Indoles and Ligands

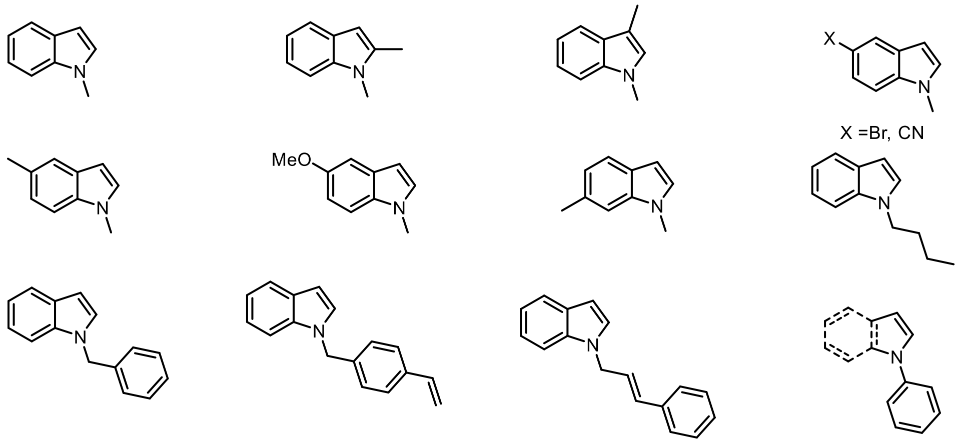

Examples of low yield

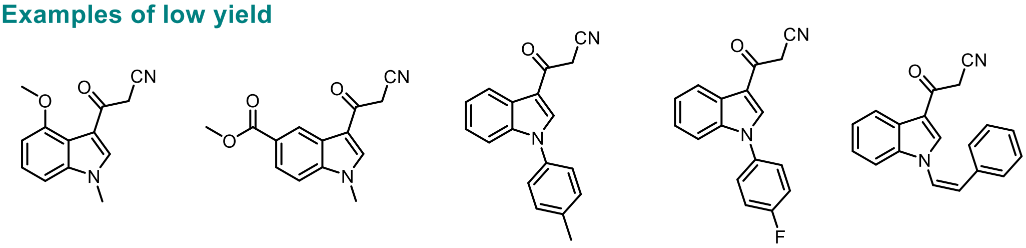

Examples of no reaction

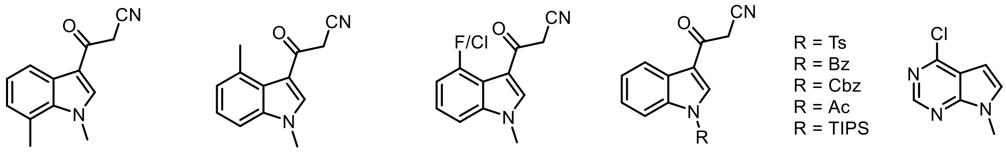

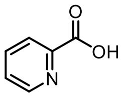

L1

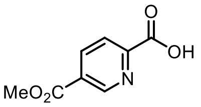

L2

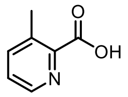

L3

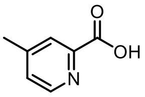

L4

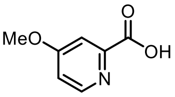

L5

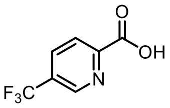

L6

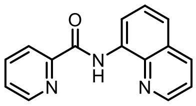

L9

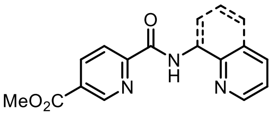

L10
L11

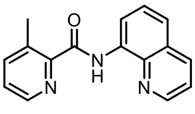

L12

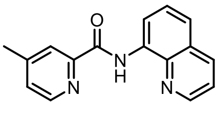

L13

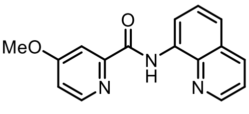

L14

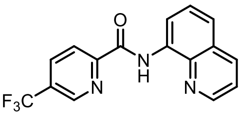

L15

3 General procedure for the synthesis of starting materials

Synthesis of *N*-alkyl-substituted indoles

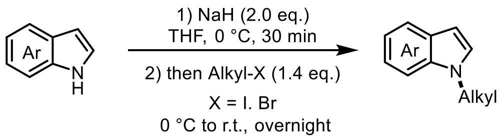

Add the substituted indole (3.0 mmol) to a fully dried Schlenk tube equipped with a magnetic stir bar and then dissolve it in THF (10 mL). The reaction mixture was cooled to 0 ºC, followed by addition of NaH (60%, 2.0 eq.). Keep stirring at 0 ºC Celsius for 30 minutes. Then, the alkyl halide was slowly added to the Schlenk tube. The reaction mixture allowed to warm to room temperature (Some fewer active halides can be heated appropriately) and stir overnight. Upon completion, add water to quench the reaction, then extract with ethyl acetate. The organic phase was dried with anhydrous sodium sulfate, the solvent was removed under vacuum and then purified by silica gel column chromatography (PE to PE/EA = 50:1).

Synthesis of *N*-aryl-substituted indoles

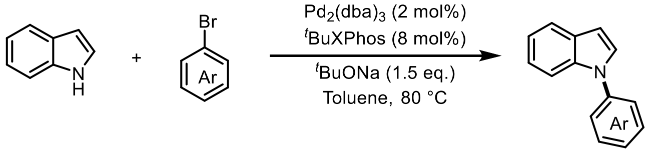

To an oven-dried 20 mL Schlenk tube was added $Pd_2(dba)_3$ (55 mg, 0.06 mmol, 2.0 mol%) and ^{t}BuXPhos (102 mg, 0.24 mmol, 8.0 mol%). The Schlenk tube was introduced in a nitrogen-filled glovebox, ^{t}BuONa (432 mg, 4.5 mmol, 1.5 equiv.), Toluene (2 mL), and a magnetic stir bar were added. The reaction tube was sealed with a rubber plug, removed from the glovebox and stirred at 80 °C for 2-12 h. Upon completion, add water to quench the reaction, then extract with ethyl acetate. The organic phase was dried with anhydrous sodium sulfate, the solvent was removed under vacuum and then purified by silica gel column chromatography (PE to PE/EA = 50:1).

(Z)-1-Styryl-1*H*-indole (S1)

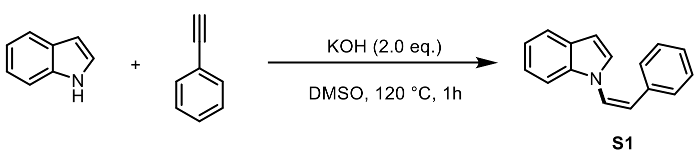

S1

To a 20 mL Schlenk tube equipped with a magnetic stir bar was added indole (3.0 mmol, 1.0 equiv.), KOH (6.0 mmol, 2.0 equiv.) and phenylacetylene (3.6 mmol, 1.2 equiv.), DMSO (2 mL).

The reaction tube is sealed with a rubber plug attached to a balloon, which is used to regulate the air pressure, stirred at 120 °C for 1 h. Upon completion, the solvent was removed under vacuum and then purified by silica gel column chromatography (PE to PE/EA = 50:1).

Synthesis of 3-cyclopropyl-1-methyl-1*H*-indole (S7)[1]

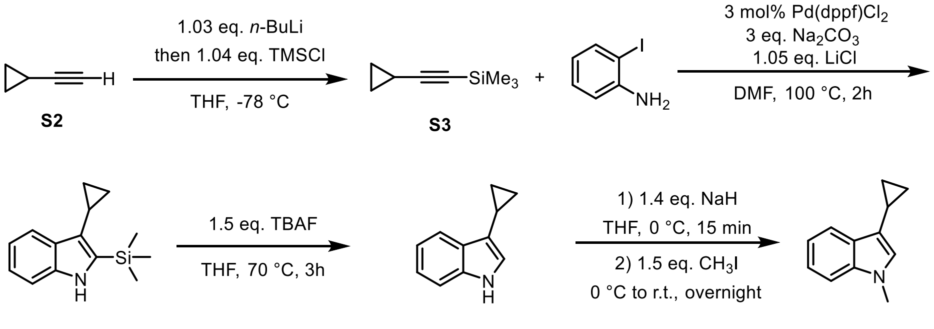

(Cyclopropylethynyl)trimethylsilane S3: Synthesis was conducted by reported procedure. To a stirring solution of cyclopropylacetylene (1.44 g, 21.8 mmol, 1 equiv.) in dry THF (7 mL) at -78 ºC was added n-BuLi (8.95 mL of 2.5 M in THF, 22.4 mmol, 1.03 equiv.) dropwise via syringe. The solution was allowed to stir for 30 minutes at -78 ºC, after which TMS–Cl (freshly distilled from CaH_2, 2.90 mL, 22.8 mmol, 1.04 equiv.) was added dropwise and the solution was stirred at -78 ºC for 1 hour. The reaction was then allowed to warm to room temperature, diluted with diethyl ether, and filtered through a pad of Na_2SO_4 layered on silica gel, eluting 1:4 Et_2O : pentane. The filtrate was concentrated in vacuo and then carried on crude.

3-Cyclopropyl-2-(trimethylsilyl)-1*H*-indole S4: To an oven-dried Schlenk tube was added Pd(dppf)Cl_2 (131 mg, 0.18 mmol, 3.0 mol%), 2-iodoaniline (1095 mg, 5.0 mmol, 1.0 equiv.) and Na_2CO_3 (1590 mg, 15 mmol). The Schlenk tube was introduced in a nitrogen-filled glovebox, LiCl (220 mg, 6.3 mmol, 1.05 equiv.), **S3** (1520 mg, 6.0 mmol, 1.2 equiv.) anhydrous DMF (20 mL), and a magnetic stir bar were added. The reaction tube was sealed with a rubber plug, removed from the glovebox and stirred at 100 °C for 2-4 h. Upon completion, add water to quench the reaction, then extract with ethyl acetate. The organic phase was dried with anhydrous sodium sulfate, the solvent was removed under vacuum and then purified by silica gel column chromatography (PE to PE/EA = 30:1).

3-Cyclopropyl-1*H*-indole S5: To a 20 mL Schlenk tube equipped with a magnetic stir bar was added S4 (1.0 equiv.) and TBAF (1.5 equiv.). The reaction tube was sealed with a rubber plug, stirred at 70 °C for 1-2 h. Upon completion, the solvent was removed under vacuum and then purified by

silica gel column chromatography (PE to PE/EA = 50:1).

3-cyclopropyl-1-methyl-1H-indole S6: This step is like the synthesis of *N*-alkyl-substituted indoles.

3-Cyclopropyl-2-(trimethylsilyl)-1*H*-indole (S4)

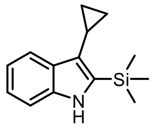

The title compound was purification by column chromatography on silica gel (petroleum ether) as a yellow solid. **^{1}H NMR (700 MHz, $CDCl_3$)** δ 7.82 (s, NH, 1H), 7.69 (dd, *J* = 7.7, 1.4 Hz, 1H), 7.26 (dt, *J* = 7.7, 0.7 Hz, 1H), 7.14 – 7.08 (m, 1H), 7.05 – 6.99 (m, 1H), 1.94 – 1.87 (m, 1H), 0.96 – 0.86 (m, 2H), 0.80 – 0.75 (m, 2H), 0.37 (s, 4H).**^{13}C NMR (175 MHz, $CDCl_3$)** δ 138.5, 135.8, 129.6, 127.0, 122.8, 120.3, 119.7, 111.5, 8.3, 7.0, 0.0.

3-Cyclopropyl-1*H*-indole (S5)

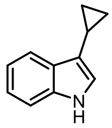

The title compound was purification by column chromatography on silica gel (petroleum ether/EtOAc = 50:1) as a yellow solid (when the ambient temperature is relatively high, the product may be a liquid). **^{1}H NMR (700 MHz, $CDCl_3$)** δ 7.82 (s, NH, 1H), 7.74 (d, *J* = 7.7 Hz, 1H), 7.32 (d, *J* = 7.7 Hz, 1H), 7.20 – 7.17 (m, 1H), 7.12 (t, *J* = 7.0 Hz, 1H), 6.87 (d, *J* = 2.1 Hz, 1H), 2.08 – 1.89 (m, 1H), 0.91 – 0.83 (m, 2H), 0.68 – 0.59 (m, 2H).**^{13}C NMR (175 MHz, $CDCl_3$)** δ 136.4, 128.2, 122.1, 120.4, 119.2, 119.2, 119.2, 111.1, 6.2, 6.0. **MS (EI)**： 157.

4. Optimization of reaction conditions

Table 1: The effect of Ligands.

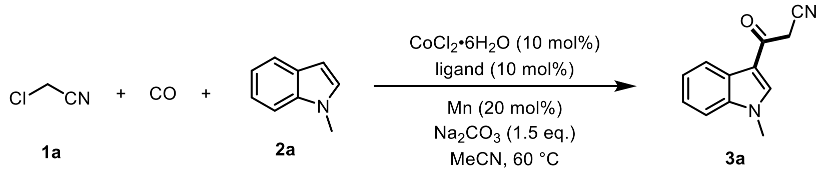

Entry	Ligand	Yield (%)[a]
1	**L1**	0
2	**L2**	31
3	**L3**	0
4	**L4**	0
5	**L5**	0
6	**L6**	30
7	**L7**	0
8	**L8**	0
9	**L9**	5
10	**L10**	9
11	**L11**	10
12	**L12**	14
13	**L13**	0
14	**L14**	0
15	**L15**	13

Reaction conditions: **1a** (1.5 eq.), **2a** (0.3 mmol), $CoCl_2 \cdot 6H_2O$ (10 mol%), **Ligand** (10 mol%), Mn (20 mol%), Na_2CO_3 (1.5 eq.), MeCN (1.5 mL), **CO** (40 bar), 60 °C, 12 h. [a]Yields were determined by GC with dodecane as an internal standard.

Table 2: The effect of temperature.

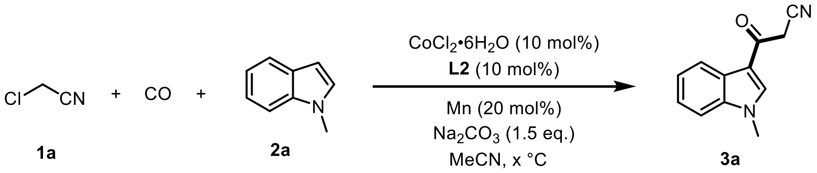

Entry	Temperature (x °C)	Yield (%)[a]
1	50	8
2	60	31
**3**	**80**	**62**
4	100	62

Reaction conditions: **1a** (1.5 eq.), **2a** (0.3 mmol), $CoCl_2 \cdot 6H_2O$ (10 mol%), **L2** (10 mol%), Mn (20 mol%), Na_2CO_3 (1.5 eq.), MeCN (1.5 mL), **CO** (40 bar), x °C, 12 h. [a]Yields were determined by GC with dodecane as an internal standard.

Table 3: The effect of solvents.

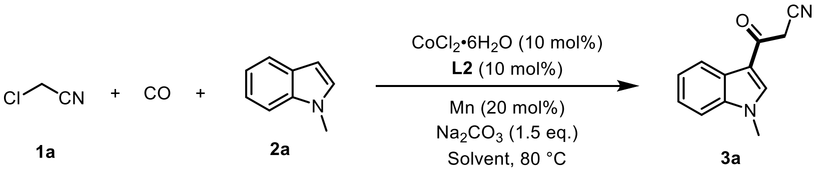

Entry	Reductant (x mol%)	Yield (%)[a]
1	Dioxane	0
2	THF	14
3	DMF	0
4	$PhCF_3$	0
5	DCE	0
6	DCM	0
8	DME	25

Reaction conditions: **1a** (1.5 eq.), **2a** (0.3 mmol), $CoCl_2 \cdot 6H_2O$ (10 mol%), **L2** (10 mol%), Mn (20 mol%), Na_2CO_3 (1.5 eq.), solvent (1.5 mL), **CO** (40 bar), 80 °C, 12 h. [a]Yields were determined by GC with dodecane as an internal standard.

Table 4: The effect of Co salts.

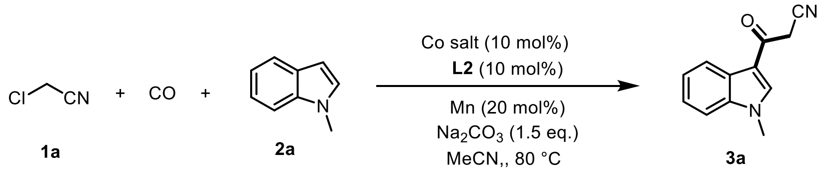

Entry	Metal salts	Yield (%)[a]
1	Co(acac)3	0
**2**	**Co(acac)2**	**73**
3	CoCl2•6H2O	62
4	Co(OAc) 2•4H2O	68
5	Co(OAc) 2	69
6	CoCl2	69
7	CoBr2	49
8	w/o Mn	0
9	w/o [Co]	0

Reaction conditions: **1a** (1.5 eq.), **2a** (0.3 mmol), Co salt (10 mol%), **L2** (10 mol%), Mn (20 mol%), Na_2CO_3 (1.5 eq.), solvent (1.5 mL), **CO** (40 bar), 80 °C, 12 h. [a]Yields were determined by GC with dodecane as an internal standard.

Table 5: The effect of equivalent of **1a**.

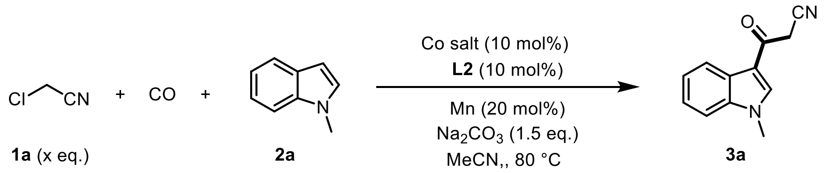

Entry	X equivalent	Yield (%)[a]
1	1.5	73
2	2.0	79
3	2.5	81
4	3.0	85
6	4.0	95(91)[b]

Reaction conditions: **1a** (x eq.), **2a** (0.3 mmol), $Co(acac)_2$ (10 mol%), **L2** (10 mol%), Mn (20 mol%), Na_2CO_3 (1.5 eq.), MeCN (1.5 mL), **CO** (40 bar), 80 °C, 12 h. [a]Yields were determined by GC with dodecane as an internal standard. [b]Isolated yield.

Table 6: The effect of the pressure of **CO**.

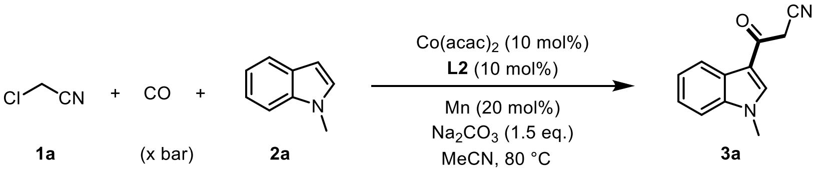

Entry	pressure (x bar)	Yield (%)[a]
1	40	95
**2**	**30**	**95**
3	20	59
4	10	27
5	5	8

Reaction conditions: **1a** (4.0 eq.), **2a** (0.3 mmol), Co(acac)$_2$ (10 mol%), **L2** (10 mol%), Mn (20 mol%), Na_2CO_3 (1.5 eq.), MeCN (1.5 mL), **CO** (x bar), 80 °C, 12 h. [a]Yields were determined by GC with dodecane as an internal standard.

5. General procedure for *N*-protected indoles

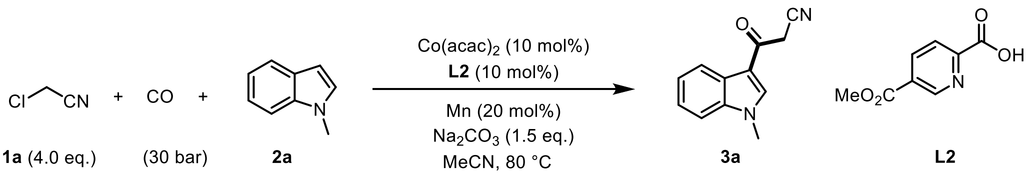

A 4 mL screw-cap vial was charged with Co(acac)$_2$ (10 mol%, 7.7 mg), **L2** (10 mol%, 5.4 mg), Mn (20 mol%, 4.4 mg), Na_2CO_3 (0.45 mmol, 47.7 mg) and an oven-dried stirring bar. The vial was closed with a Teflon septum and cap and connected to the atmosphere via a needle. Then *N*-methylindole **2a** (0.3 mmol, 39.3 μL), $ClCH_2CN$ (1.2 mmol, 80.0 μL), MeCN (1.5 mL) was added with a syringe under N_2 atmosphere, the vial was moved to an alloy plate and put into a Parr 4560 series autoclave (300 mL) under N_2 atmosphere. At room temperature, the autoclave flushed with N_2 three times and CO three times and charged with 30 bar CO. The autoclave was placed on a heating plate equipped with a magnetic stirrer. The reaction mixture was heated to 80 °C for 12 h. After the reaction was completed, the crude mixture was filtered and concentrated under vacuum. The crude product was purified by column chromatography on silica gel to afford the corresponding compounds.

6. General procedure for unprotected indoles

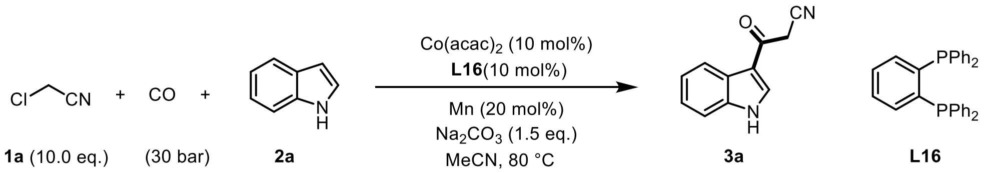

A 4 mL screw-cap vial was charged with Co(acac)$_2$ (10 mol%, 7.7 mg), **L16** (10 mol%, 13.4 mg), Mn (20 mol%, 4.4 mg), Na_2CO_3 (0.45 mmol, 47.7 mg) and an oven-dried stirring bar. Then *N*-methylindole **2a** (0.3 mmol, 39.3 μL), $ClCH_2CN$ (1.2 mmol, 80.0 μL), MeCN (1.5 mL) was added with a syringe under N_2 atmosphere, the vial was moved to an alloy plate and put into a Parr 4560 series autoclave (300 mL) under N_2 atmosphere. At room temperature, the autoclave flushed with N_2 three times and CO three times and charged with 30 bar CO. The autoclave was placed on a heating plate equipped with a magnetic stirrer. The reaction mixture was heated to 80 °C for 12 h. After the reaction was completed, the crude mixture was filtered and concentrated under vacuum. The crude product was purified by column chromatography on silica gel to afford the corresponding compounds.

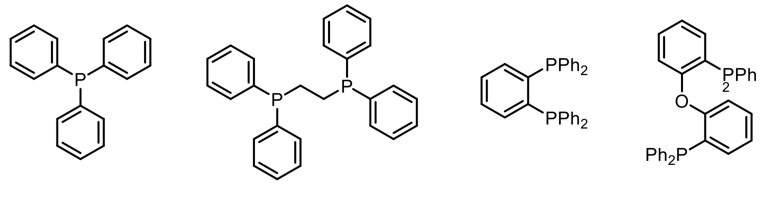

L14 **L15** **L16** **L17**

7. General procedure for alkyl iodides

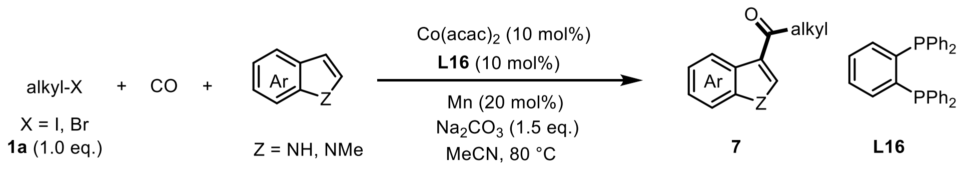

A 4 mL screw-cap vial was charged with Co(acac)$_2$ (10 mol%, 7.7 mg), **L16** (10 mol%, 13.4 mg), Mn (20 mol%, 4.4 mg), Na_2CO_3 (0.45 mmol, 47.7 mg) and an oven-dried stirring bar. Then *N*-methylindole **2a** (0.45 mmol, 59.0 μL), alkyl iodide (0.4 mmol), MeCN (1.5 mL) was added with a syringe under N_2 atmosphere, the vial was moved to an alloy plate and put into a Parr 4560 series autoclave (300 mL) under N_2 atmosphere. At room temperature, the autoclave flushed with N_2 three

times and CO three times and charged with 30 bar CO. The autoclave was placed on a heating plate equipped with a magnetic stirrer. The reaction mixture was heated to 80 °C for 12 h. After the reaction was completed, the crude mixture was filtered and concentrated under vacuum. The crude product was purified by column chromatography on silica gel to afford the corresponding compounds.

8. Scale-up reaction of 3a

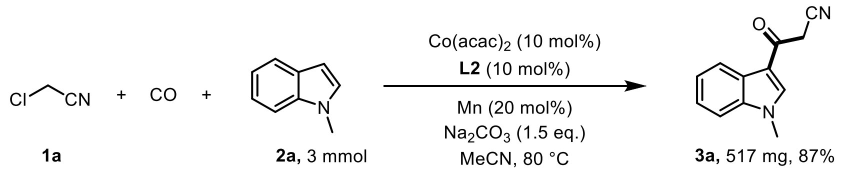

A 25 mL screw-cap vial was charged with $Co(acac)_2$ (10 mol%), L17 (10 mol%), Mn (20 mol%), Na_2CO_3 (45 mmol) and an oven-dried stirring bar. Then *N*-methylindole **2a** (3.0 mmol, 393.0 μL), $ClCH_2CN$ (12 mmol, 800.0 μL), MeCN (15 mL) was added with a syringe under N_2 atmosphere, the vial was moved to an alloy plate and put into a Parr 4560 series autoclave (300 mL) under N_2 atmosphere. At room temperature, the autoclave flushed with N_2 three times and CO three times and charged with 30 bar CO. The autoclave was placed on a heating plate equipped with a magnetic stirrer. The reaction mixture was heated to 80 °C for 24 h. After the reaction was completed, the crude mixture was filtered and concentrated under vacuum. The crude product was purified by column chromatography on silica gel to afford the corresponding compound **3a** at 87% yield.

9. late-stage modification of 4a

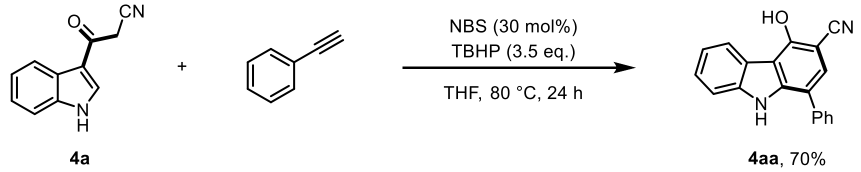

A 10 mL round bottom flask with a magneton was charged with 3-(1*H*-indol-3-yl)-3-oxopropanenitrile **4a** (0.3 mmol, 1 equiv.) and NBS (0.09 mmol, 0.3 equiv) under air at room temperature, and then phenylacetylene (0.6 mmol, 2 equiv), *tert*-butyl hydroperoxide (1.05 mmol, 3.5 equiv) and tetrahydrofuran (1.5 mL) were added. After the mixture was stirred at 80 °C for 24

h, the resulting residue was mixed with silica gel and concentrated. The resulting mixture was purified by silica gel column chromatography on silica gel with petroleum ether/EtOAc (15:1) as eluent to give the desired product 4-hydroxy-1-phenyl-9*H*-carbazole-3-carbonitrile **4aa** (59 mg, 70%).

4-Hydroxy-1-phenyl-9*H*-carbazole-3-carbonitrile (4aa)[2]

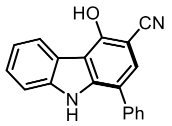

The title compound was prepared following the general procedure, purification by column chromatography on silica gel (petroleum ether/ EtOAc = 15:1) yielded (59 mg, 70%) as a yellow solid. **^{1}H NMR (700 MHz, $CDCl_3$)** δ 11.58 (s, NH, 1H), 11.17 (s, OH, 1H), 8.33 (d, *J* = 7.7 Hz, 1H), 7.69 (d, *J* = 7.7 Hz, 2H), 7.64 – 7.55 (m, 4H), 7.51 (d, *J* = 2.1 Hz, 1H), 7.48 – 7.40 (m, 3H), 7.27 (t, *J* = 7.7 Hz, 1H). **^{13}C NMR (175 MHz, $CDCl_3$)** δ 156.3, 141.5, 140.1, 137.4, 129.5, 129.4, 128.9, 128.0, 126.1, 122.8, 122.0, 120.5, 119.4, 119.0, 113.2, 112.2, 90.5. **HRMS (ESI-TOF) m/z:** $[M+H]^+$: Calcd. for $C_{19}H_{13}N_2O^+$: 285.1023. Found: 285.1022.

10. Cyclic voltammetry

Cyclic voltammograms were recorded with Admiral Squidsta Plus workstation potentiostat at room temperature in MeCN. ^{n}Bu$_4$NPF$_6$ (0.1 M) was used as the supporting electrolyte, and a standard platinum electrode was used as the working electrode. The auxiliary electrode was a glass carbon electrode. All potentials are referenced against the Ag/AgCl redox couple. The scan rate was 100 mV/s.[3]

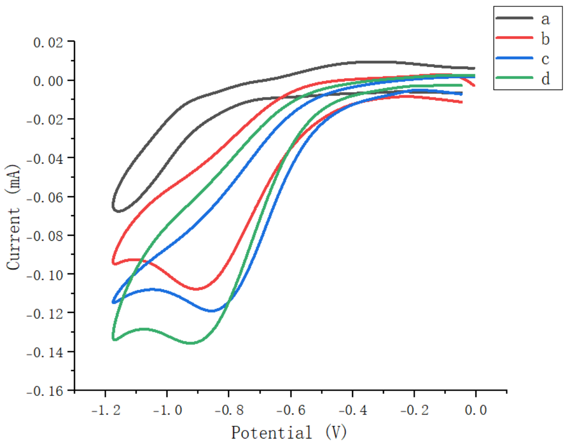

Figure S1: Cyclic voltammograms recorded on a Pt electrode at 100 mV/s in: (a) MeCN containing 0.1 M of ^{n}Bu$_4$NPF$_6$, black; (b) solution (a) with 0.2 mM of chloroacetonitrile added, red; (c) solution

(a) with 0.05 mM of $Co(OAc)_2 \cdot 4H_2O$ and **L2** added, blue; (d) solution (c) with 0.2 mM of chloroacetonitrile added, green.

11. Mechanism experiments

11.1 Radical inhibition experiment and radical capture experiment

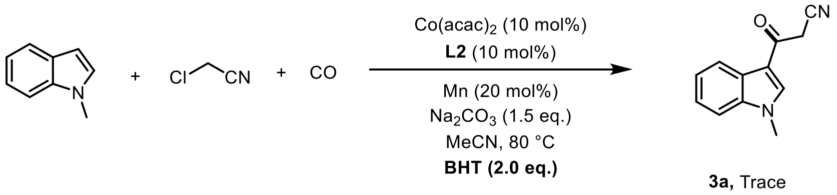

3a, Trace

A 4 mL screw-cap vial was charged with $Co(acac)_2$ (10 mol%, 7.7 mg), **L2** (10 mol%, 5.4 mg), Mn (20 mol%, 4.4 mg), Na_2CO_3 (0.45 mmol, 47.7 mg), BHT (0.6 mmol, 132 mg) and an oven-dried stirring bar. The vial was closed with a Teflon septum and cap and connected to the atmosphere via a needle. Then *N*-methylindole **2a** (0.3 mmol, 39.3 μL), $ClCH_2CN$ (1.2 mmol, 80.0 μL), MeCN (1.5 mL) was added with a syringe under N_2 atmosphere, the vial was moved to an alloy plate and put into a Parr 4560 series autoclave (300 mL) under N_2 atmosphere. At room temperature, the autoclave flushed with N_2 three times and CO three times and charged with 30 bar CO. The autoclave was placed on a heating plate equipped with a magnetic stirrer. The reaction mixture was heated to 80 °C for 12 h. After the reaction was completed, determined by GC with dodecane as an internal standard.

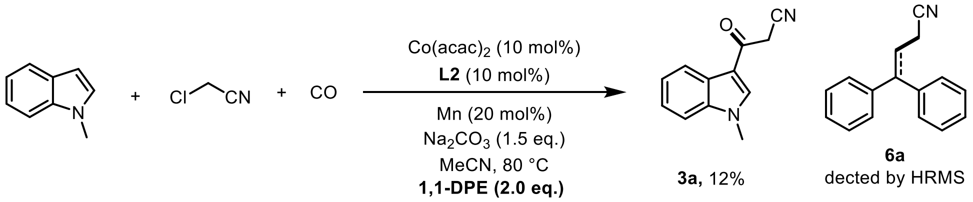

3a, 12% **6a** dected by HRMS

A 4 mL screw-cap vial was charged with $Co(acac)_2$ (10 mol%, 7.7 mg), **L2** (10 mol%, 5.4 mg), Mn (20 mol%, 4.4 mg), Na_2CO_3 (0.45 mmol, 47.7 mg), and an oven-dried stirring bar. The vial was closed with a Teflon septum and cap and connected to the atmosphere via a needle. Then *N*-methylindole **2a** (0.3 mmol, 39.3 μL), $ClCH_2CN$ (1.2 mmol, 80.0 μL), 1,1-DPE (0.6 mmol, 108 mg), MeCN (1.5 mL) was added with a syringe under N_2 atmosphere, the vial was moved to an alloy plate and put into a Parr 4560 series autoclave (300 mL) under N_2 atmosphere. At room temperature, the autoclave flushed with N_2 three times and CO three times and charged with 30 bar CO. The

autoclave was placed on a heating plate equipped with a magnetic stirrer. The reaction mixture was heated to 80 °C for 12 h. After the reaction was completed, determined by GC with dodecane as an internal standard.

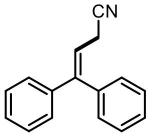

HRMS (ESI-TOF) m/z: $[M+H]^+$ Calcd. for $C_{16}H_{14}N$ 200.1121; found: 200.1119.

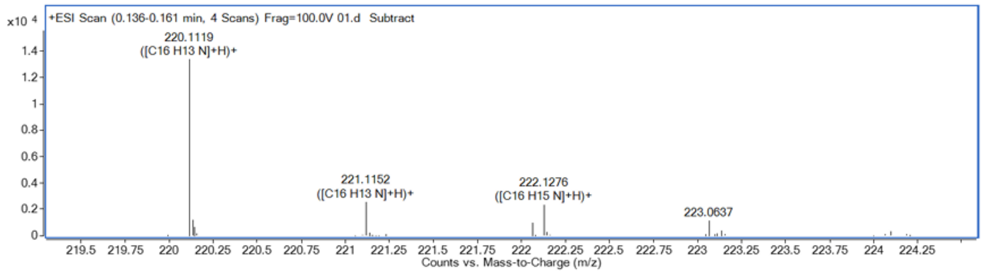

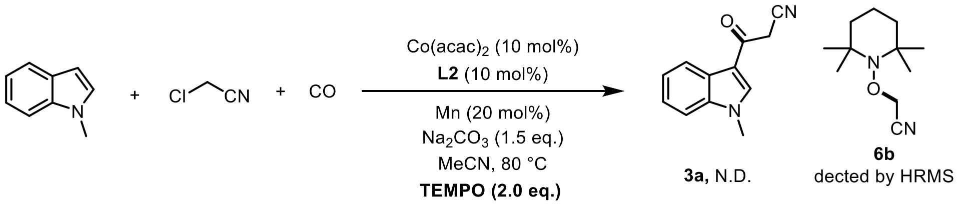

A 4 mL screw-cap vial was charged with $Co(acac)_2$ (10 mol%, 7.7 mg), **L2** (10 mol%, 5.4 mg), Mn (20 mol%, 4.4 mg), Na_2CO_3 (0.45 mmol, 47.7 mg), TEMPO (0.6 mmol, 93.6 mg), and an oven-dried stirring bar. The vial was closed with a Teflon septum and cap and connected to the atmosphere via a needle. Then *N*-methylindole **2a** (0.3 mmol, 39.3 μL), $ClCH_2CN$ (1.2 mmol, 80.0 μL), MeCN (1.5 mL) was added with a syringe under N_2 atmosphere, the vial was moved to an alloy plate and put into a Parr 4560 series autoclave (300 mL) under N_2 atmosphere. At room temperature, the autoclave flushed with N_2 three times and CO three times and charged with 30 bar CO. The autoclave was placed on a heating plate equipped with a magnetic stirrer. The reaction mixture was heated to 80 °C for 12 h. After the reaction was completed, determined by GC with dodecane as an internal standard.

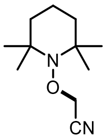

HRMS (ESI-TOF) m/z: $[M+H]^+$ Calcd. for $C_{11}H_{21}N_2O$ 197.1649; found: 197.1654.

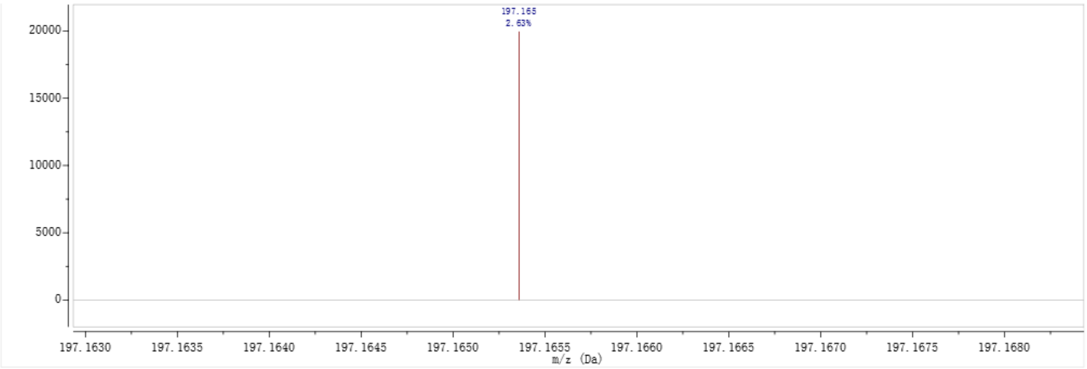

11.2 Radical addition experiments

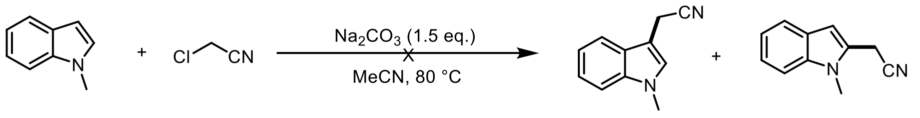

6c **6d**

A 4 mL screw-cap vial was charged Na_2CO_3 (0.45 mmol, 47.7 mg) and an oven-dried stirring bar. The vial was closed with a Teflon septum and cap and connected to the atmosphere via a needle. Then *N*-methylindole **2a** (0.3 mmol, 39.3 μL), $ClCH_2CN$ (1.2 mmol, 80.0 μL), MeCN (1.5 mL) was added with a syringe under N_2 atmosphere, the vial was moved to an alloy plate and put into a Parr 4560 series autoclave (300 mL) under N_2 atmosphere. At room temperature, the autoclave flushed with N_2 three times then charged with 30 bar N_2. The autoclave was placed on a heating plate equipped with a magnetic stirrer. The reaction mixture was heated to 80 °C for 12 h.

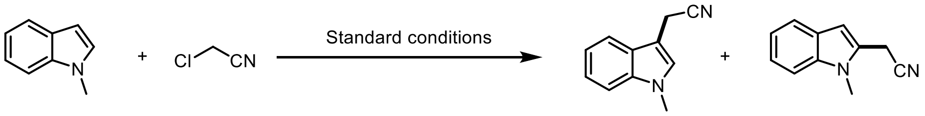

6c, 8% **6d**, 70%

A 4 mL screw-cap vial was charged with Co(acac)$_2$ (10 mol%, 7.7 mg), **L16** (10 mol%, 13.4 mg), Mn (20 mol%, 4.4 mg), Na_2CO_3 (0.45 mmol, 47.7 mg) and an oven-dried stirring bar. The vial was closed with a Teflon septum and cap and connected to the atmosphere via a needle. Then *N*-methylindole **2a** (0.3 mmol, 39.3 μL), $ClCH_2CN$ (1.2 mmol, 80.0 μL), MeCN (1.5 mL) was added with a syringe under N_2 atmosphere, the vial was moved to an alloy plate and put into a Parr 4560 series autoclave (300 mL) under N_2 atmosphere. At room temperature, the autoclave flushed with N_2 three times then charged with 30 bar N_2. The autoclave was placed on a heating plate equipped with a magnetic stirrer. The reaction mixture was heated to 80 °C for 12 h. After the reaction was completed, the crude product was purified by column chromatography on silica gel to afford the

corresponding compounds.

2-(1-Methyl-1*H*-indol-3-yl)acetonitrile (6c)

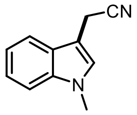

The title compound was prepared following the general procedure, purification by column chromatography on silica gel (petroleum ether/EtOAc = 10:1) yielded (the average yield for four reactions, 4.0 mg, 8%) as a white solid. **^{1}H NMR (700 MHz, $CDCl_3$)** δ 7.56 (dt, *J* = 7.7, 0.7 Hz, 1H), 7.33 (d, *J* = 8.4 Hz, 1H), 7.29 – 7.26 (m, 1H), 7.20 – 7.16 (m, 1H), 7.07 (s, 1H), 3.81 (s, 1H), 3.77 (s, 3H). **^{13}C NMR (175 MHz, $CDCl_3$)** δ 137.1, 127.4, 126.4, 122.4, 119.7, 118.3, 118.2, 109.6, 102.9, 32.8, 14.3. **GC-MS (EI):** 170.

2-(1-Methyl-1*H*-indol-2-yl)acetonitrile (6d)

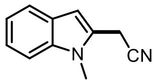

The title compound was prepared following the general procedure, purification by column chromatography on silica gel (petroleum ether/EtOAc = 10:1) yielded (35.5 mg, 70%) as a white solid. **^{1}H NMR (700 MHz, $CDCl_3$)** δ 7.57 (dd, *J* = 7.7, 1.4 Hz, 1H), 7.33 – 7.27 (m, 1H), 7.26 – 7.22 (m, 1H), 7.16 – 7.11 (m, 1H), 6.51 (s, 1H), 3.82 (s, 1H), 3.70 (s, 3H). **^{13}C NMR (175 MHz, $CDCl_3$)** δ 137.9, 127.7, 127.1, 122.3, 120.7, 120.2, 116.1, 109.3, 102.3, 29.8, 16.7. **GC-MS (EI):** 170.

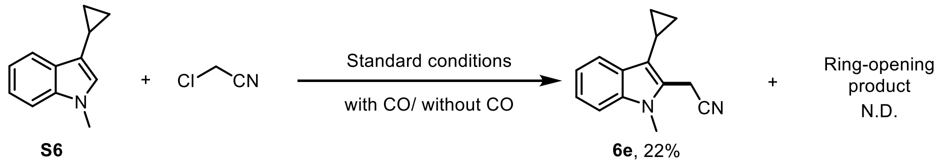

A 4 mL screw-cap vial was charged with $Co(acac)_2$ (10 mol%, 7.7 mg), **L16** (10 mol%, 13.4 mg), Mn (20 mol%, 4.4 mg), Na_2CO_3 (0.45 mmol, 47.7 mg), and an oven-dried stirring bar. The vial was closed with a Teflon septum and cap and connected to the atmosphere via a needle. Then 3-cyclopropyl-1-methyl-1*H*-indole (0.3 mmol, 51.0 mg), $ClCH_2CN$ (1.2 mmol, 80.0 μL), MeCN (1.5 mL) was added with a syringe under N_2 atmosphere, the vial was moved to an alloy plate and put into a Parr 4560 series autoclave (300 mL) under N_2 atmosphere. At room temperature, the autoclave flushed with N_2 three times then charged with 30 bar N_2 (or CO). The autoclave was placed on a heating plate equipped with a magnetic stirrer. The reaction mixture was heated to 80 °C for 12 h. After the reaction was completed, the crude product was purified by column chromatography on silica gel to afford the corresponding compounds.

2-(3-Cyclopropyl-1-methyl-1*H*-indol-2-yl)acetonitrile (6e)

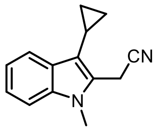

The title compound was prepared following the general procedure, purification by column chromatography on silica gel (petroleum ether/EtOAc = 10:1) yielded (13.8 mg, 22%) as a yellow oil. **^{1}H NMR (700 MHz, CDCl$_3$)** δ 7.73 (dt, *J* = 7.7, 0.7 Hz, 1H), 7.32 – 7.29 (m, 1H), 7.26 – 7.23 (m, 1H), 7.16 – 7.11 (m, 1H), 4.02 (s, 2H), 3.77 (s, 2H), 1.79 (tt, *J* = 8.4, 5.6 Hz, 1H), 1.03 – 0.95 (m, 2H), 0.70 – 0.59 (m, 2H).**^{13}C NMR (175 MHz, CDCl$_3$)** δ 180.4, 137.5, 136.0, 126.2, 124.2, 123.5, 122.4, 115.0, 114.3, 110.0, 33.9, 29.7. **GC-MS (EI):** 210.

11.3 Carbon ion rearrangement experiment

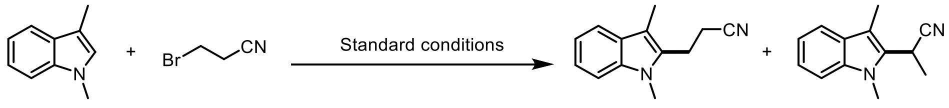

S7 **1b** **6f** dected by GC-MS **6g** dected by GC-MS

A 4 mL screw-cap vial was charged with Co(acac)$_2$ (10 mol%, 7.7 mg), **L16** (10 mol%, 13.4 mg), Mn (20 mol%, 4.4 mg), Na_2CO_3 (0.45 mmol, 47.7 mg), and an oven-dried stirring bar. The vial was closed with a Teflon septum and cap and connected to the atmosphere via a needle. Then 1,3-dimethyl-1*H*-indole **S7** (0.3 mmol, 51.0 mg), **1b** (1.2 mmol, 80.0 μL), MeCN (1.5 mL) was added with a syringe under N_2 atmosphere, the vial was moved to an alloy plate and put into a Parr 4560 series autoclave (300 mL) under N_2 atmosphere. At room temperature, the autoclave flushed with N_2 three times then charged with 30 bar N_2. The autoclave was placed on a heating plate equipped with a magnetic stirrer. The reaction mixture was heated to 80 °C for 12 h. After the reaction was completed, the reaction solution was tested using GC-MS. **GC-MS (EI):** 198.

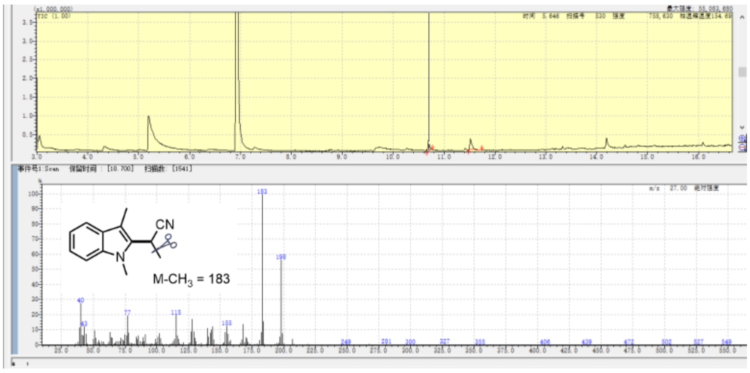

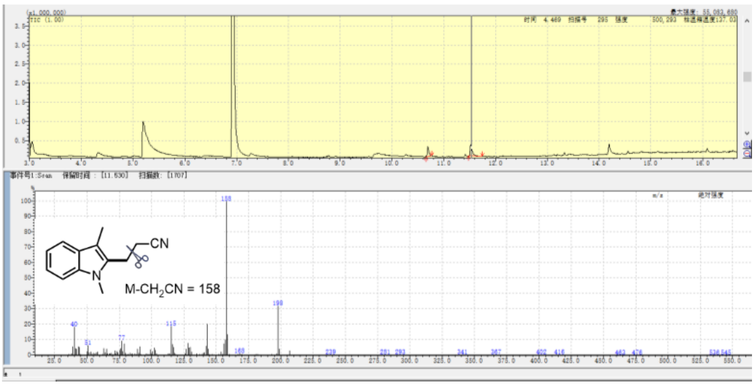

11.4 Acyl chloride intermediate experiment

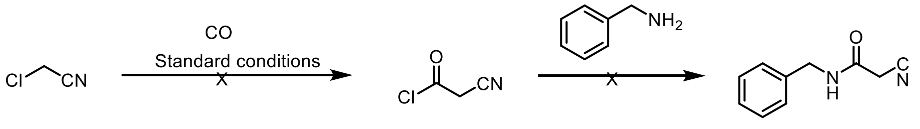

6h, HRMS N.D. **6i**, N.D.

A 4 mL screw-cap vial was charged with $Co(acac)_2$ (10 mol%, 7.7 mg), **L16** (10 mol%, 13.4 mg), Mn (20 mol%, 4.4 mg), Na_2CO_3 (0.45 mmol, 47.7 mg), and an oven-dried stirring bar. The vial was closed with a Teflon septum and cap and connected to the atmosphere via a needle. Then $ClCH_2CN$ (0.3 mmol, 20.0 μL), MeCN (1.5 mL) was added with a syringe under N_2 atmosphere, the vial was moved to an alloy plate and put into a Parr 4560 series autoclave (300 mL) under N_2 atmosphere. At room temperature, the autoclave flushed with N_2 three times then charged with 30 bar CO. The autoclave was placed on a heating plate equipped with a magnetic stirrer. The reaction mixture was heated to 80 °C for 12 h. After the reaction was completed, the reaction vial was moved into a glove box filled with nitrogen. A small amount of reaction mixture was filtered for HRMS test, and the remaining reaction mixture was added with 2 equivalents of benzylamine and stirred at room temperature.

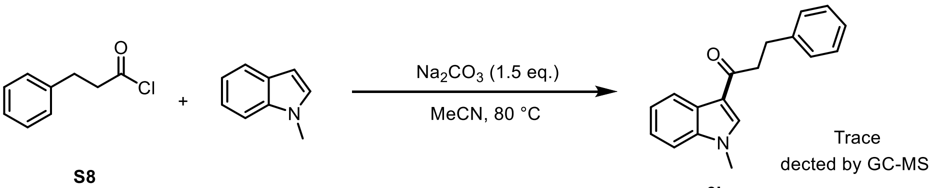

S8 **6j**

A 4 mL screw-cap vial was charged with Na_2CO_3 (0.45 mmol, 47.7 mg), and an oven-dried stirring bar. The vial was closed with a Teflon septum and cap and connected to the atmosphere via a needle. Then **S8** (0.3 mmol), 1-methyl-1H-indole (0.3 mmol) and MeCN (1.5 mL) were added with a syringe under N_2 atmosphere, the vial was moved to an alloy plate and put into a Parr 4560 series

autoclave (300 mL) under N_2 atmosphere. At room temperature, the autoclave flushed with N_2 three times then charged with 30 bar CO. The autoclave was placed on a heating plate equipped with a magnetic stirrer. The reaction mixture was heated to 80 °C for 12 h. After the reaction was completed, the reaction mixture was filtered for GC-MS test.

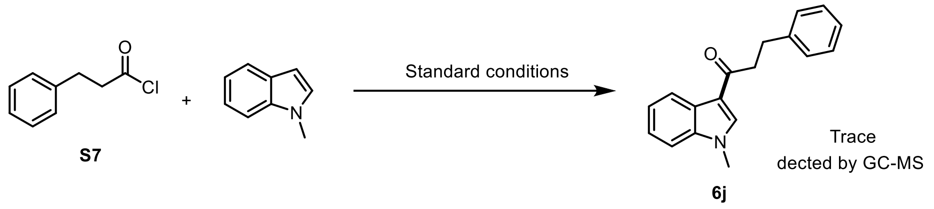

A 4 mL screw-cap vial was charged with Co(acac)$_2$ (10 mol%, 7.7 mg), **L16** (10 mol%, 13.4 mg), Mn (20 mol%, 4.4 mg), Na_2CO_3 (0.45 mmol, 47.7 mg), and an oven-dried stirring bar. The vial was closed with a Teflon septum and cap and connected to the atmosphere via a needle. Then **S8** (0.3 mmol), 1-methyl-1H-indole (0.3 mmol) and MeCN (1.5 mL) were added with a syringe under N_2 atmosphere, the vial was moved to an alloy plate and put into a Parr 4560 series autoclave (300 mL) under N_2 atmosphere. At room temperature, the autoclave flushed with N_2 three times then charged with 30 bar CO. The autoclave was placed on a heating plate equipped with a magnetic stirrer. The reaction mixture was heated to 80 °C for 12 h. After the reaction was completed, the reaction mixture was filtered for GC-MS test.

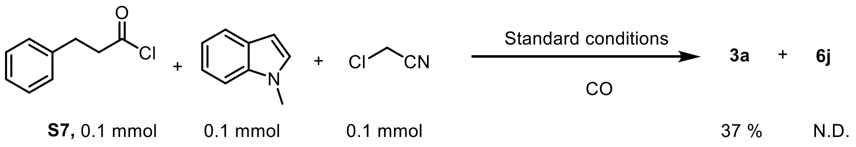

A 4 mL screw-cap vial was charged with Co(acac)$_2$ (10 mol%, 7.7 mg), **L16** (10 mol%, 13.4 mg), Mn (20 mol%, 4.4 mg), Na_2CO_3 (0.45 mmol, 47.7 mg), and an oven-dried stirring bar. The vial was closed with a Teflon septum and cap and connected to the atmosphere via a needle. Then **S8** (0.3 mmol), 1-methyl-1H-indole (0.1 mmol), $ClCH_2CN$ (0.1 mmol, 16 μL) and MeCN (1.5 mL) were added with a syringe under N_2 atmosphere, the vial was moved to an alloy plate and put into a Parr 4560 series autoclave (300 mL) under N_2 atmosphere. At room temperature, the autoclave flushed with N_2 three times then charged with 30 bar CO. The autoclave was placed on a heating plate equipped with a magnetic stirrer. The reaction mixture was test by GC.

11.5 Synthesis of Co and its reactivity

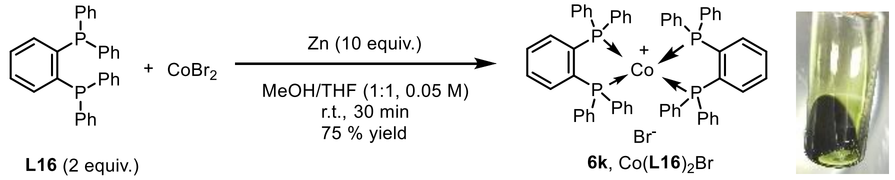

L16 (2 equiv.) **6k**, Co(**L16**)$_2$Br

In a N_2-filled glove-box, an oven-dried vial (8 mL) equipped with a magnetic stir bar was charged with **L16** (89.2 mg, 0.2 mmol, 1.0 equiv), $CoBr_2$ (21.8 mg, 0.1 mmol, 1.0 equiv), Zn (65.0 mg, 37 2.0 mmol, 10.0 equiv), THF (1 mL), MeOH (1 mL) and a stir bar. The mixture was stirred for 30 minutes at room temperature and then filtered through celite. The solvents were removed at room temperature under vacuum and the crude product was washed with hexane (5 mL). The residual solvent was removed at room temperature under vaccum, affording complex Co^{I}(**L16**)$_2$Br as a dark green solid solid (77.2 mg, 75%).[4]

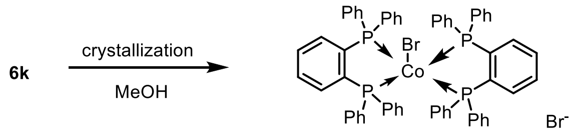

6l, Co(**L16**)$_2$Br$_2$•MeOH•H_2O

Dissolve 6k in methanol for crystallization; the dark green solution turns brownish-red, and brownish-red crystals **6l** were obtained.

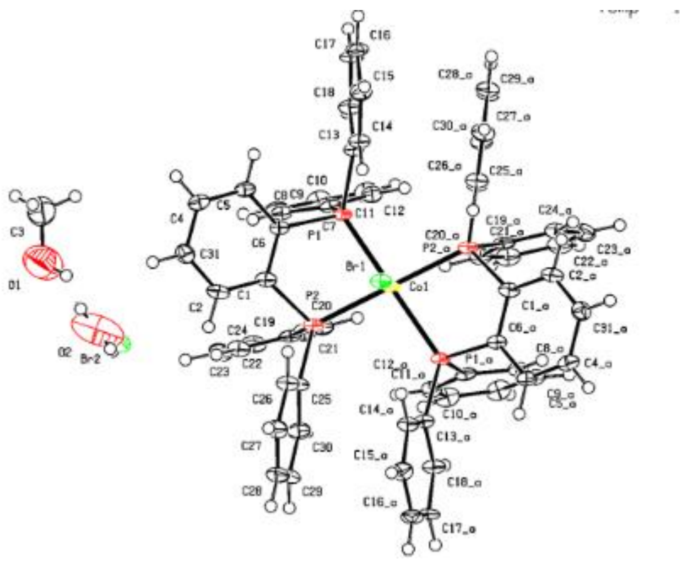

Formula	$C_{61}H_{54}Br_2Co_2O_2P_4$
$D_{calc.}$/ g cm^{-3}	1.489
μ/mm^{-1}	5.788
Formula Weight	596.86
Colour	clear brownish red
Shape	block
Size/mm^3	0.12×0.12×0.10
T/K	100.00(10)
Crystal System	monoclinic
Space Group	$P2/n$
a/Å	15.5390(4)
b/Å	9.5032(2)
c/Å	18.1818(4)
α/°	90
β/°	97.433(2)
γ/°	90
V/Å^3	2662.35(11)
Z	4
Z'	1
Wavelength/Å	1.54184
Radiation type	Cu K$_\alpha$
Θ_{min}/°	4.008
Θ_{max}/°	76.260
Measured Refl.	19195
Independent Refl.	5295
Reflections with I > 2(I)	4730
R_{int}	0.0807
Parameters	329
Restraints	0
Largest Peak	1.990
Deepest Hole	-1.603
GooF	1.049
wR_2 (all data)	0.2036
wR_2	0.1980
R_1 (all data)	0.0794
R_1	0.0735

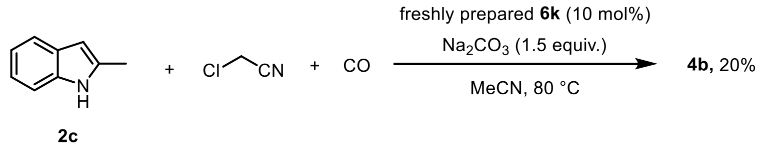

A 4 mL screw-cap vial was charged with 2-methyl-1*H*-indole **2c** (0.3 mmo), **6k** (10 mol%), Na_2CO_3 (0.45 mmol, 47.7 mg) and an oven-dried stirring bar. The vial was closed with a Teflon septum and cap and connected to the atmosphere via a needle. Then $ClCH_2CN$ (12 mmol, 200 µL) and MeCN (1.5 mL) were added with a syringe under N_2 atmosphere, the vial was moved to an alloy plate and put into a Parr 4560 series autoclave (300 mL) under N_2 atmosphere. At room temperature, the autoclave flushed with N_2 three times then charged with 30 bar CO. The autoclave was placed on a

heating plate equipped with a magnetic stirrer. The reaction mixture was test by GC.

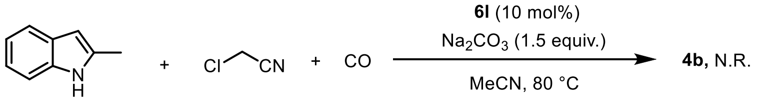

2c

A 4 mL screw-cap vial was charged with 2-methyl-1*H*-indole **2c** (0.3 mmol), **6l** (10 mol%), Na_2CO_3 (0.45 mmol, 47.7 mg) and an oven-dried stirring bar. The vial was closed with a Teflon septum and cap and connected to the atmosphere via a needle. Then $ClCH_2CN$ (12 mmol, 200 μL) and MeCN (1.5 mL) were added with a syringe under N_2 atmosphere, the vial was moved to an alloy plate and put into a Parr 4560 series autoclave (300 mL) under N_2 atmosphere. At room temperature, the autoclave flushed with N_2 three times then charged with 30 bar CO. The autoclave was placed on a heating plate equipped with a magnetic stirrer. The reaction mixture was test by GC.

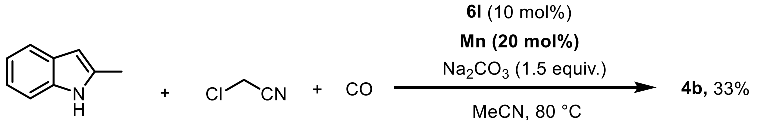

2c

A 4 mL screw-cap vial was charged with 2-methyl-1*H*-indole **2c** (0.3 mmol), **6l** (10 mol%), Mn (3.3 mg, 20 mol%), Na_2CO_3 (0.45 mmol, 47.7 mg) and an oven-dried stirring bar. The vial was closed with a Teflon septum and cap and connected to the atmosphere via a needle. Then $ClCH_2CN$ (12 mmol, 200 μL) and MeCN (1.5 mL) were added with a syringe under N_2 atmosphere, the vial was moved to an alloy plate and put into a Parr 4560 series autoclave (300 mL) under N_2 atmosphere. At room temperature, the autoclave flushed with N_2 three times then charged with 30 bar CO. The autoclave was placed on a heating plate equipped with a magnetic stirrer. The reaction mixture was test by GC.

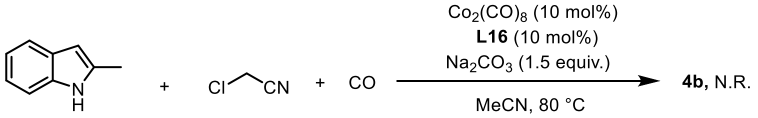

2c

A 4 mL screw-cap vial was charged with 2-methyl-1*H*-indole **2c** (0.3 mmol), $Co_2(CO)_8$ (5 mol%), **L16** (14.3 mg, 10 mol%), Na_2CO_3 (0.45 mmol, 47.7 mg) and an oven-dried stirring bar. The vial was closed with a Teflon septum and cap and connected to the atmosphere via a needle. Then $ClCH_2CN$ (12 mmol, 200 μL) and MeCN (1.5 mL) were added with a syringe under N_2 atmosphere, the vial was moved to an alloy plate and put into a Parr 4560 series autoclave (300 mL) under N_2 atmosphere. At room temperature, the autoclave flushed with N_2 three times then charged with 30

bar CO. The autoclave was placed on a heating plate equipped with a magnetic stirrer. The reaction mixture was test by GC.

12. Characterization data of products

3-(1-Methyl-1*H*-indol-3-yl)-3-oxopropanenitrile (3a)

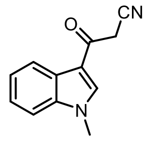

The title compound was prepared following the general procedure, purification by column chromatography on silica gel (petroleum ether/EtOAc = 1:1) yielded (54 mg, 91%) as a white solid. **^{1}H NMR (400 MHz, $CDCl_3$)** δ 8.34 – 8.28 (m, 1H), 7.80 (s, 1H), 7.40 – 7.33 (m, 3H), 3.88 (s, 3H), 3.86 (s, 2H). **^{13}C NMR (100 MHz, $CDCl_3$)** δ 180.4, 137.5, 136.0, 126.2, 124.2, 123.5, 122.4, 115.0, 114.3, 110.0, 33.9, 29.7. **HRMS (ESI-TOF) m/z:** $[M+H]^+$: Calcd. for $C_{12}H_{11}N_2O^+$: 190.0866. Found: 190.0867.

3-(1,2-Dimethyl-1*H*-indol-3-yl)-3-oxopropanenitrile (3b)

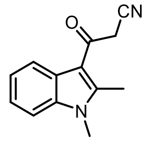

The title compound was prepared following the general procedure, purification by column chromatography on silica gel (petroleum ether/EtOAc = 1:1) yielded (52 mg, 82%) as a white solid. **^{1}H NMR (400 MHz, DMSO-*d*$_6$)** δ 8.03 – 7.90 (m, 1H), 7.64 – 7.52 (m, 1H), 7.32 – 7.18 (m, 2H), 4.58 (s, 2H), 3.76 (s, 3H), 2.73 (s, 3H). **^{13}C NMR (100 MHz, DMSO-*d*$_6$)** δ 184.0, 147.3, 136.9, 125.7, 122.7, 122.7, 120.8, 116.8, 111.7, 111.0, 33.6, 30.3, 13.0. **HRMS (ESI-TOF) m/z:** $[M+H]^+$: Calcd. for $C_{13}H_{13}N_2O^+$: 213.1023. Found: 213.1029.

3-(5-Bromo-1-methyl-1H-indol-3-yl)-3-oxopropanenitrile (3d)

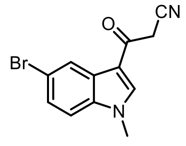

The title compound was prepared following the general procedure, purification by column chromatography on silica gel (petroleum ether/acetone = 2:1) yielded (55 mg, 66%) as a light-yellow solid. **^{1}H NMR (400 MHz, $CDCl_3$)** δ 8.42 (s, 1H), 8.25 (d, J = 2.0 Hz, 1H), 7.57 (d, J = 8.8 Hz, 1H), 7.45 (dd, J = 8.8, 2.0 Hz, 1H), 4.47 (s, 2H), 3.88 (s, 3H). **^{13}C NMR (100 MHz, $CDCl_3$)** δ 183.0, 140.2, 136.6, 127.6, 126.4, 123.7, 116.6, 116.1, 113.7, 113.1, 34.1, 30.0. **HRMS (ESI-TOF) m/z:** Calcd. for $C_{12}H_{10}BrN_2O^+$: 276.9972. Found: 276.9982.

3-(2-Cyanoacetyl)-1-methyl-1*H*-indole-5-carbonitrile (3e)

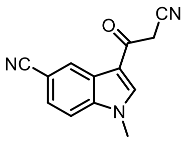

The title compound was prepared following the general procedure, purification by column chromatography on silica gel (petroleum ether/acetone = 2:1) yielded (48 mg, 72%) as a light-yellow solid. **^{1}H NMR (400 MHz, DMSO-*d*$_6$)** δ 8.58 (s, 1H), 8.48 (s, 1H), 7.82 (d, *J* = 8.4 Hz, 1H), 7.72 (dd, *J* = 8.4, 1.6 Hz, 1H), 4.53 (s, 2H), 3.94 (s, 3H). **^{13}C NMR (100 MHz, DMSO-*d*$_6$)** δ 183.3, 141.4, 139.5, 126.7, 126.4, 125.7, 120.3, 116.4, 113.9, 113.2, 105.4, 34.2, 30.2. **HRMS (ESI-TOF) m/z:** $[M+H]^+$: Calcd. for $C_{13}H_{10}N_3O^+$: 224.0819. Found: 224.0822.

3-(1,5-Dimethyl-1*H*-indol-3-yl)-3-oxopropanenitrile (3f)

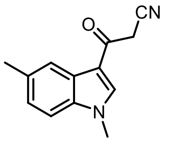

The title compound was prepared following the general procedure, purification by column chromatography on silica gel (petroleum ether/ EtOAc = 1:1) yielded (54 mg, 85%) as a white solid. **^{1}H NMR (400 MHz, DMSO-*d*$_6$)** δ 8.32 (s, 1H), 7.99 – 7.87 (m, 1H), 7.46 (d, *J* = 8.0 Hz, 1H), 7.15 (dd, *J* = 8.4, 2.0 Hz, 1H), 4.43 (s, 2H), 3.85 (s, 3H), 2.43 (s, 3H). **^{13}C NMR (100 MHz, DMSO-*d*$_6$)** δ 182.6, 139.2, 136.2, 132.2, 126.3, 125.3, 121.4, 116.9, 113.3, 111.1, 33.9, 29.9, 21.7. **HRMS (ESI-TOF) m/z:** $[M+H]^+$: Calcd. for $C_{13}H_{13}N_2O^+$: 213.1023. Found: 213.1029.

3-(5-Methoxy-1-methyl-1H-indol-3-yl)-3-oxopropanenitrile (3g)

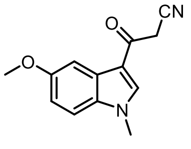

The title compound was prepared following the general procedure, purification by column chromatography on silica gel (petroleum ether/ acetone = 2:1) yielded (43 mg, 67%) as a light-yellow solid. **^{1}H NMR (700 MHz, DMSO-*d*$_6$)** δ 8.31 (s, 1H), 7.64 (d, *J* = 2.1 Hz, 1H), 7.49 (d, *J* = 9.1 Hz, 1H), 6.95 (dd, *J* = 9.1, 2.1 Hz, 1H), 4.42 (s, 2H), 3.85 (s, 3H), 3.80 (s, 3H). **^{13}C NMR (175 MHz, DMSO-*d*$_6$)** δ 182.6, 156.6, 139.2, 132.8, 126.9, 116.8, 113.5, 113.4, 112.4, 103.4, 55.8, 34.1, 29.8. **HRMS (ESI-TOF) m/z:** $[M+H]^+$: Calcd. for $C_{13}H_{13}N_2O_2^+$: 229.0972. Found: 229.0975.

3-(1,6-Dimethyl-1*H*-indol-3-yl)-3-oxopropanenitrile (3h)

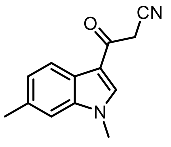

The title compound was prepared following the general procedure, purification by column chromatography on silica gel (petroleum ether/ EtOAc = 1:1) yielded (52 mg, 81%) as a white solid. **^{1}H NMR (400 MHz, DMSO-*d*$_6$)** δ 8.30 (s, 1H), 8.01 (d, *J* = 8.4 Hz, 1H), 7.38 (s, 1H), 7.11 (dd, *J* = 8.2, 1.6 Hz, 1H), 4.42 (s, 2H), 3.84 (s, 3H), 2.45 (s, 3H). **^{13}C NMR (100 MHz, DMSO-*d*$_6$)** δ 182.6, 138.8, 138.2, 133.3, 124.8, 123.9, 121.3, 116.9,

113.7, 111.2, 33.8, 29.8, 21.8. **HRMS (ESI-TOF) m/z:** [M+H]$^+$: Calcd. for $C_{13}H_{13}N_2O^+$: 213.1023. Found: 213.1025.

3-(1-Butyl-1*H*-indol-3-yl)-3-oxopropanenitrile (3j)

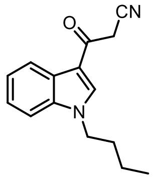

The title compound was prepared following the general procedure, purification by column chromatography on silica gel (petroleum ether/ EtOAc = 1:1) yielded (59 mg, 83%) as a light-yellow solid. **^{1}H NMR (400 MHz, CDCl$_3$)** δ 8.37 – 8.25 (m, 1H), 7.83 (d, *J* = 1.6 Hz, 1H), 7.51 – 7.30 (m, 3H), 4.18 (t, *J* = 7.2 Hz, 1H), 3.89 (s, 2H), 2.05 – 1.78 (m, 2H), 1.47 – 1.31 (m, 2H), 0.97 (t, *J* = 7.2 Hz, 3H). **^{13}C NMR (100 MHz, CDCl$_3$)** δ 180.6, 136.9, 135.3, 126.3, 124.1, 123.4, 122.5, 115.0, 114.2, 110.3, 47.3, 31.8, 29.7, 20.1, 13.6. **HRMS (ESI-TOF) m/z:** [M+H]$^+$: Calcd. for $C_{15}H_{17}N_2O^+$: 241.1336. Found: 241.1361.

3-(1-Benzyl-1*H*-indol-3-yl)-3-oxopropanenitrile (3k)

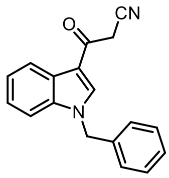

The title compound was prepared following the general procedure, purification by column chromatography on silica gel (petroleum ether/ EtOAc = 1:1) yielded (71 mg, 86%) as a white solid. **^{1}H NMR (400 MHz, CDCl$_3$)** δ 8.39 – 8.29 (m, 1H), 7.83 (s, 1H), 7.46 – 7.31 (m, 6H), 7.21 – 7.10 (m, 2H), 5.38 (s, 2H), 3.88 (s, 2H). **^{13}C NMR (100 MHz, CDCl$_3$)** δ 181.3, 137.2, 135.9, 135.1, 129.2, 128.5, 127.2, 126.3, 124.4, 123.7, 122.4, 114.7, 110.7, 51.1, 29.7. **HRMS (ESI-TOF) m/z:** [M+H]$^+$: Calcd. for $C_{18}H_{15}N_2O^+$: 275.1179. Found: 275.1180.

3-Oxo-3-(1-(4-vinylbenzyl)-1*H*-indol-3-yl)propanenitrile (3l)

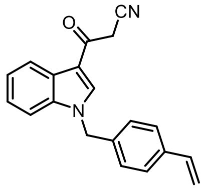

The title compound was prepared following the general procedure, purification by column chromatography on silica gel (petroleum ether/ EtOAc = 1:1) yielded (72 mg, 80%) as a white solid. **^{1}H NMR (400 MHz, DMSO-*d*$_6$)** δ 8.59 (s, 1H), 8.29 – 8.07 (m, 1H), 7.64 – 7.54 (m, 1H), 7.44 (d, *J* = 8.0 Hz, 2H), 7.33 – 7.23 (m, 4H), 6.69 (dd, *J* = 17.6, 11.2 Hz, 1H), 5.80 (d, *J* = 17.6 Hz, 1H), 5.52 (s, 2H), 5.24 (d, *J* = 11.2 Hz, 1H), 4.52 (s, 2H). **^{13}C NMR (100 MHz, DMSO-*d*$_6$)** δ 183.2, 138.8, 137.2, 137.0, 136.7, 136.5, 128.1, 126.9, 126.3, 124.0, 123.3, 121.8, 116.8, 115.1, 114.3, 111.9, 50.2, 30.1. **HRMS (ESI-TOF) m/z:** [M+H]$^+$: Calcd. for $C_{20}H_{17}N_2O^+$: 301.1336. Found: 301.1338.

3-(1-Cinnamyl-1*H*-indol-3-yl)-3-oxopropanenitrile (3m)

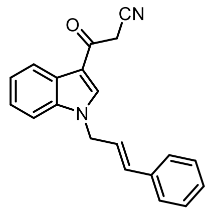

The title compound was prepared following the general procedure, purification by column chromatography on silica gel (petroleum ether/ EtOAc = 1:1) yielded (74 mg, 82%) as a white solid. **^{1}H NMR (400 MHz, CDCl$_3$)** δ 8.38 – 8.28 (m, 1H), 7.87 (s, 1H), 7.50 – 7.42 (m, 1H), 7.38 – 7.26 (m, 7H), 6.66 – 6.56 (m, 1H), 6.34 (dt, *J* = 16.0, 6.0 Hz, 1H), 4.94 (dd, *J* = 6.2, 1.6 Hz, 2H), 3.89 (s, 2H). **^{13}C NMR (100 MHz, CDCl$_3$)** δ 181.1, 137.0, 135.5, 135.4, 134.6, 128.8, 128.5, 126.7, 126.3, 124.4, 123.7, 122.3, 114.6, 110.6, 49.4, 29.7. **HRMS (ESI-TOF) m/z:** [M+H]$^+$: Calcd. for $C_{20}H_{17}N_2O^+$: 301.1336. Found: 301.1339.

3-Oxo-3-(1-phenyl-1*H*-indol-3-yl)propanenitrile (3n)

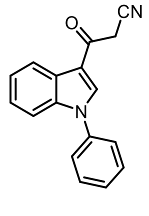

The title compound was prepared following the general procedure, purification by column chromatography on silica gel (petroleum ether/acetone = 10:1) yielded (58 mg, 74%) as a white solid. **^{1}H NMR (400 MHz, CDCl$_3$)** δ 8.50 – 8.35 (m, 1H), 8.01 (s, 1H), 7.69 – 7.56 (m, 2H), 7.54 – 7.44 (m, 4H), 7.43 – 7.31 (m, 2H), 3.95 (s, 2H). **^{13}C NMR (100 MHz, CDCl$_3$)** δ 181.0, 137.8, 137.2, 134.9, 130.1, 128.7, 126.3, 125.1, 124.8, 124.0, 122.6, 116.0, 114.7, 111.2, 29.9. **HRMS (ESI-TOF) m/z:** [M+H]$^+$: Calcd. for $C_{17}H_{13}N_2O^+$: 261.1023. Found: 261.1036.

3-Oxo-3-(1-phenyl-1*H*-pyrrol-2-yl)propanenitrile (3o)

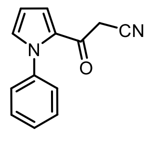

The title compound was prepared following the general procedure, purification by column chromatography on silica gel (petroleum ether/ EtOAc = 5:1) yielded (44 mg, 71%) as a yellow oil. **^{1}H NMR (700 MHz, CDCl$_3$)** δ 7.48 – 7.40 (m, 3H), 7.33 – 7.24 (m, 2H), 7.13 (dd, *J* = 4.2, 1.4 Hz, 1H), 7.07 (dd, *J* = 2.8, 1.4 Hz, 1H), 6.37 (dd, *J* = 4.2, 2.8 Hz, 1H), 3.87 (s, 2H). **^{13}C NMR (175 MHz, CDCl$_3$)** δ 175.3, 139.9, 133.3, 128.9, 128.6, 128.4, 126.2, 121.7, 114.2, 110.3, 29.5. **HRMS (ESI-TOF) m/z:** [M+H]$^+$: Calcd. for $C_{13}H_{11}N_2O^+$: 211.0866. Found: 211.0869.

3-(1*H*-Indol-3-yl)-3-oxopropanenitrile (4a)

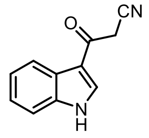

The title compound was prepared following the general procedure, purification by column chromatography on silica gel (petroleum ether/acetone = 2:1) yielded (43 mg, 78%) as a light-yellow solid. **^{1}H NMR (400 MHz, DMSO-*d*$_6$)** δ 12.20 (s, NH, 1H), 8.40 (d, *J* = 3.2 Hz, 1H), 8.18 (dt, *J* = 5.6, 2.4 Hz, 1H), 7.54 (dt, *J* = 7.2, 1.6 Hz, 1H), 7.38 – 7.04

(m, 2H), 4.52 (s, 2H). **^{13}C NMR (100 MHz, DMSO-*d*$_6$)** δ 183.4, 137.1, 136.0, 125.7, 123.8, 122.8, 121.5, 116.9, 114.9, 112.9, 29.9. **HRMS (ESI-TOF) m/z:** [M+H]$^+$: Calcd. for $C_{11}H_9N_2O^+$: 185.0710. Found: 185.0708.

3-(2-Methyl-1H-indol-3-yl)-3-oxopropanenitrile (4b)

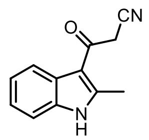

The title compound was prepared following the general procedure, purification by column chromatography on silica gel (petroleum ether/acetone = 2:1) yielded (54 mg, 92%) as a light-yellow solid. **^{1}H NMR (400 MHz, DMSO-*d*$_6$)** δ 12.08 (s, NH, 1H), 7.98 (dd, *J* = 6.0, 3.2 Hz, 1H), 7.59 – 7.31 (m, 1H), 7.18 (dd, *J* = 6.0, 3.2 Hz, 2H), 4.63 – 4.31 (m, 2H), 2.69 (s, 3H). **^{13}C NMR (100 MHz, DMSO-*d*$_6$)** δ 183.8, 183.8, 146.3, 135.2, 126.9, 122.8, 122.3, 120.9, 116.8, 111.9, 33.1, 15.4. **HRMS (ESI-TOF) m/z:** [M+H]$^+$: Calcd. for $C_{12}H_{11}N_2O^+$: 199.0866. Found: 199.0864.

3-(4-Methyl-1H-indol-3-yl)-3-oxopropanenitrile (4c)

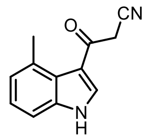

The title compound was prepared following the general procedure, purification by column chromatography on silica gel (petroleum ether/acetone = 2:1) yielded (45 mg, 76%) as a light-yellow solid. **^{1}H NMR (400 MHz, DMSO-*d*$_6$)** δ 12.16 (s, NH, 1H), 8.36 (d, *J* = 3.6 Hz, 1H), 7.31 – 7.27 (m, 1H), 7.13 (t, *J* = 8.0 Hz, 1H), 6.96 (dt, *J* = 7.2, 0.8 Hz, 1H), 4.52 (s, 2H), 2.72 (s, 3H). **^{13}C NMR (100 MHz, DMSO-*d*$_6$)** δ 182.8, 138.2, 136.9, 132.1, 124.5, 124.3, 124.0, 117.2, 116.4, 110.4, 30.9, 23.2. **HRMS (ESI-TOF) m/z:** [M+H]$^+$: Calcd. for $C_{12}H_{11}N_2O^+$: 199.0866. Found: 199.0867.

3-(4-Methoxy-1*H*-indol-3-yl)-3-oxopropanenitrile (4d)

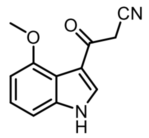

The title compound was prepared following the general procedure, purification by column chromatography on silica gel (petroleum ether/acetone = 2:1) yielded (45 mg, 70%) as a light-yellow solid. **^{1}H NMR (400 MHz, DMSO-*d*$_6$)** δ 12.17 (s, NH, 1H), 8.14 (d, *J* = 3.2 Hz, 1H), 7.28 – 7.08 (m, 2H), 6.72 (d, *J* = 7.6 Hz, 1H), 4.53 (s, 2H), 3.89 (s, 3H). **^{13}C NMR (100 MHz, DMSO-*d*$_6$)** δ 183.7, 153.7, 139.2, 134.2, 124.6, 117.1, 116.2, 114.4, 106.1, 103.4, 55.8, 32.3. **HRMS (ESI-TOF) m/z:** [M+H]$^+$: Calcd. for $C_{12}H_{11}N_2O_2^+$: 215.0816. Found: 215.0815.

3-(5-Methyl-1*H*-indol-3-yl)-3-oxopropanenitrile (4f)

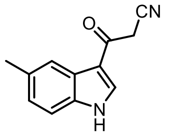

The title compound was prepared following the general procedure, purification by column chromatography on silica gel (petroleum ether/acetone = 2:1) yielded (43 mg, 72%) as a light-yellow solid. **^{1}H NMR (400 MHz, DMSO-*d*$_6$)** δ 12.08 (s, NH, 1H), 8.32 (d, *J* = 3.6 Hz, 1H), 7.96 (s, 1H), 7.40 (d, *J* = 8.0 Hz, 1H), 7.08 (dd, *J* = 8.0, 1.6 Hz, 1H), 4.47 (s, 2H), 2.42 (s, 3H). **^{13}C NMR (100 MHz, DMSO-*d*$_6$)** δ 183.2, 135.9, 135.4, 131.7, 125.9, 125.2, 121.2, 117.0, 114.6, 112.5, 29.8, 21.8. **HRMS (ESI-TOF) m/z:** [M+H]$^+$: Calcd. for $C_{12}H_{11}N_2O^+$: 199.0866. Found: 199.0864.

3-(5-Bromo-1*H*-indol-3-yl)-3-oxopropanenitrile (4g)

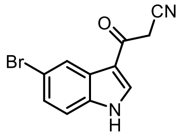

The title compound was prepared following the general procedure, purification by column chromatography on silica gel (petroleum ether/acetone = 2:1) yielded (55 mg, 60%) as a white solid. **^{1}H NMR (400 MHz, DMSO-*d*$_6$)** δ 12.36 (s, NH, 1H), 8.43 (d, *J* = 3.2 Hz, 1H), 8.27 (d, *J* = 2.0 Hz, 1H), 7.50 (dd, *J* = 8.8, 0.4 Hz, 1H), 7.40 (dd, *J* = 8.8, 2.0 Hz, 1H), 4.52 (s, 2H). **^{13}C NMR (100 MHz, DMSO-*d*$_6$)** δ 183.6, 137.0, 135.9, 127.4, 126.4, 123.6, 116.7, 115.6, 115.0, 114.4, 30.0. **HRMS (ESI-TOF) m/z:** [M+H]$^+$: Calcd. for $C_{11}H_8ClN_2O^+$: 219.0320. Found: 219.0321.

3-(6-Methyl-1*H*-indol-3-yl)-3-oxopropanenitrile (4h)

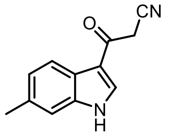

The title compound was prepared following the general procedure, purification by column chromatography on silica gel (petroleum ether/acetone = 2:1) yielded (42 mg, 71%) as a light-yellow solid. **^{1}H NMR (400 MHz, DMSO-*d*$_6$)** δ 12.04 (s, NH, 1H), 8.55 – 8.25 (m, 1H), 8.00 (d, *J* = 8.0 Hz, 1H), 7.29 (s, 1H), 7.06 (d, *J* = 8.0 Hz, 1H), 4.47 (s, 1H), 2.41 (s, 3H). **^{13}C NMR (100 MHz, DMSO-*d*$_6$)** ^{13}C NMR (100 MHz, DMSO) δ 183.2, 137.6, 135.5, 133.1, 124.4, 123.5, 121.2, 116.9, 114.9, 112.6, 29.8, 21.7. **HRMS (ESI-TOF) m/z:** [M+H]$^+$: Calcd. for $C_{12}H_{11}N_2O^+$: 199.0866. Found: 199.0869.

3-(6-Chloro-1*H*-indol-3-yl)-3-oxopropanenitrile (4i)

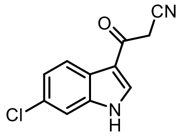

The title compound was prepared following the general procedure, purification by column chromatography on silica gel (petroleum ether/acetone = 2:1) yielded (42 mg, 64%) as a light-yellow solid. **^{1}H NMR (400 MHz, DMSO-*d*$_6$)** δ 12.27 (s, NH, 1H), 8.42 (d, *J* = 3.2 Hz, 1H), 8.12 (d, *J* = 8.4 Hz, 1H), 7.58 (d, *J* = 1.6 Hz, 1H), 7.27 (dd, *J* = 8.4, 2.0 Hz, 1H), 4.52 (s, 2H). **^{13}C NMR (100 MHz, DMSO-*d*$_6$)** δ 183.5, 137.6, 136.8, 128.3, 124.4,

123.1, 122.8, 116.7, 114.9, 112.7, 30.0. **HRMS (ESI-TOF) m/z:** $[M+H]^+$: Calcd. for $C_{11}H_8BrN_2O^+$: 262.9815. Found: 262.9810.

3-(7-Methyl-1*H*-indol-3-yl)-3-oxopropanenitrile (3u)

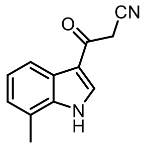

The title compound was prepared following the general procedure, purification by column chromatography on silica gel (petroleum ether/acetone = 2:1) yielded (40 mg, 68%) as a light-yellow solid. **^{1}H NMR (400 MHz, DMSO-d_6)** δ 12.19 (s, NH, 1H), 8.40 (d, J = 3.2 Hz, 1H), 8.01 (d, J = 7.6 Hz, 1H), 7.15 (t, J = 7.2 Hz, 1H), 7.05 (d, J = 7.2 Hz, 1H), 4.51 (s, 2H), 2.52 (s, 3H). **^{13}C NMR (100 MHz, DMSO-d_6)** δ 183.4, 136.7, 135.7, 125.5, 124.3, 123.0, 122.2, 119.1, 117.0, 115.3, 29.9, 17.1. **HRMS (ESI-TOF) m/z:** $[M+H]^+$: Calcd. for $C_{12}H_{11}N_2O^+$: 199.0866. Found: 199.0869.

1-(1-Methyl-1*H*-indol-3-yl)pentan-1-one (5a)

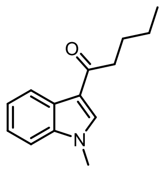

The title compound was prepared following the general procedure, purification by column chromatography on silica gel (petroleum ether/ EtOAc = 10:1) yielded (26 mg, 41%) as a colorless oil. **^{1}H NMR (700 MHz, CDCl$_3$)** δ 8.51 – 8.33 (m, 1H), 7.72 (s, 1H), 7.37 – 7.28 (m, 3H), 3.85 (s, 3H), 2.84 (t, J = 7.7 Hz, 2H), 1.80 – 1.73 (m, 2H), 1.47 – 1.39 (m, 2H), 0.96 (t, J = 7.7 Hz, 3H). **^{13}C NMR (175 MHz, CDCl$_3$)** δ 196.1, 137.5, 135.2, 126.4, 123.3, 122.7, 122.5, 116.7, 109.6, 39.7, 33.5, 27.4, 22.7, 14.0. **HRMS (ESI-TOF) m/z:** $[M+H]^+$: Calcd. for $C_{14}H_{18}NO^+$: 216.1383. Found: 216.1387.

1-(2-Methyl-1*H*-indol-3-yl)pentan-1-one (5c)

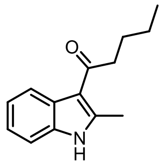

The title compound was prepared following the general procedure, purification by column chromatography on silica gel (petroleum ether/ EtOAc = 6:1) yielded (26 mg, 41%) as a white solid. **^{1}H NMR (700 MHz, CDCl$_3$)** δ 8.82 (s, NH, 1H), 7.99 (d, J = 7.7 Hz, 1H), 7.38 – 7.31 (m, 1H), 7.27 – 7.23 (m, 1H), 7.22 – 7.18 (m, 1H), 2.99 (t, J = 7.7 Hz, 2H), 2.75 (s, 3H), 1.87 – 1.75 (m, 2H), 1.56 – 1.43 (m, 2H), 0.97 (t, J = 7.7 Hz, 3H). **^{13}C NMR (175 MHz, CDCl$_3$)** δ 197.9, 143.8, 134.6, 126.7, 122.3, 122.0, 120.9, 114.3, 110.9, 42.8, 26.4, 22.7, 15.6, 14.1. **HRMS (ESI-TOF) m/z:** $[M+H]^+$: Calcd. for $C_{14}H_{18}NO^+$: 216.1383. Found: 216.1386.

2-Methyl-1-(2-methyl-1*H*-indol-3-yl)butan-1-one (5d)

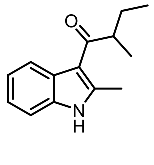

The title compound was prepared following the general procedure, purification by column chromatography on silica gel (petroleum ether/ EtOAc = 6:1) yielded (26 mg, 41%) as a colorless oil. **^{1}H NMR (700 MHz, CDCl$_3$)** δ 8.66 (s, NH, 1H), 7.95

(d, J = 8.0 Hz, 1H), 7.42 – 7.30 (m, 1H), 7.25 – 7.23 (m, 1H), 7.22 – 7.19 (m, 1H), 3.35 (h, J = 7.0 Hz, 1H), 2.76 (s, 3H), 2.01 – 1.88 (m, 1H), 1.62 – 1.45 (m, 1H), 1.26 (d, J = 7.0 Hz, 3H), 0.98 (t, J = 7.7 Hz, 3H). **^{13}C NMR (175 MHz, $CDCl_3$)** δ 201.8, 144.1, 134.6, 126.4, 122.2, 122.0, 120.7, 114.0, 110.9, 45.5, 26.3, 16.4, 15.6, 11.9. **HRMS (ESI-TOF) m/z:** $[M+H]^+$: Calcd. for $C_{14}H_{18}NO^+$: 216.1383. Found: 216.1386.

13. Reference

1. W.-B. Liu, D. P. Schuman and B. M. Stoltz, *J. Am. Chem. Soc.*, 2017, **139**, 6867−6879.

2. H. Wang, Z. Wang, Y.-L. Wang, R.-R. Zhou, G.-C. Wu, S.-Y. Yin, X. Yan and B. Wang, *Org. Lett.*, 2017, **19**, 6140−6143.

3. E. A. Juan, C. Cyrille, G. Soph and J. M. SaveÂant, *J. Am. Chem. Soc.*, 2005, **127**, 5049-5055.

4. J. Chen, L.-F. Wu, Z.-Y. Song, Y. Wang, Z.-K. Li, Y. Wang and S.-L. Zhu, *J. Am. Chem. Soc.*, 2024, **146**, 26223−26232.

14. Spectra of compounds

[1]**H NMR** spectrum of **S4** ($CDCl_3$)

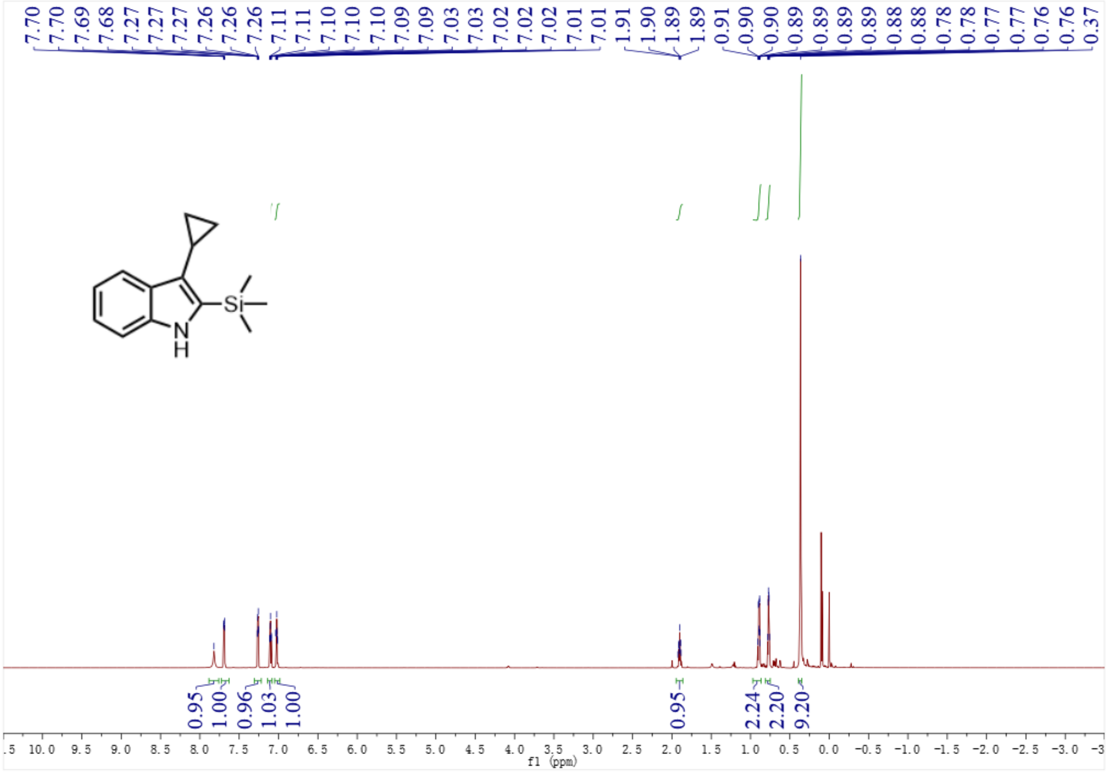

[13]**C NMR** spectrum of **S4** ($CDCl_3$)

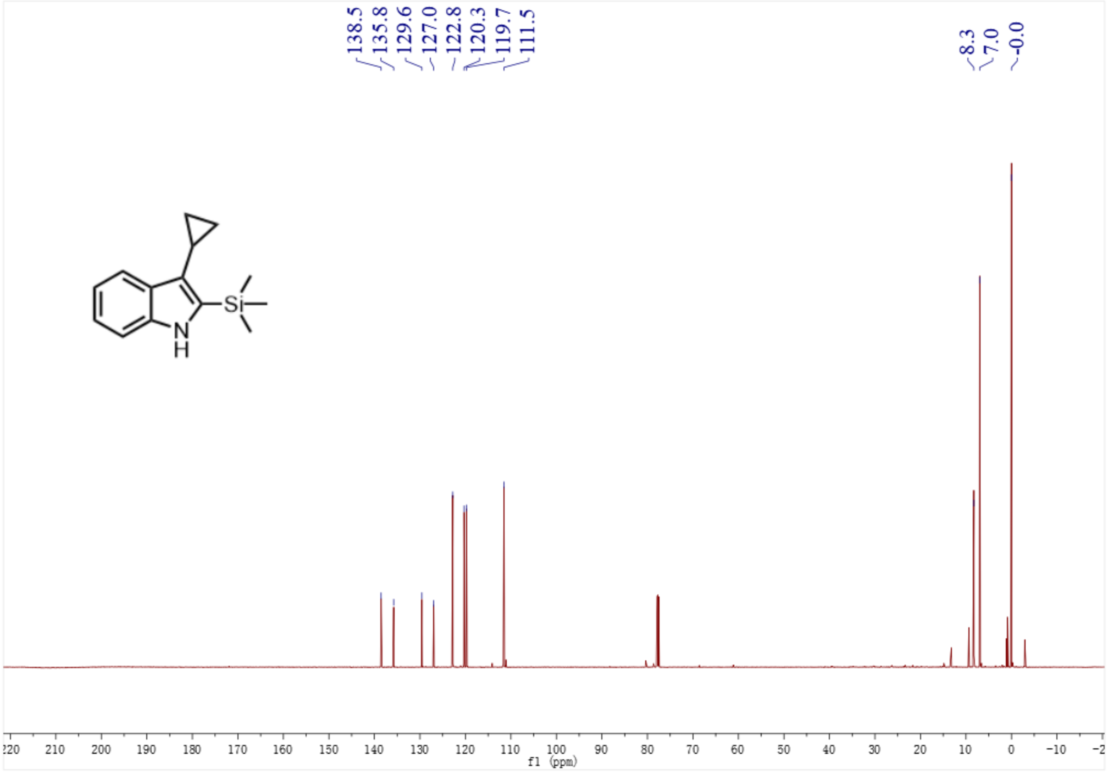

[13]H NMR spectrum of **S5** ($CDCl_3$)

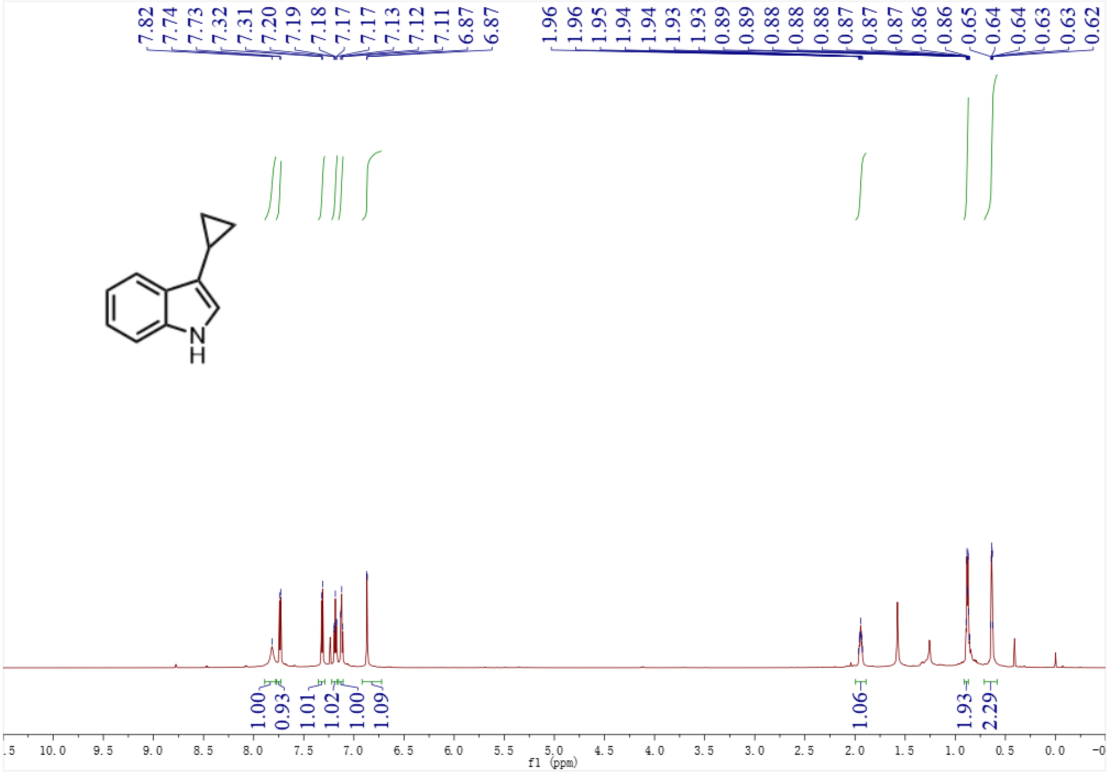

^{13}C NMR spectrum of **S5** ($CDCl_3$)

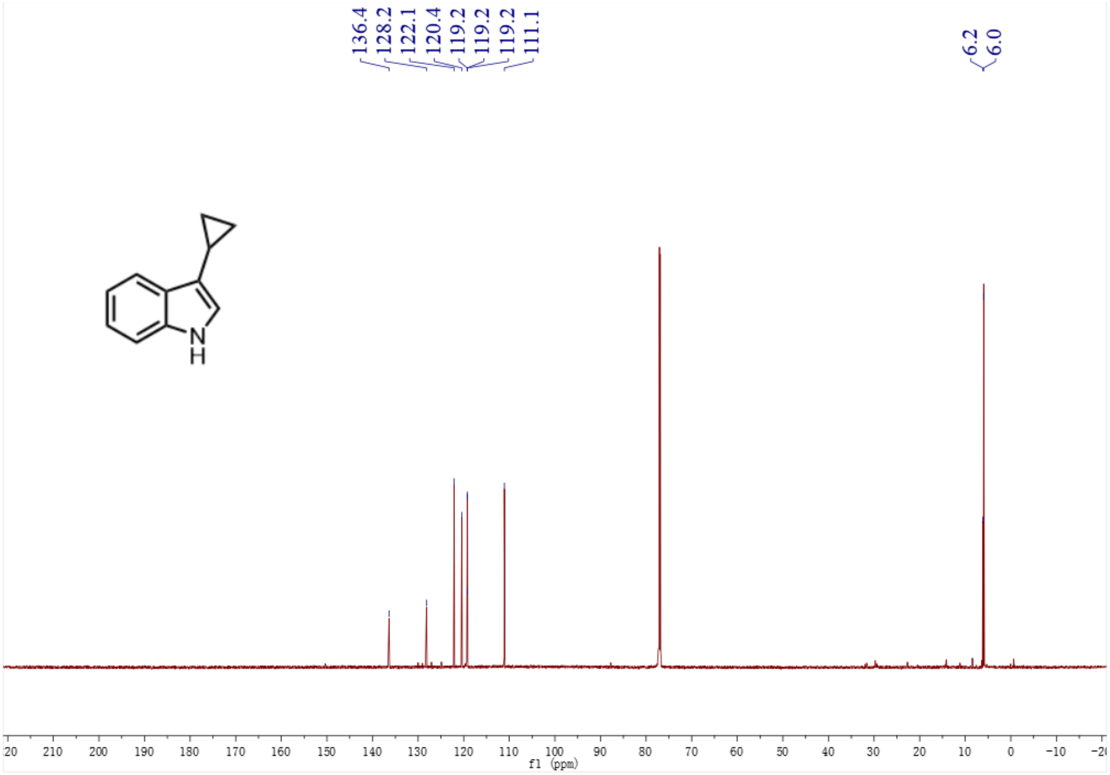

^{1}H NMR spectrum of **3a** ($CDCl_3$)

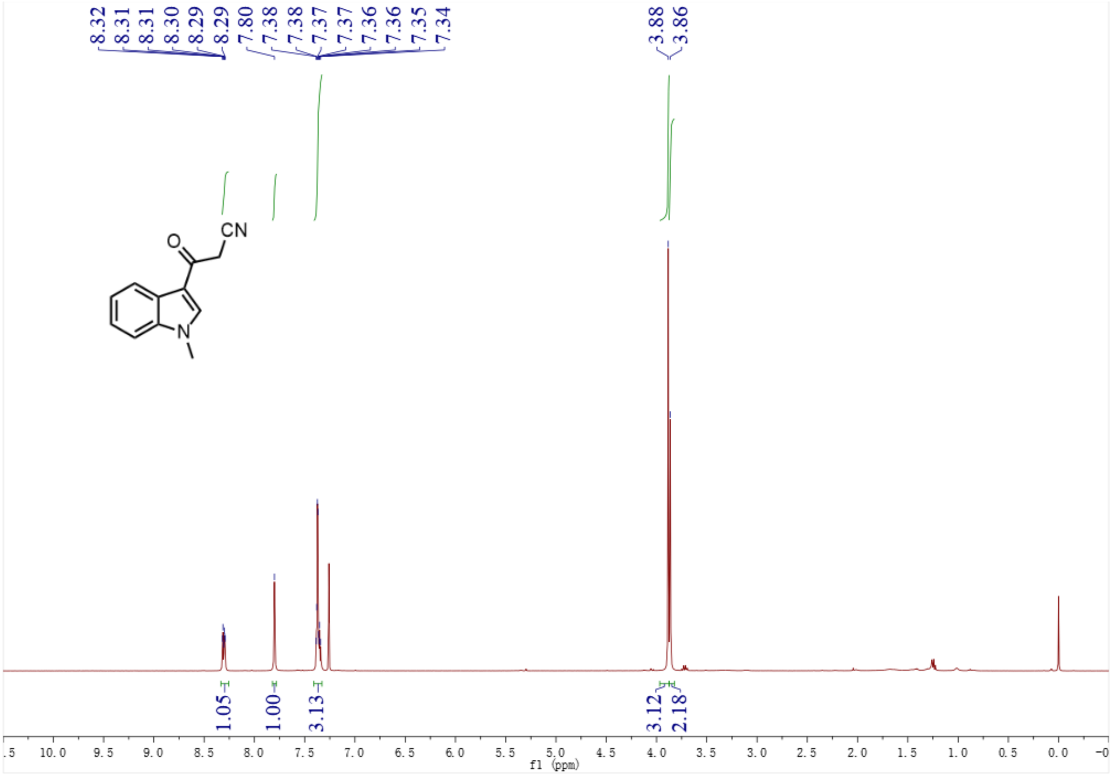

^{13}C NMR spectrum of **3a** ($CDCl_3$)

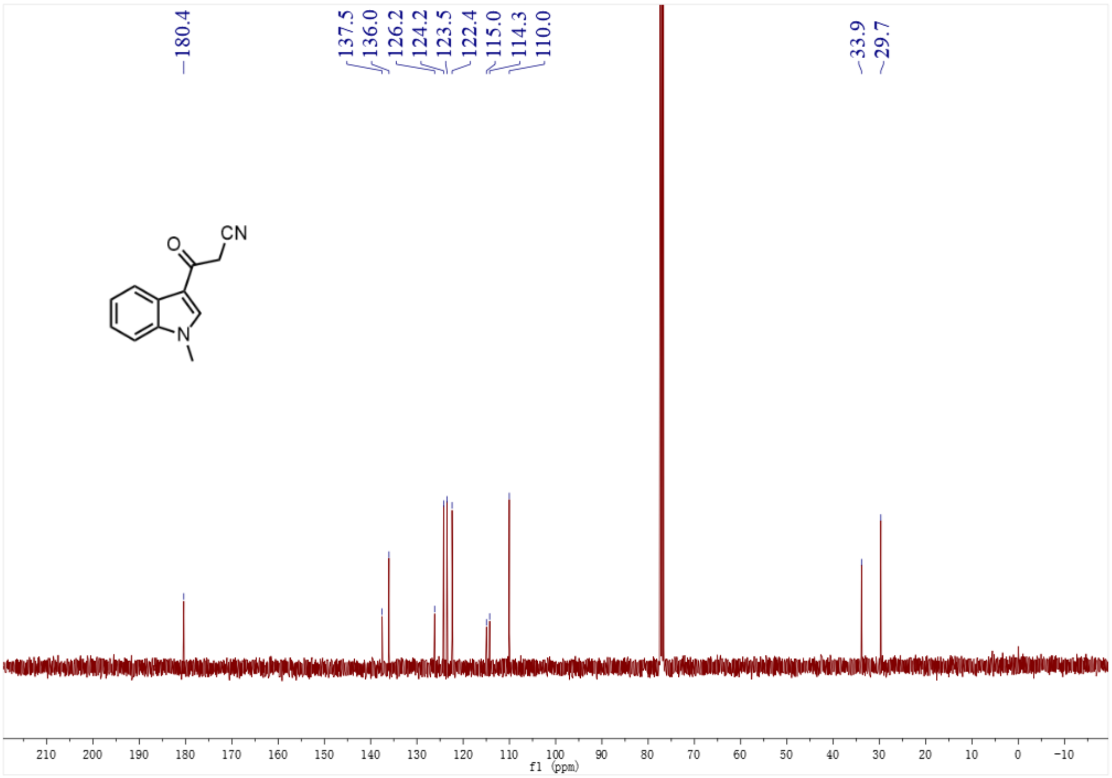

^{1}**H NMR** spectrum of **3b** (DMSO-*d*$_6$)

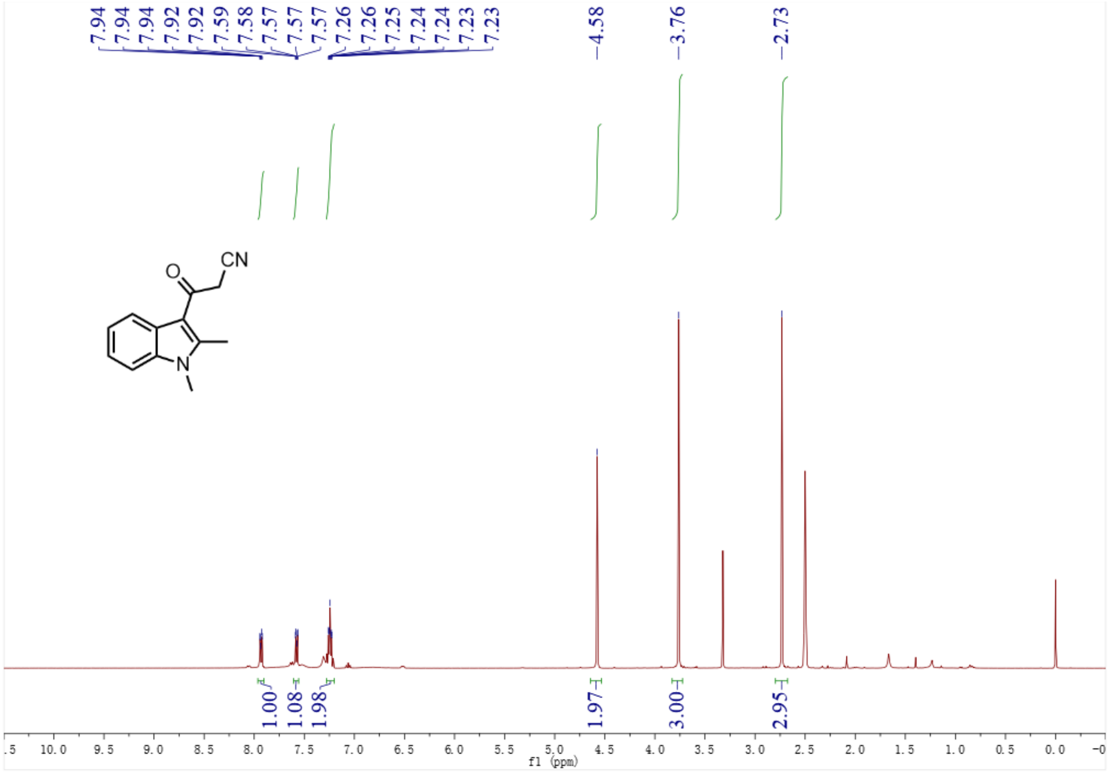

^{13}**C NMR** spectrum of **3b** (DMSO-*d*$_6$)

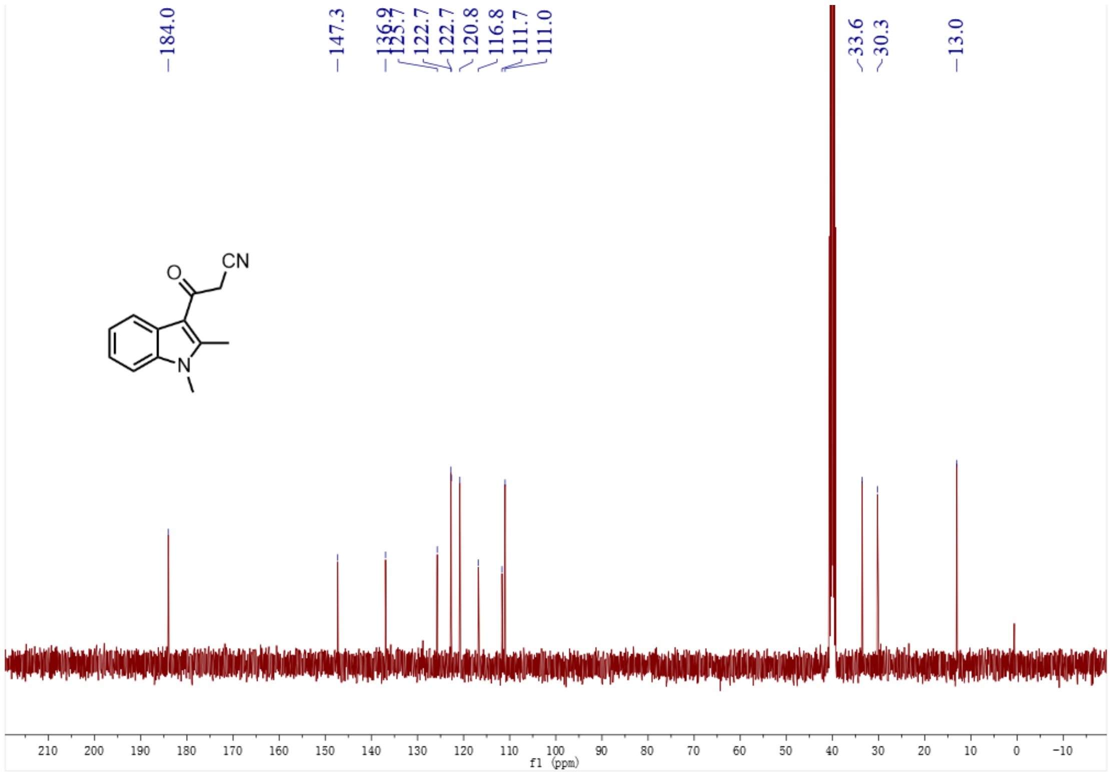

^{1}**H NMR** spectrum of **3d** (DMSO-d_6)

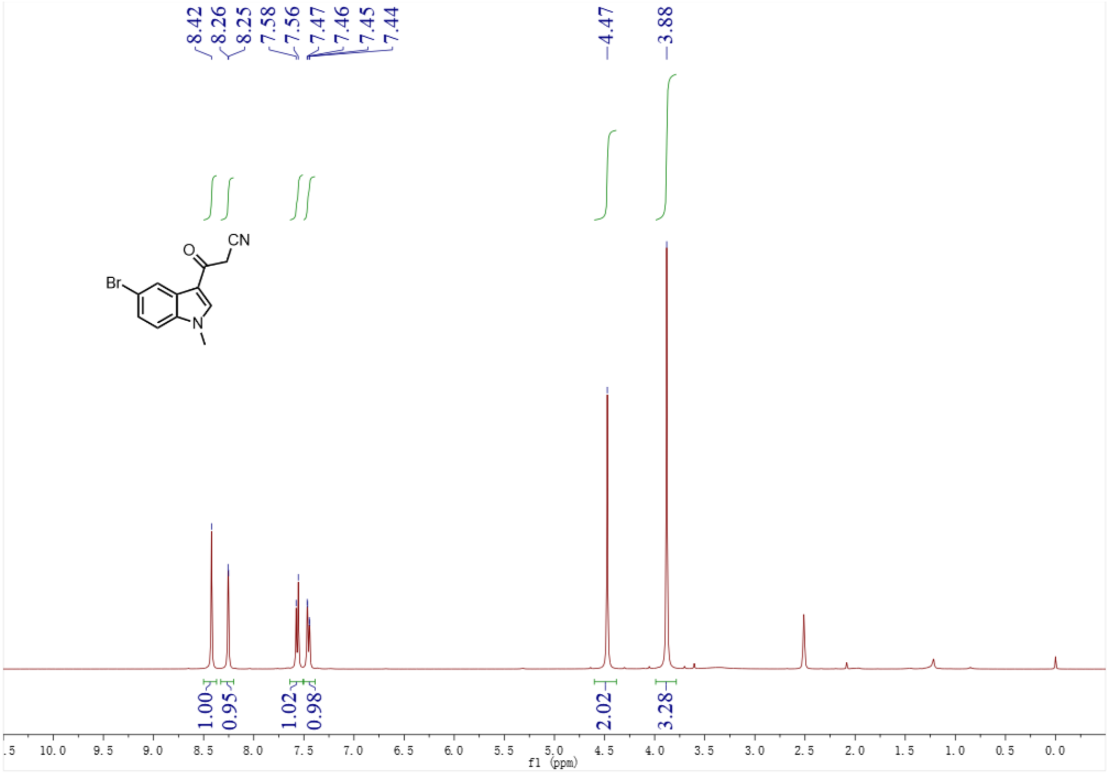

^{13}**C NMR** spectrum of **3d** (DMSO-d_6)

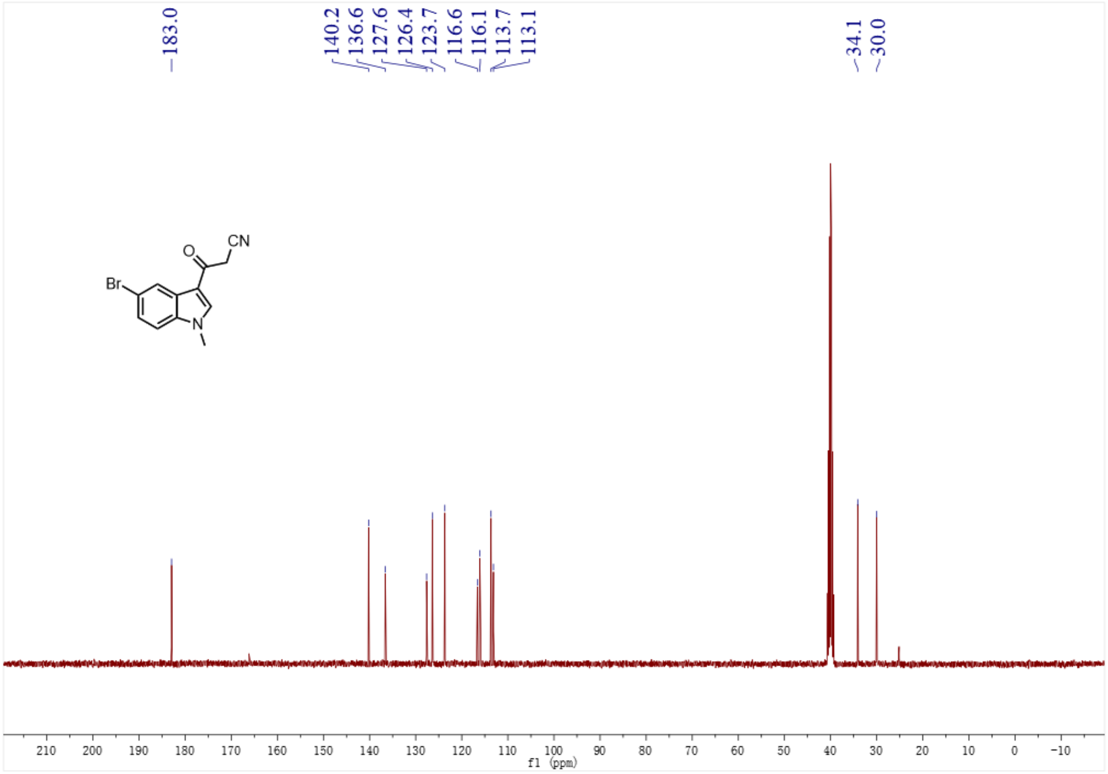

^{1}H NMR spectrum of 3e (DMSO-d_6)

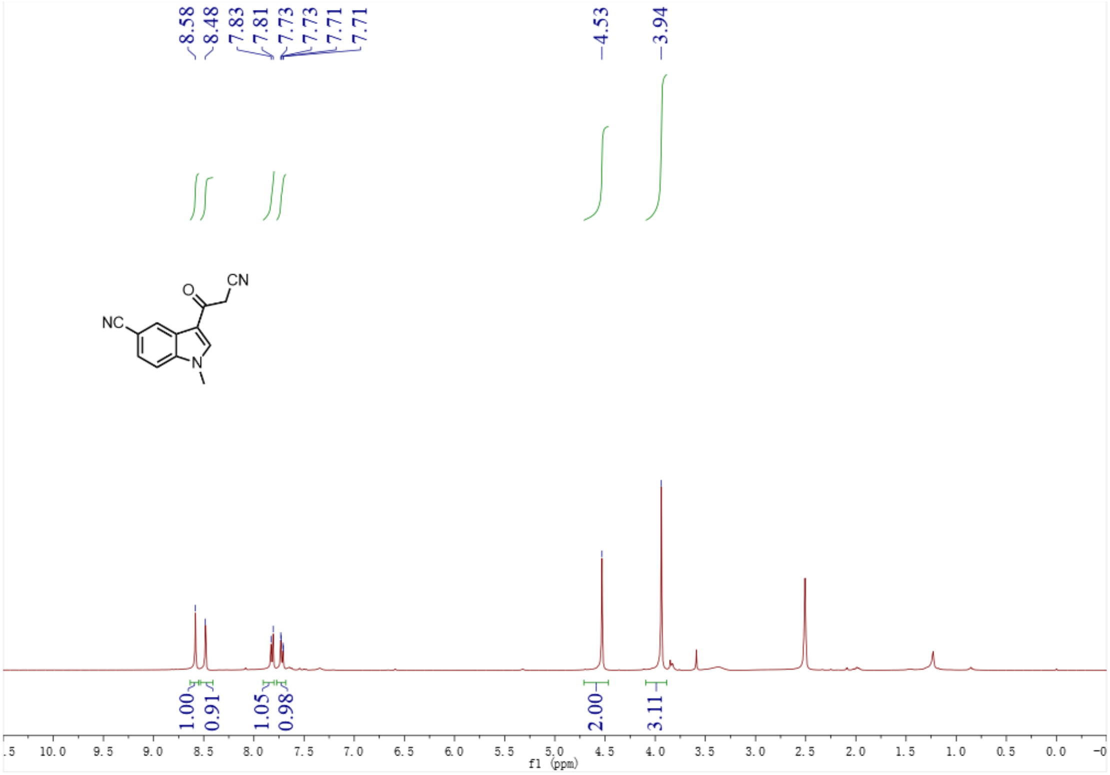

^{13}C NMR spectrum of 3e (DMSO-d_6)

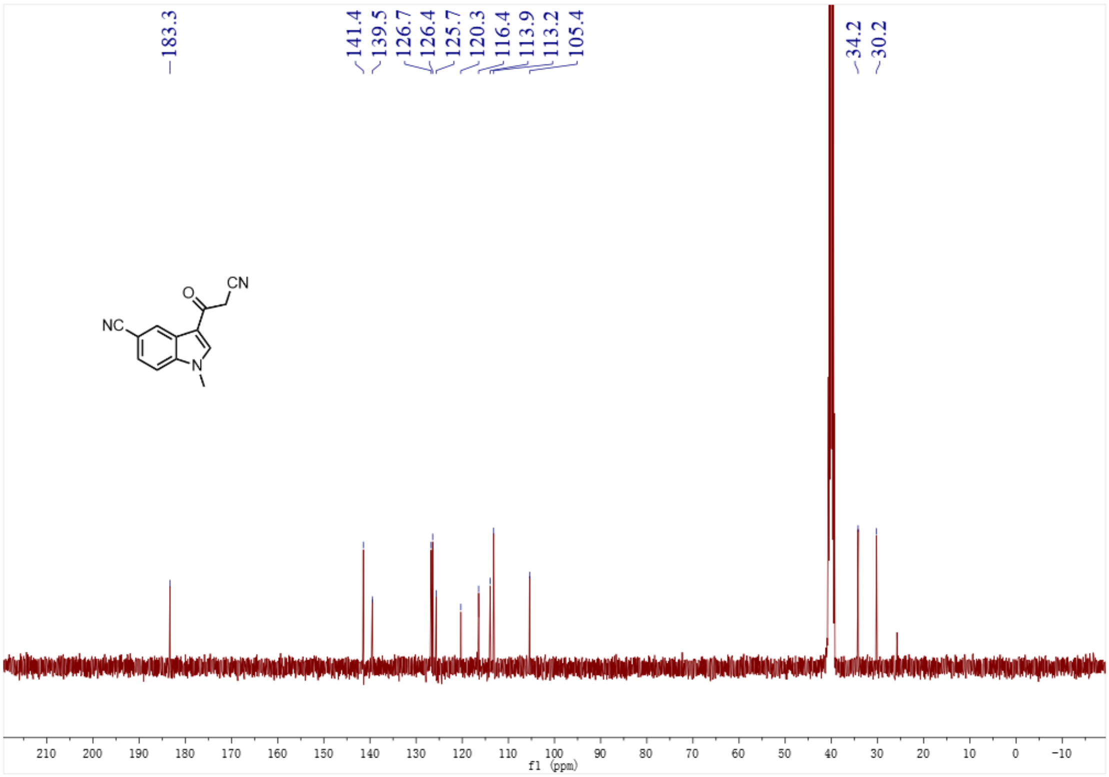

^{1}H NMR spectrum of **3f** (DMSO-d_6)

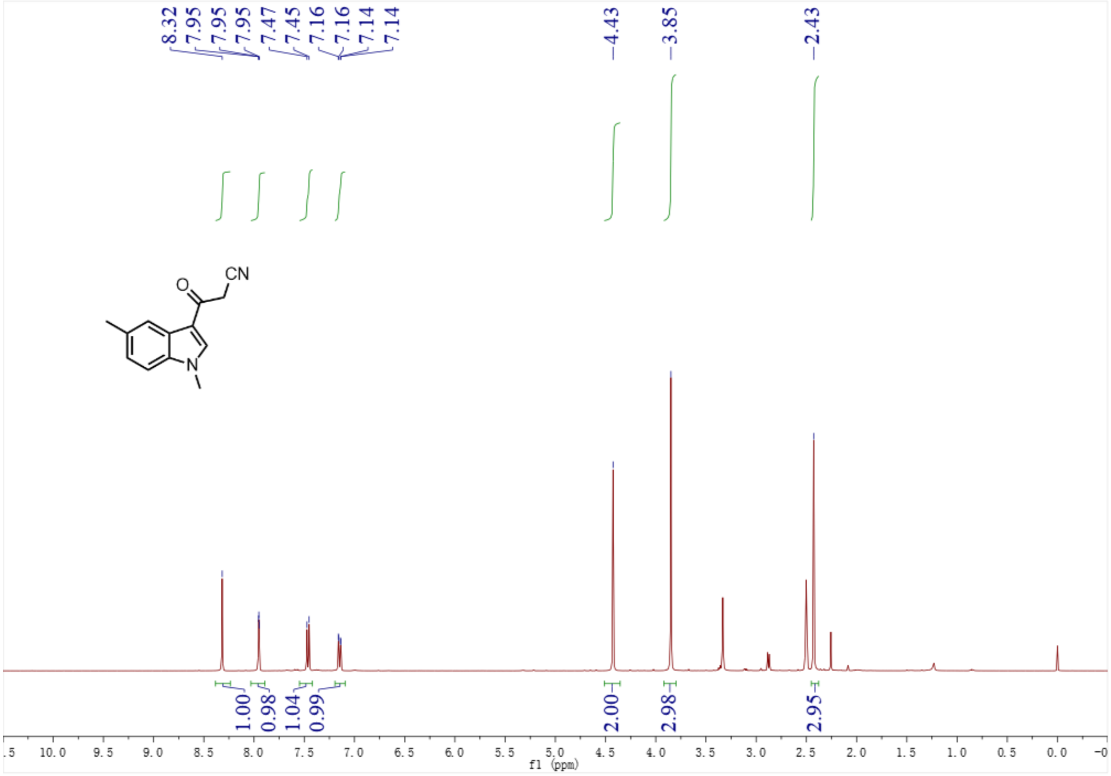

^{13}C NMR spectrum of **3f** (DMSO-d_6)

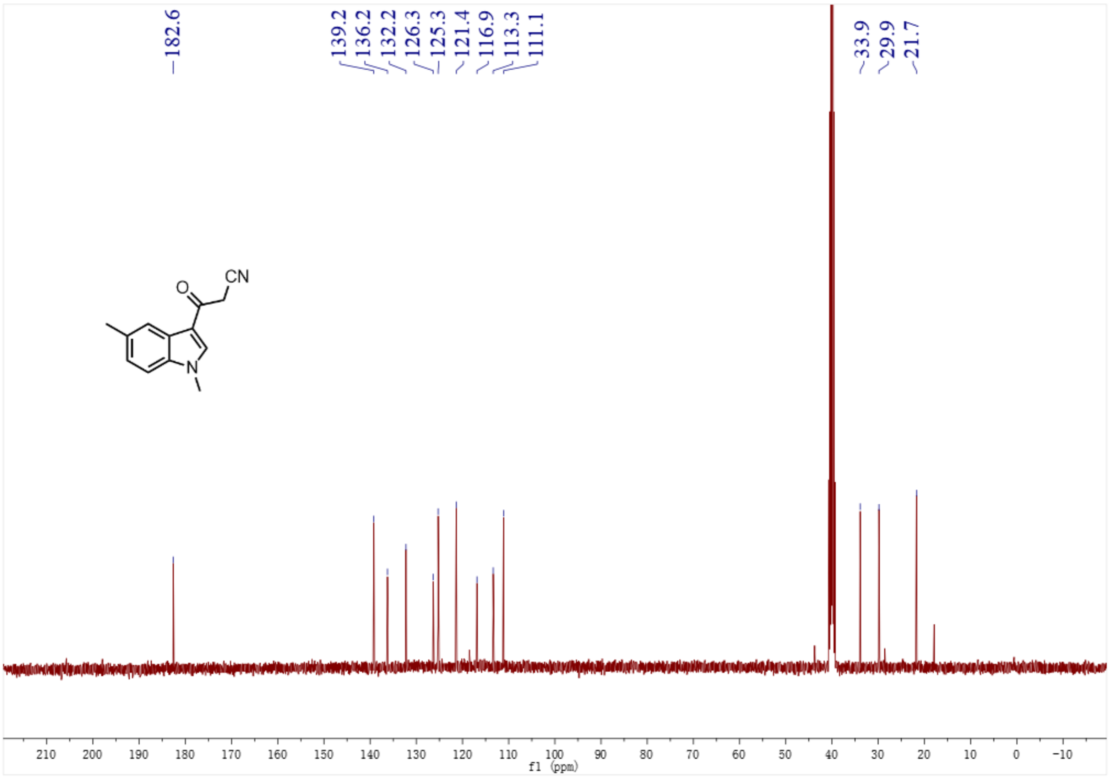

^{1}H NMR spectrum of **3g** (DMSO-d_6)

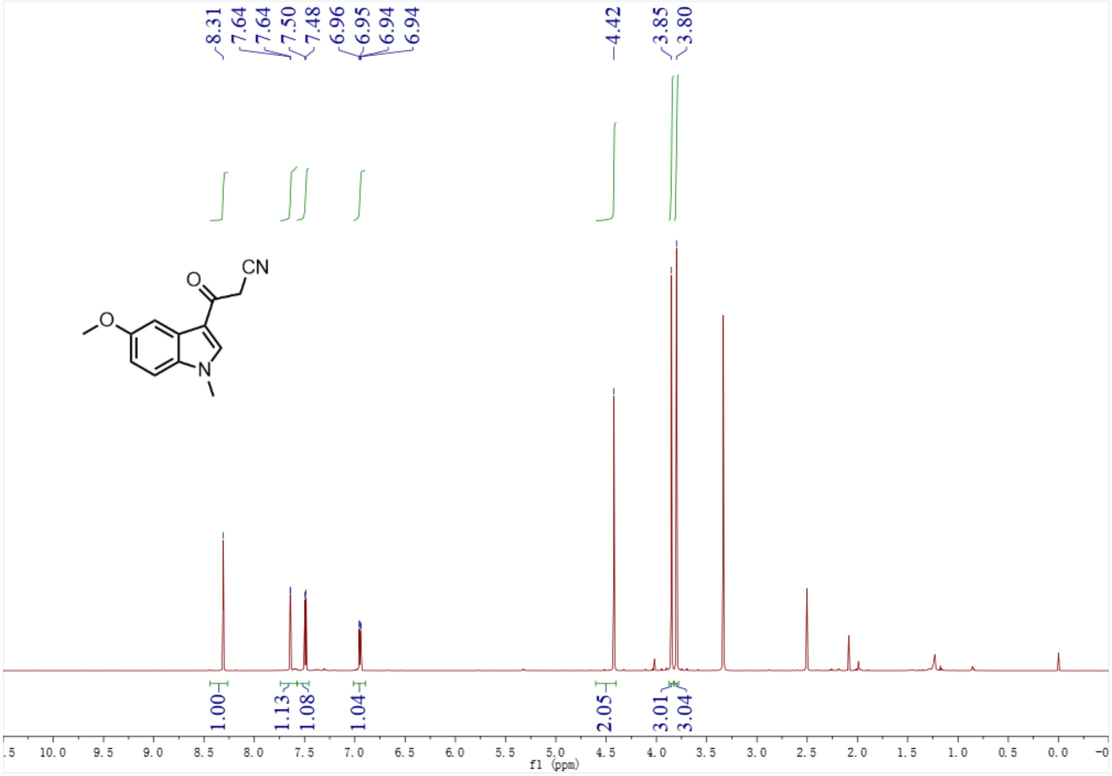

^{13}C NMR spectrum of **3g** (DMSO-d_6)

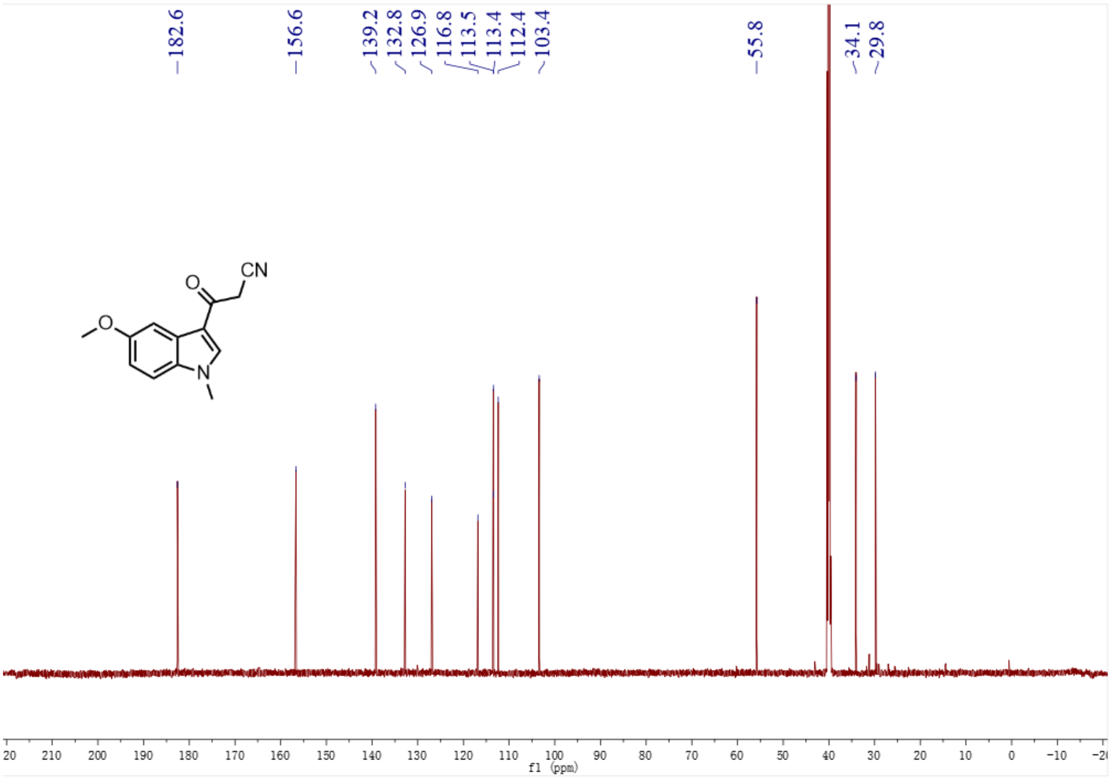

^{1}H NMR spectrum of **3h** (DMSO-d_6)

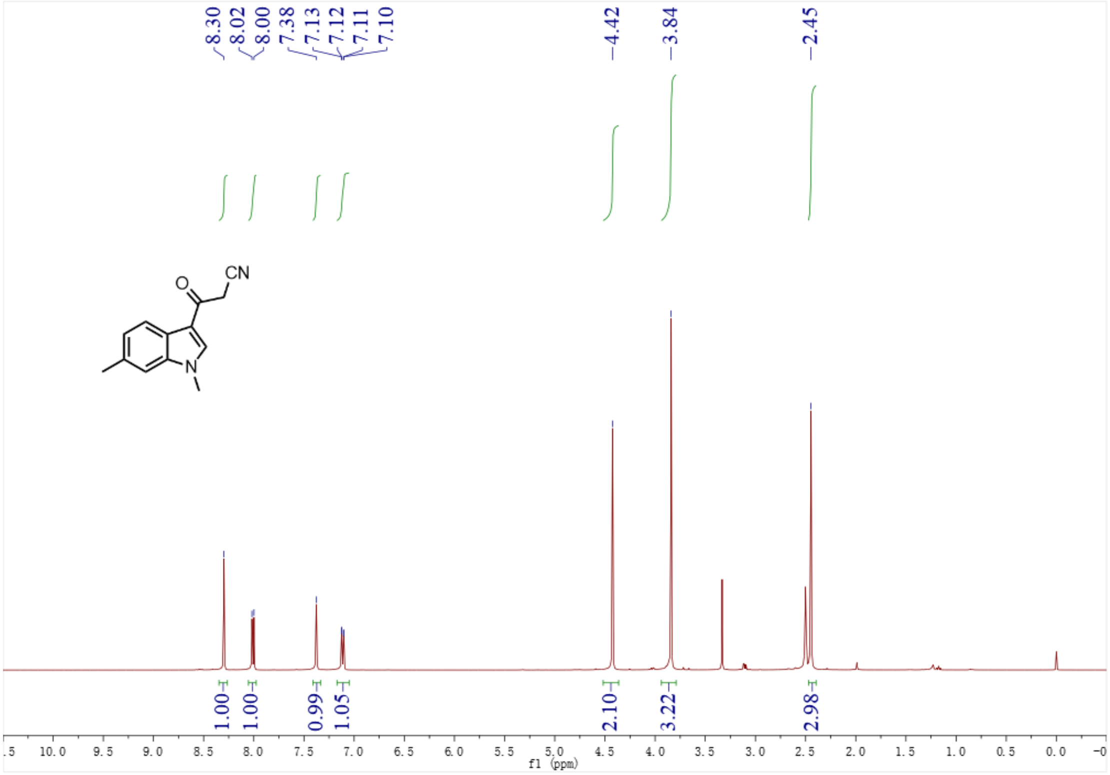

^{13}C NMR spectrum of **3h** (DMSO-d_6)

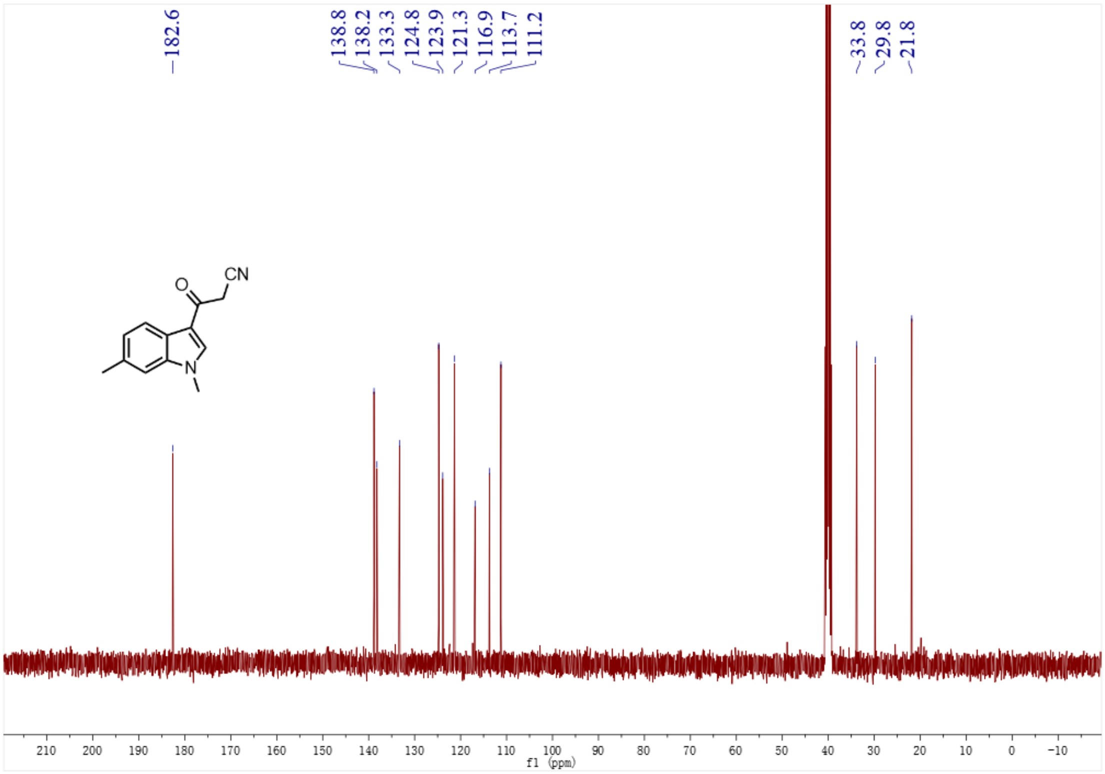

^{1}H NMR spectrum of **3j** ($CDCl_3$)

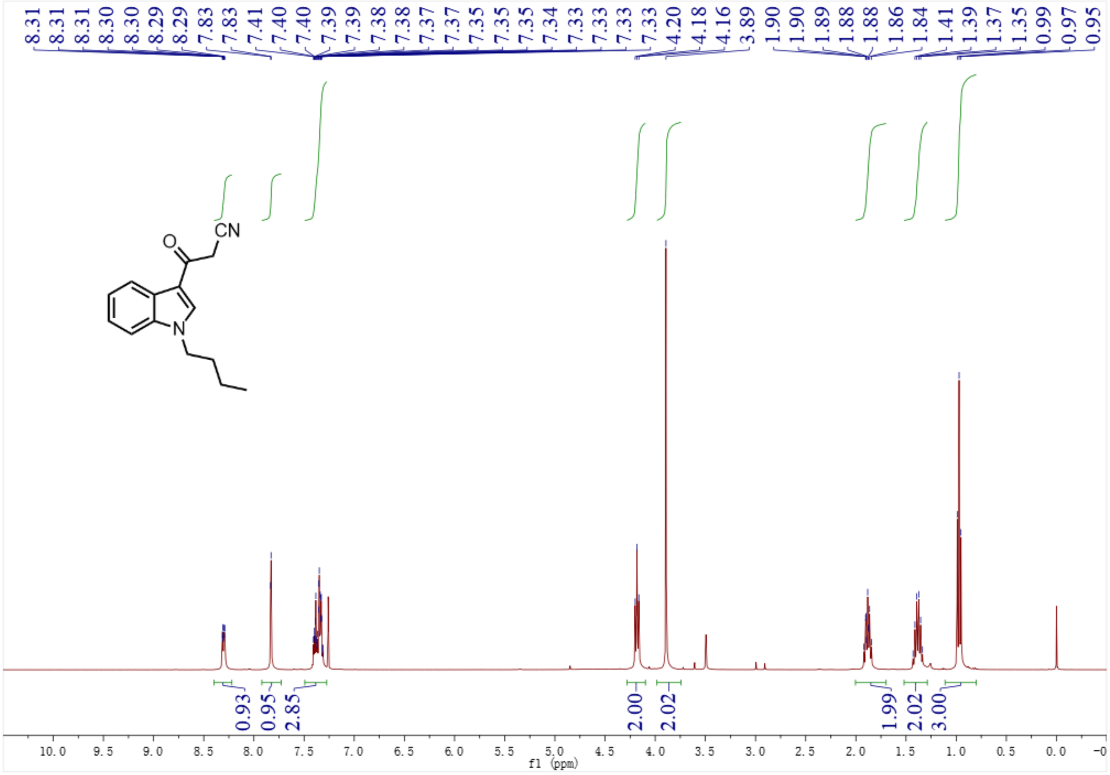

^{13}C NMR spectrum of **3j** ($CDCl_3$)

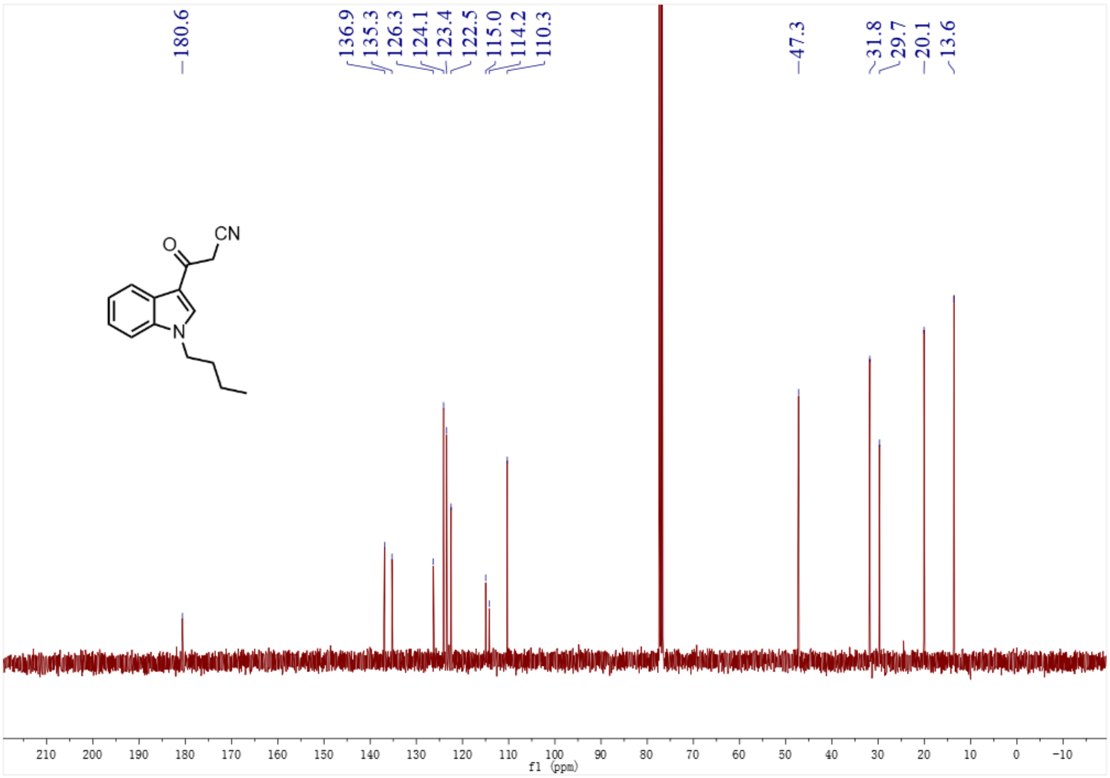

^{1}H NMR spectrum of **3k** ($CDCl_3$)

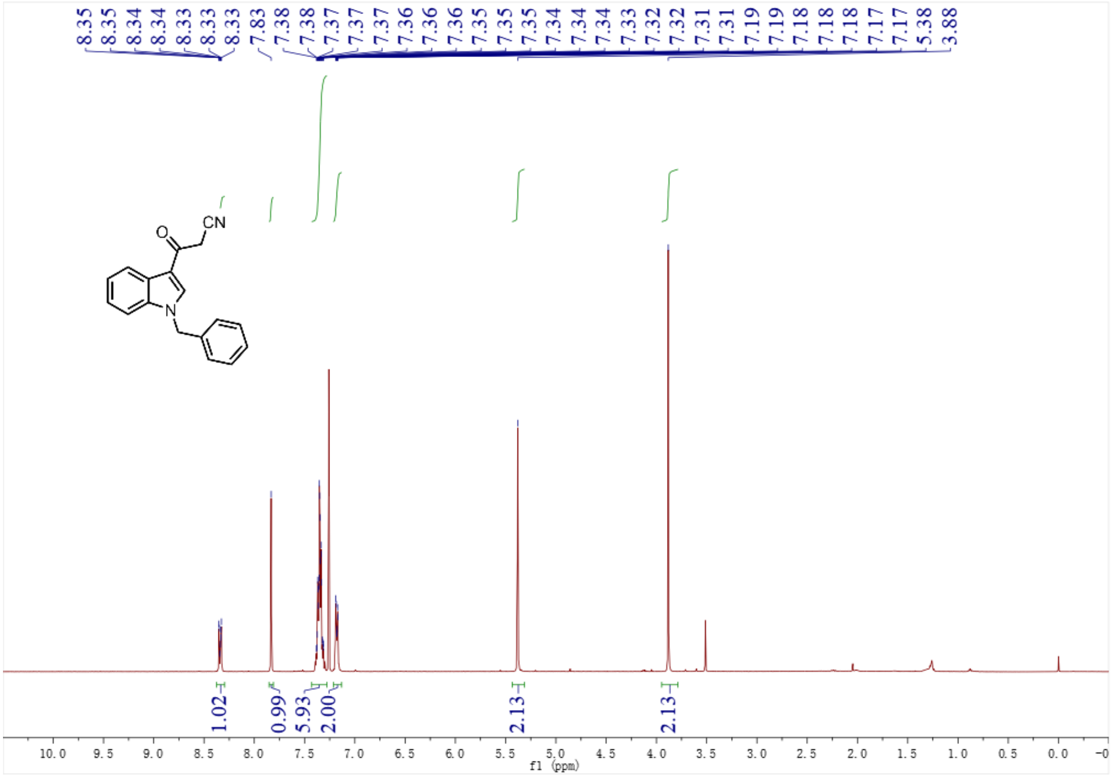

^{13}C NMR spectrum of **3k** ($CDCl_3$)

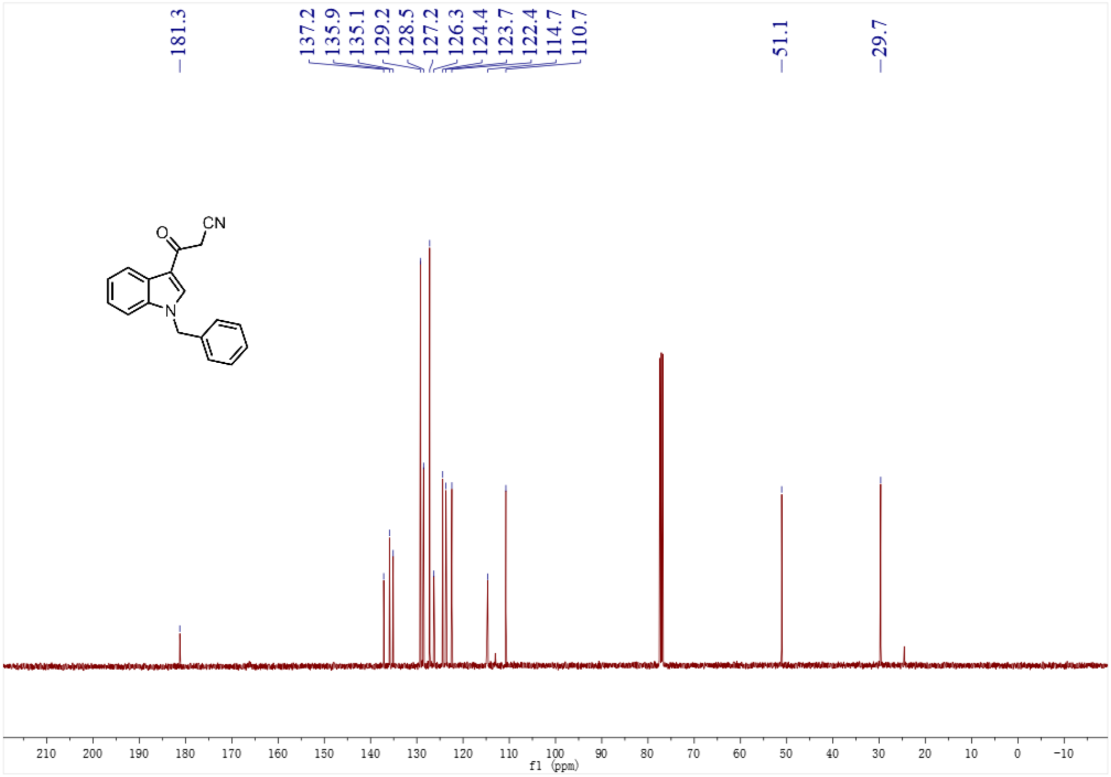

^{1}H NMR spectrum of **3l** (DMSO-d_6)

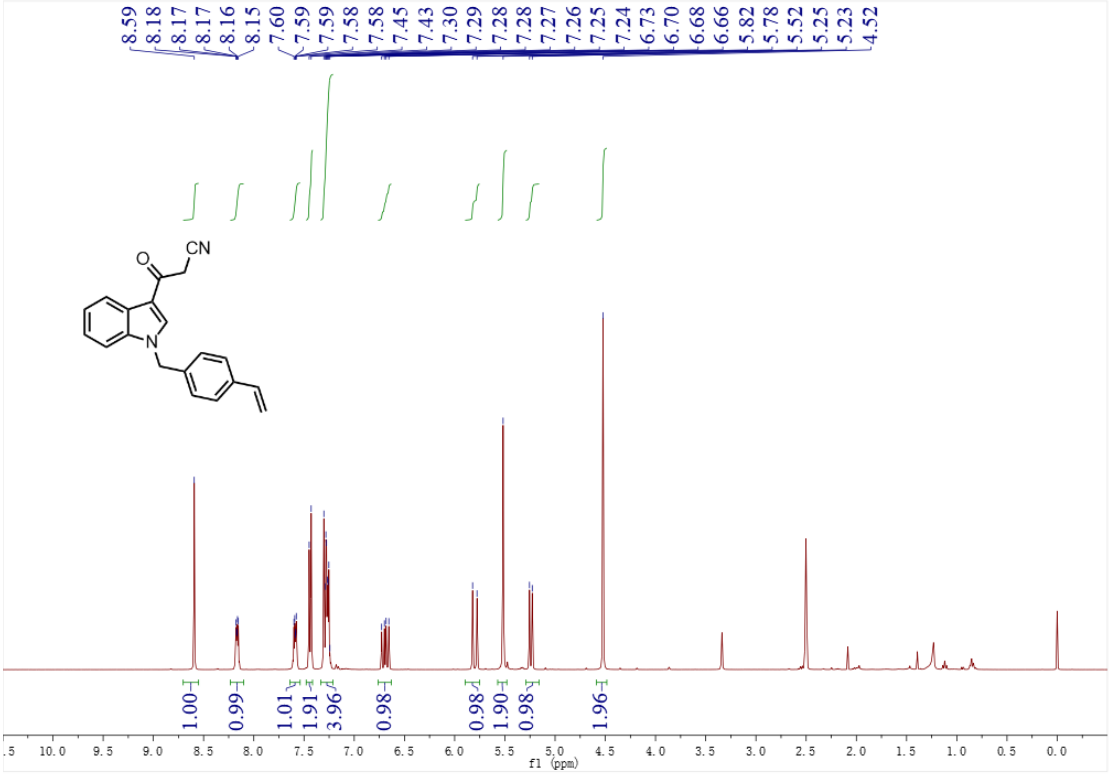

^{13}C NMR spectrum of **3l** (DMSO-d_6)

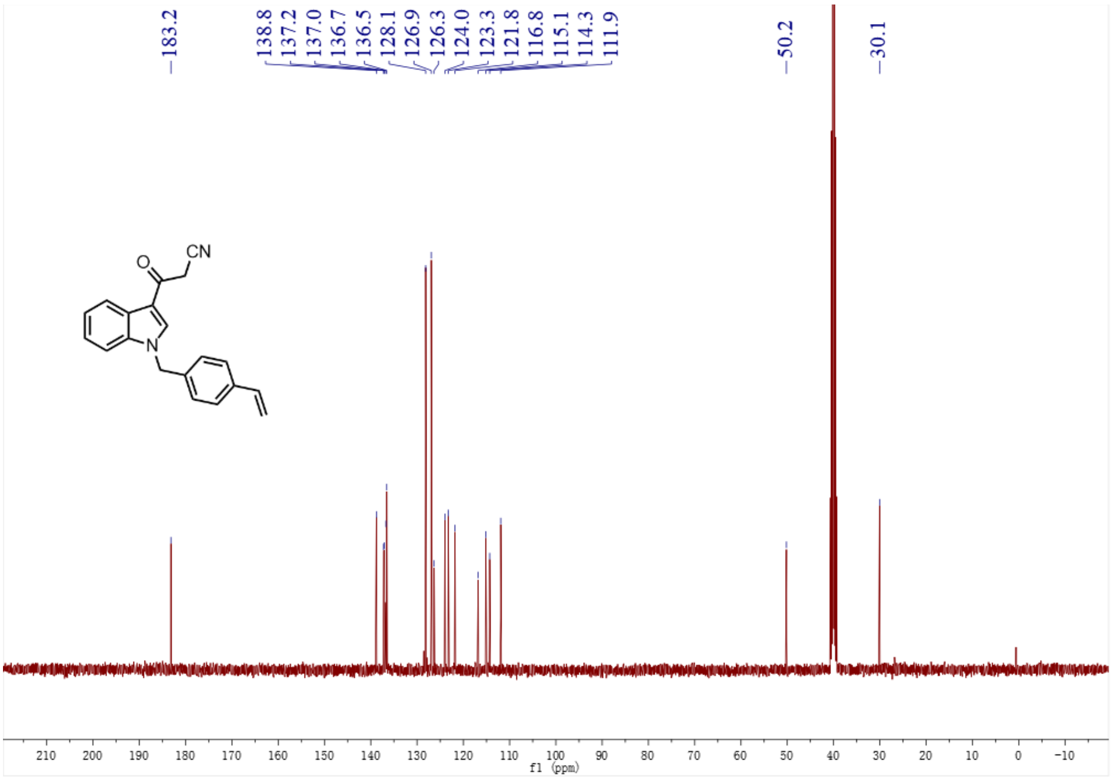

^{1}H NMR spectrum of **3m** ($CDCl_3$)

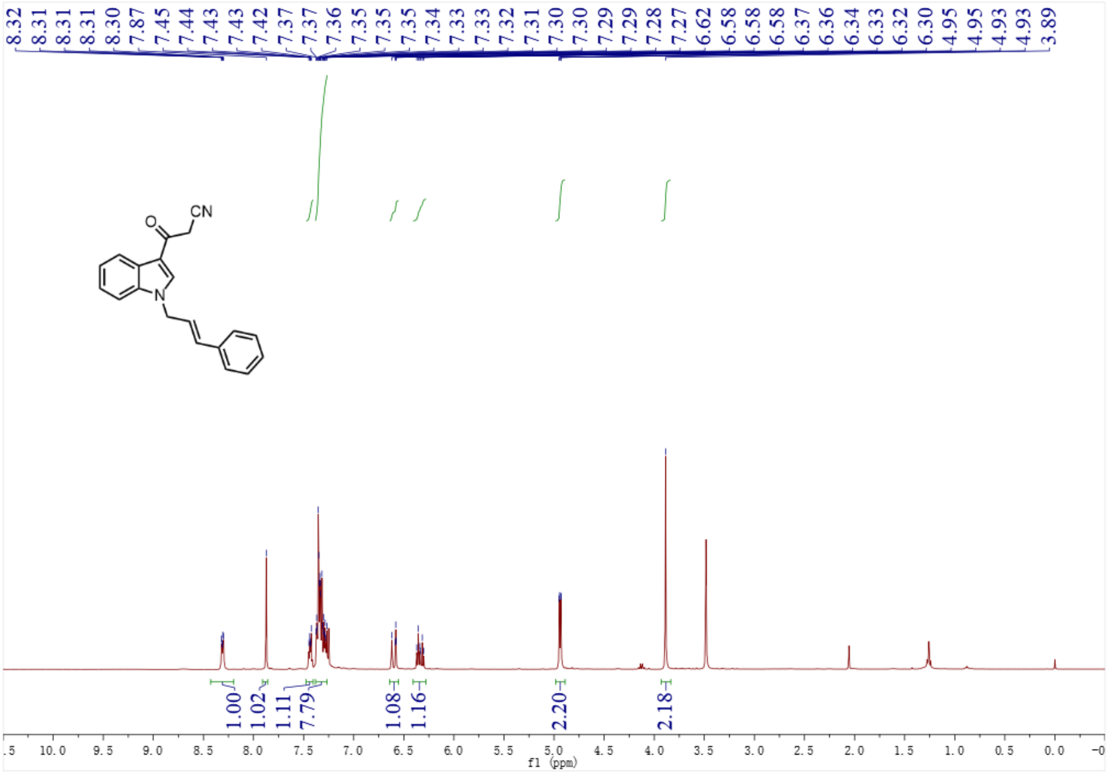

^{13}C NMR spectrum of **3m** ($CDCl_3$)

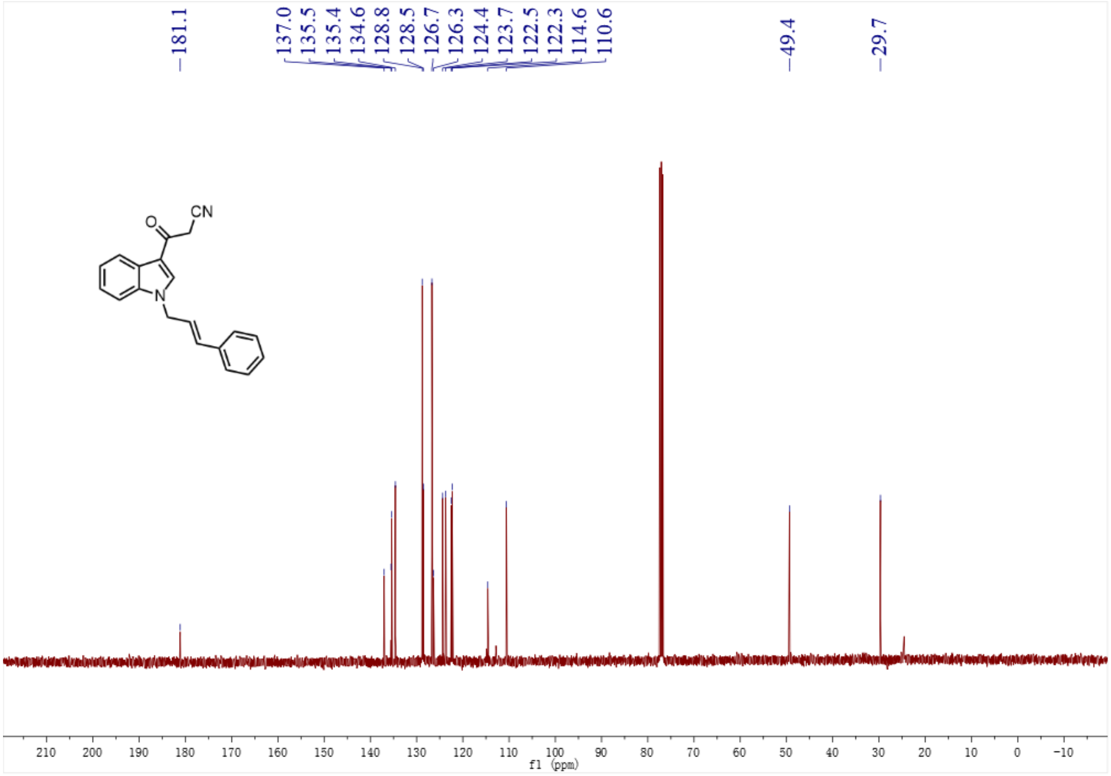

^{1}**H NMR** spectrum of **3n** ($CDCl_3$)

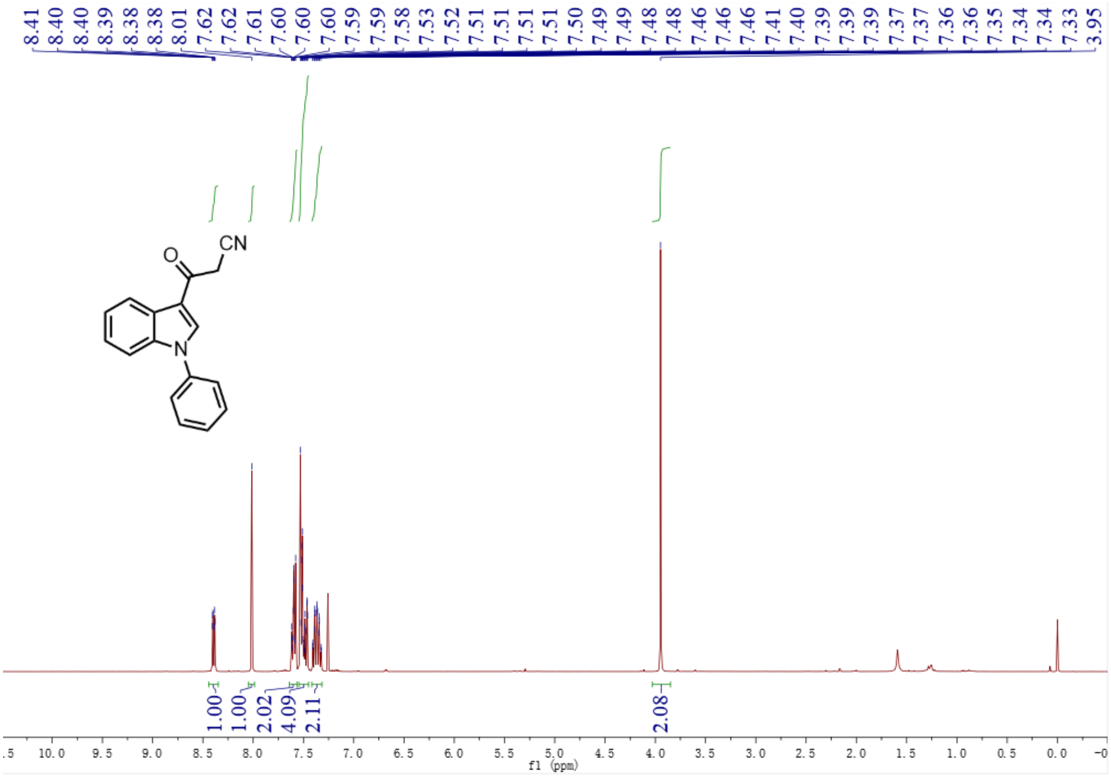

^{13}**C NMR** spectrum of **3n** ($CDCl_3$)

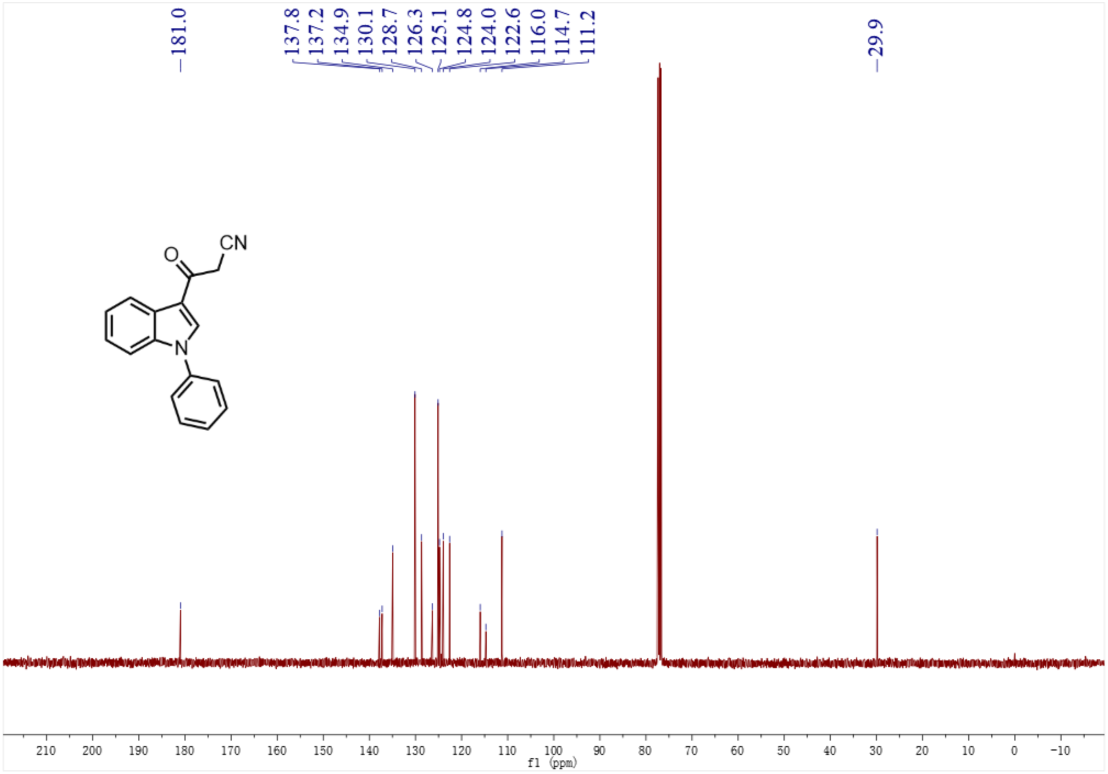

^{1}**H NMR** spectrum of **3o** ($CDCl_3$)

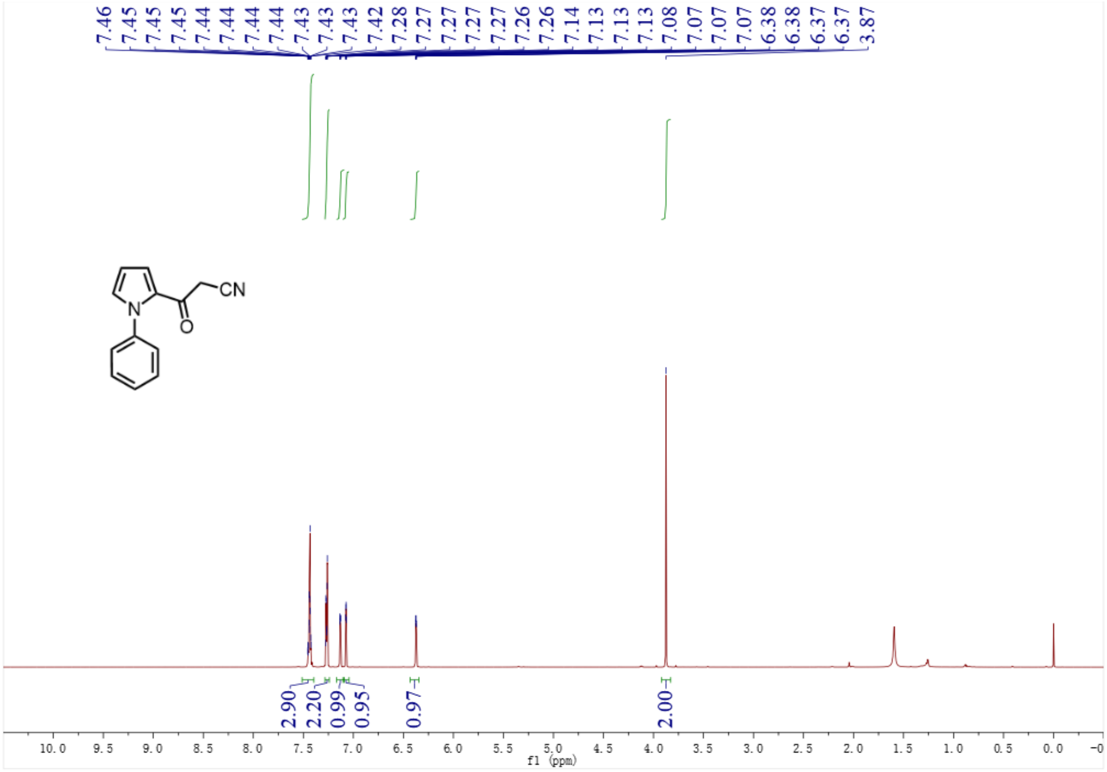

^{13}**C NMR** spectrum of **3o** ($CDCl_3$)

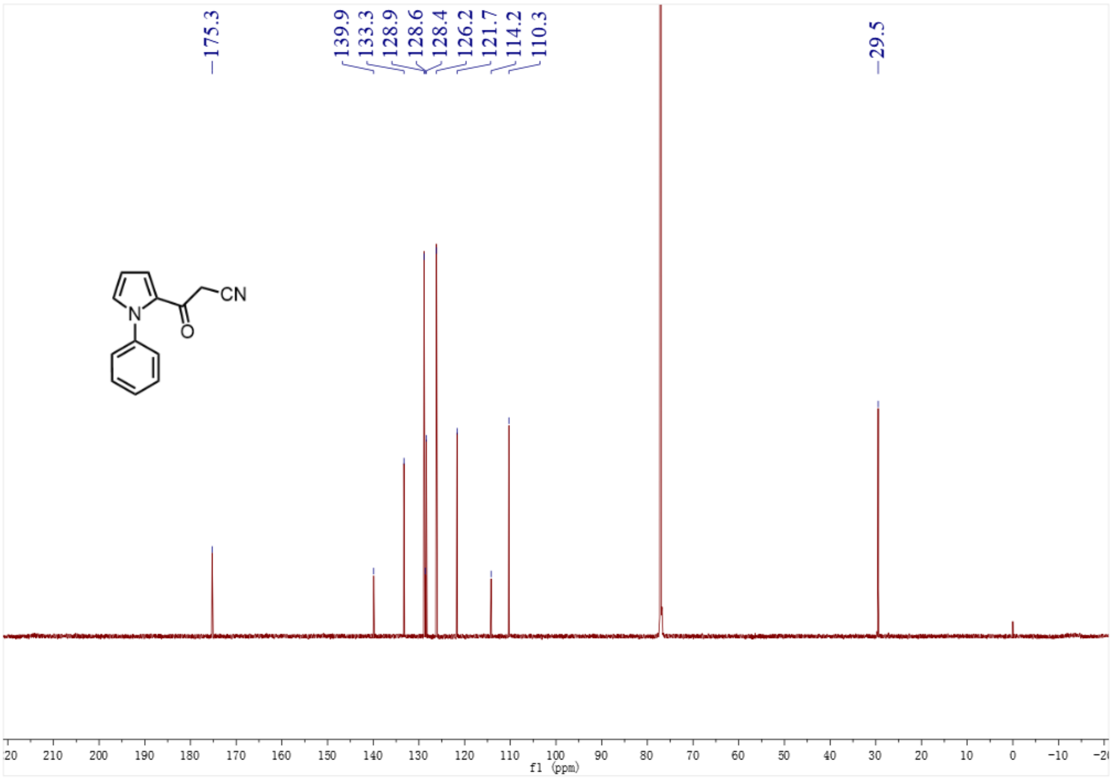

^{1}H NMR spectrum of **4a** (DMSO-d_6)

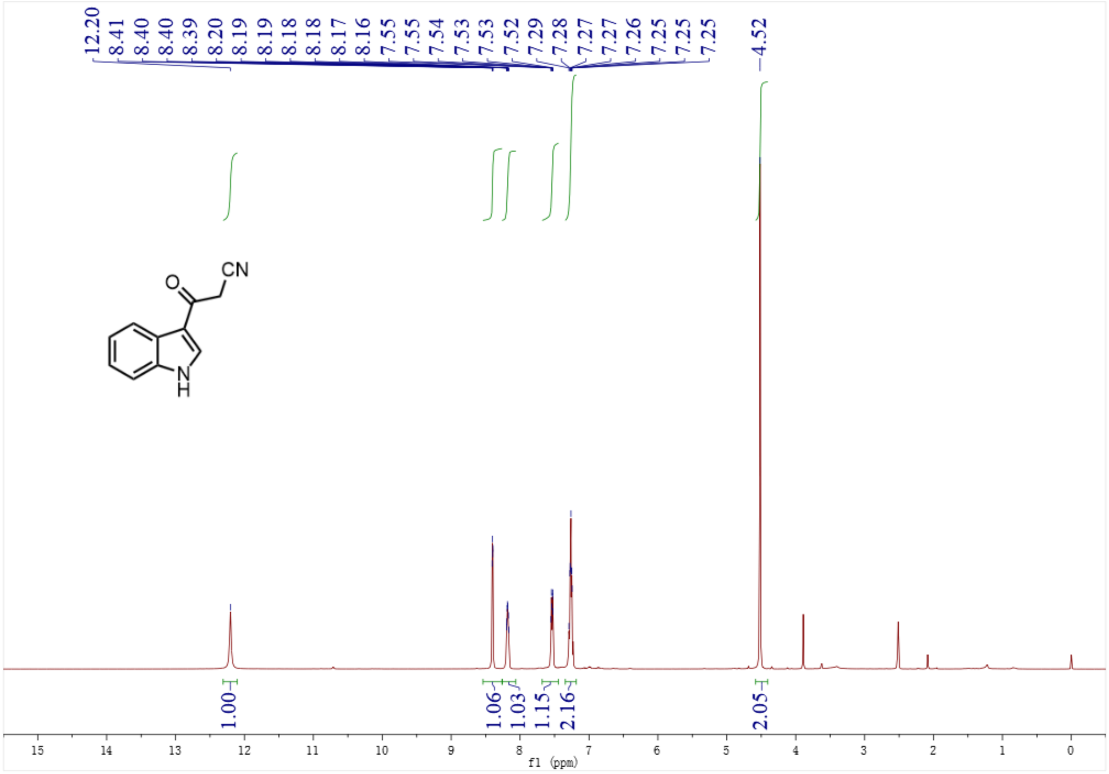

^{13}C NMR spectrum of **4a** (DMSO-d_6)

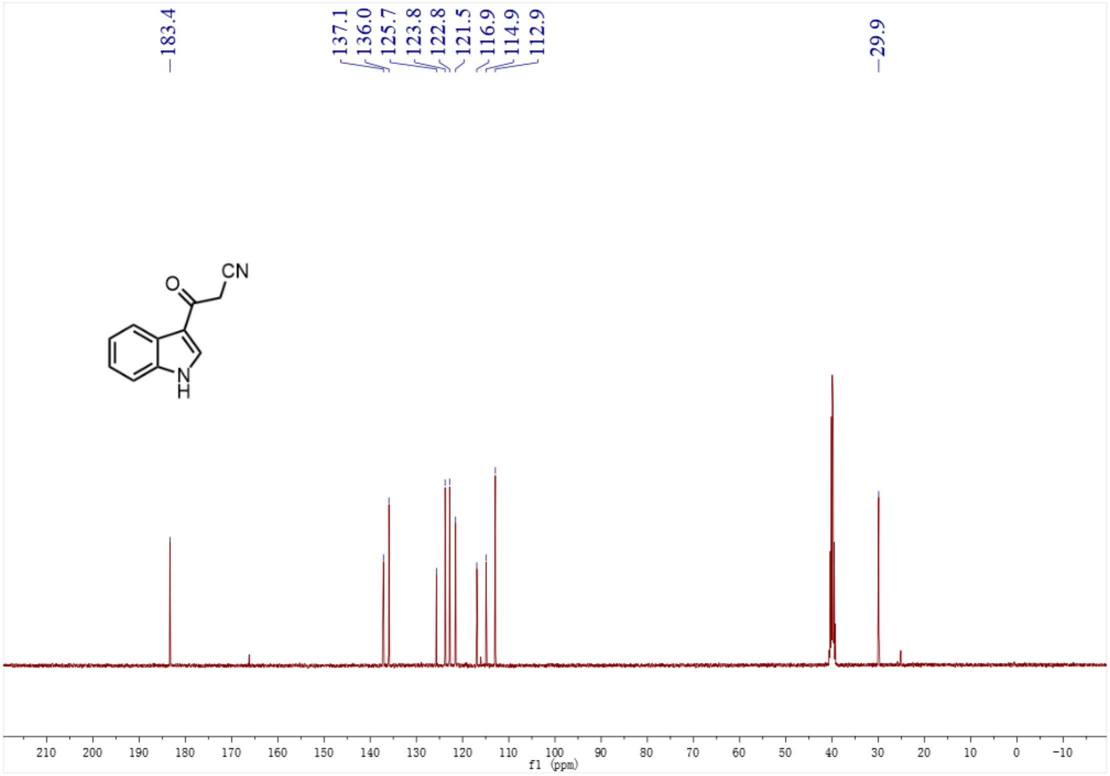

^{1}H NMR spectrum of **4b** (DMSO-d_6)

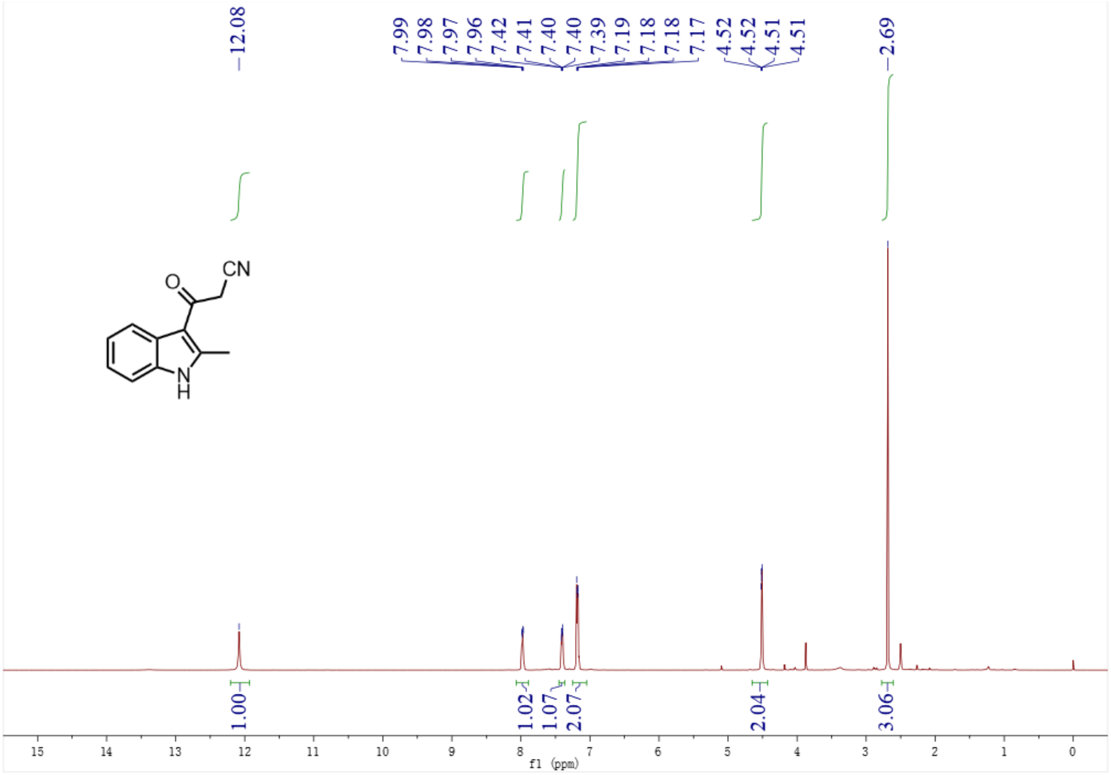

^{13}C NMR spectrum of **4b** (DMSO-d_6)

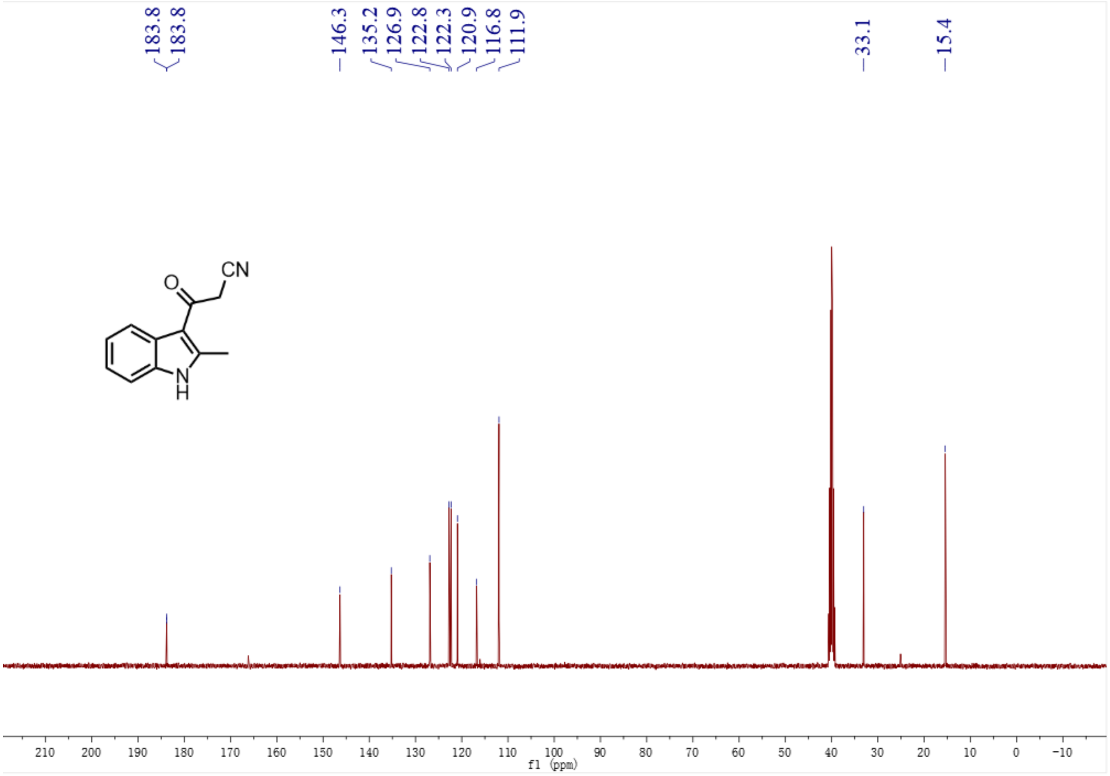

^{1}H NMR spectrum of **4c** (DMSO-d_6)

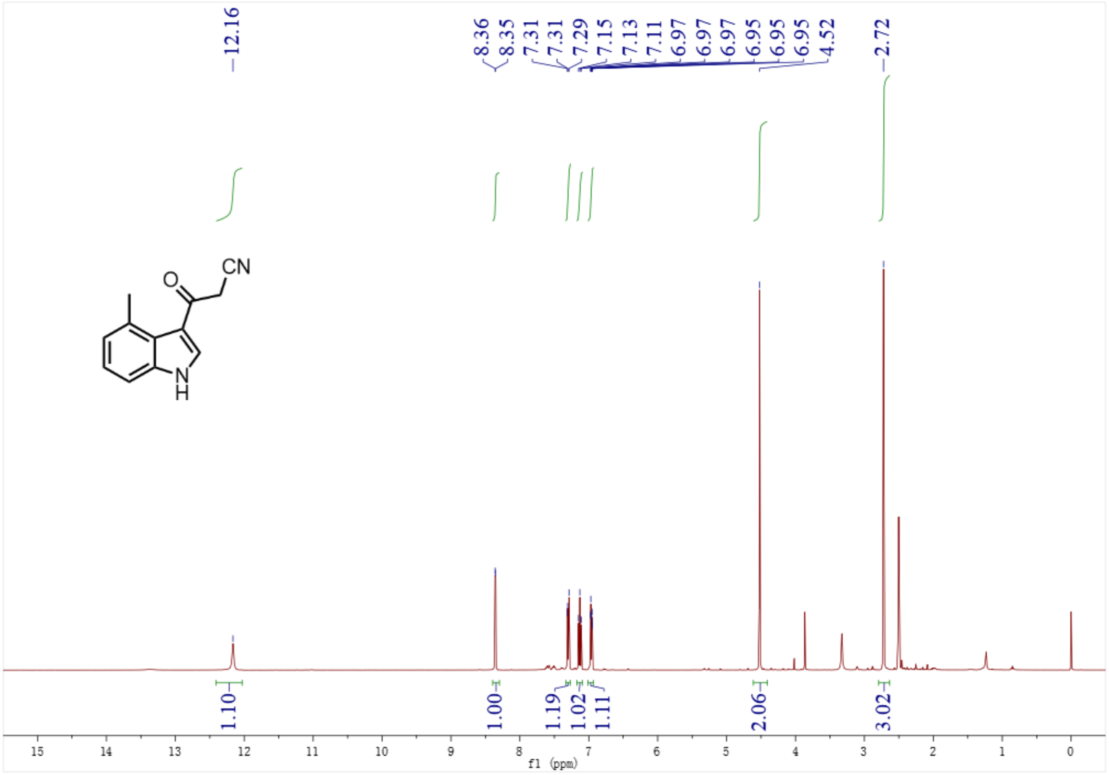

^{13}C NMR spectrum of **4c** (DMSO-d_6)

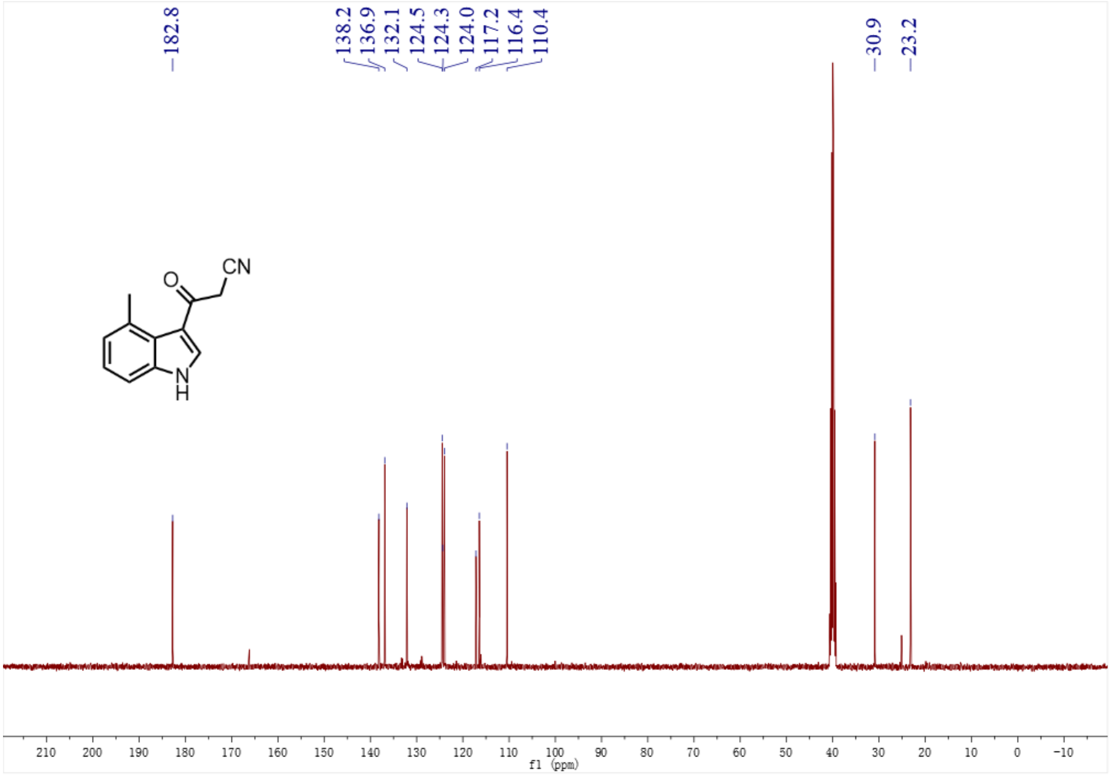

^{1}**H NMR** spectrum of **4d** (DMSO-d_6)

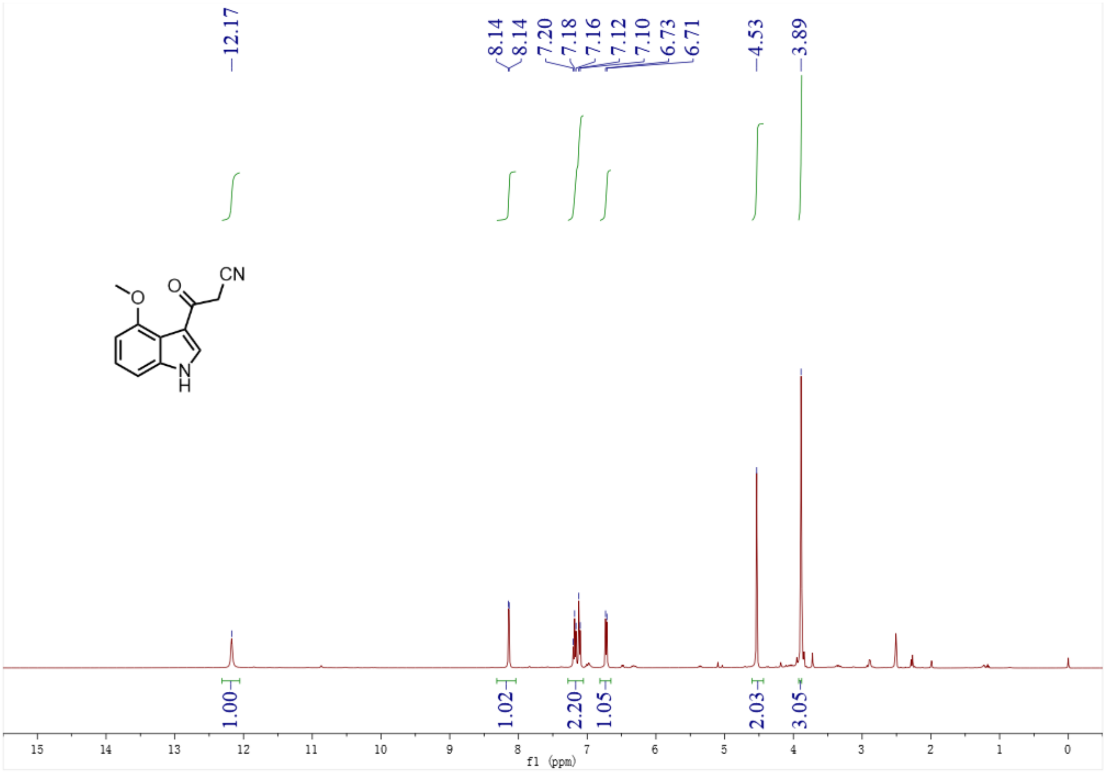

^{13}**C NMR** spectrum of **4d** (DMSO-d_6)

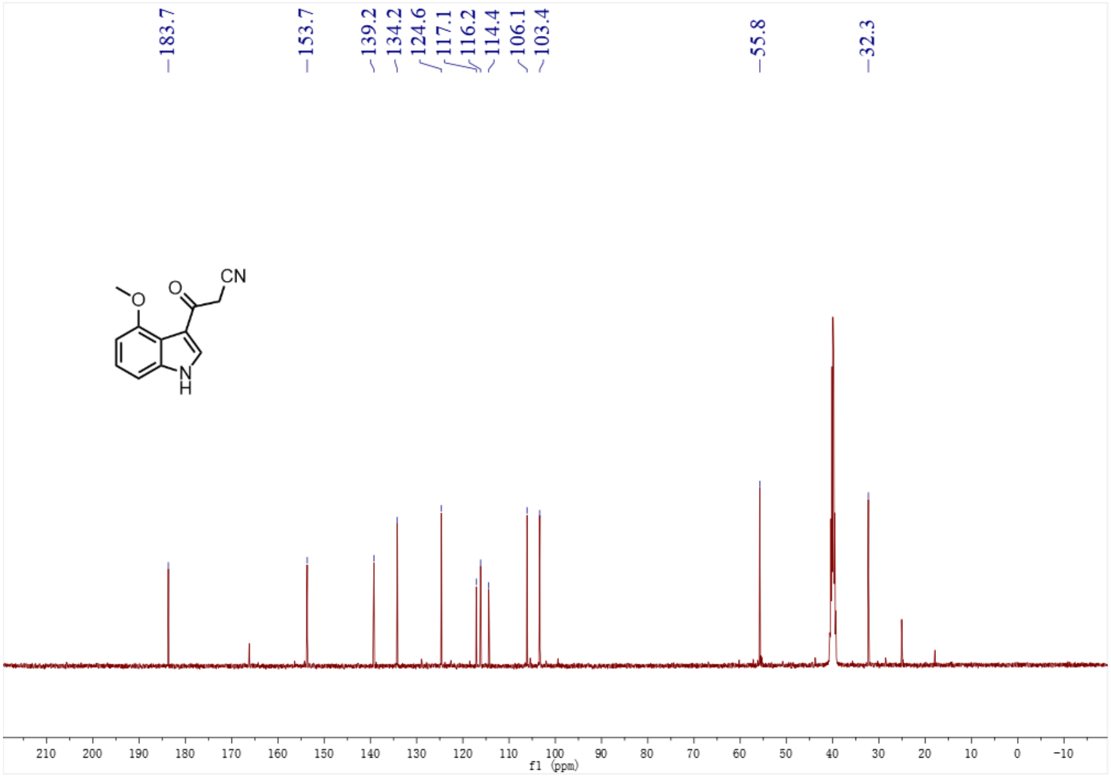

^{1}H NMR spectrum of **4f** (DMSO-d_6)

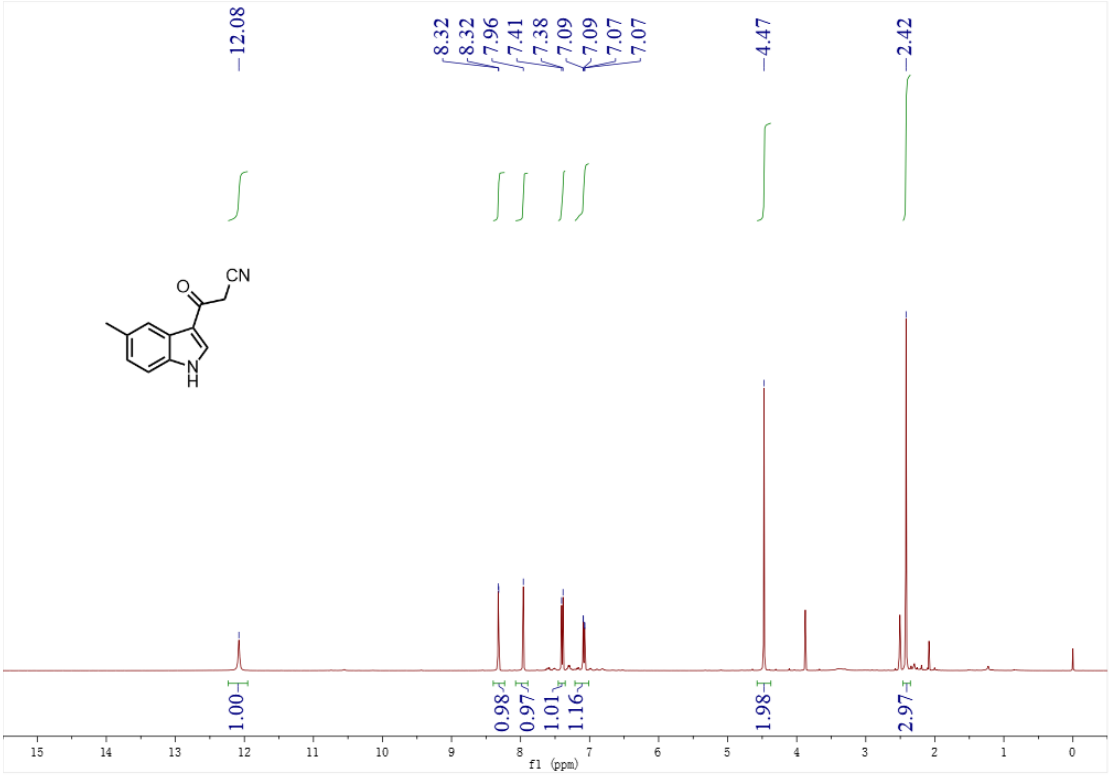

^{13}C NMR spectrum of **4f** (DMSO-d_6)

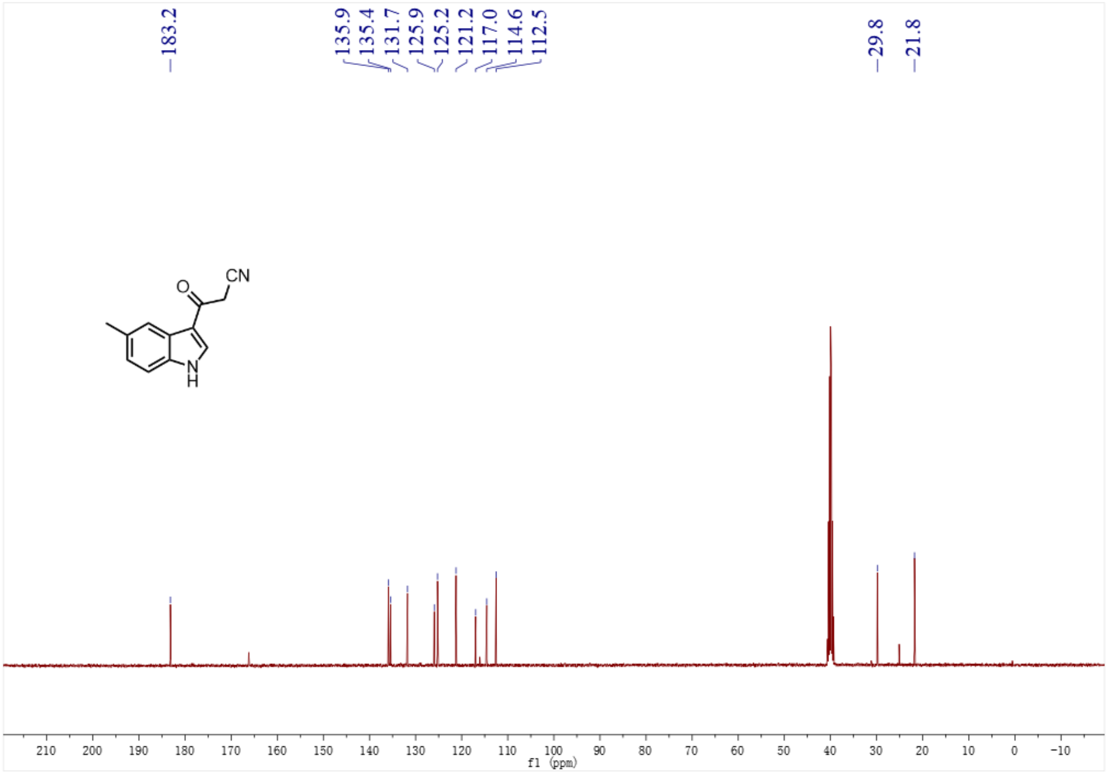

^{1}H NMR spectrum of **4g** (DMSO-d_6)

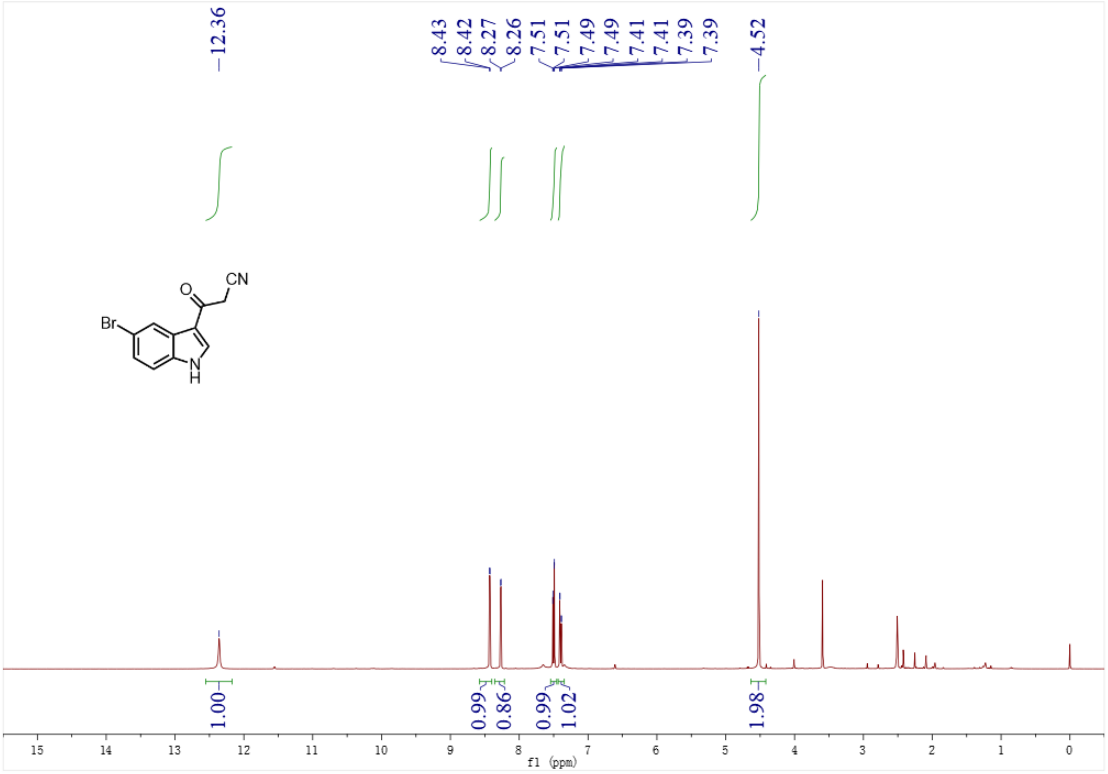

^{13}C NMR spectrum of **4g** (DMSO-d_6)

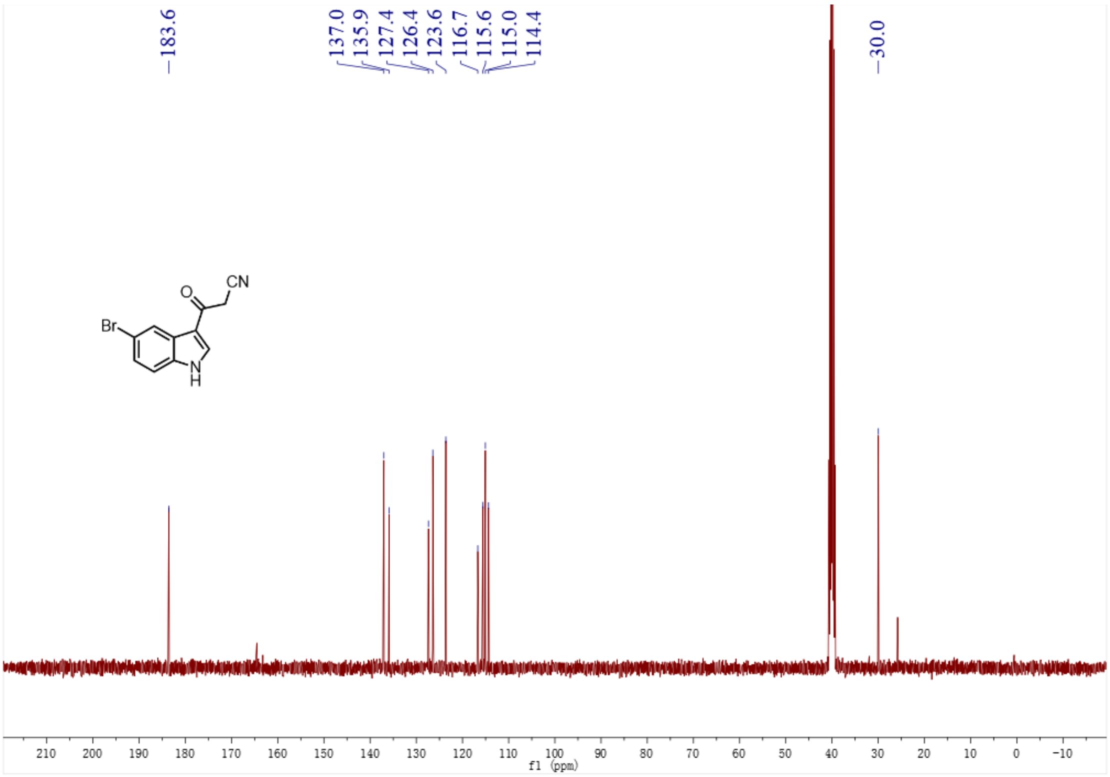

^{1}H NMR spectrum of **4h** (DMSO-d_6)

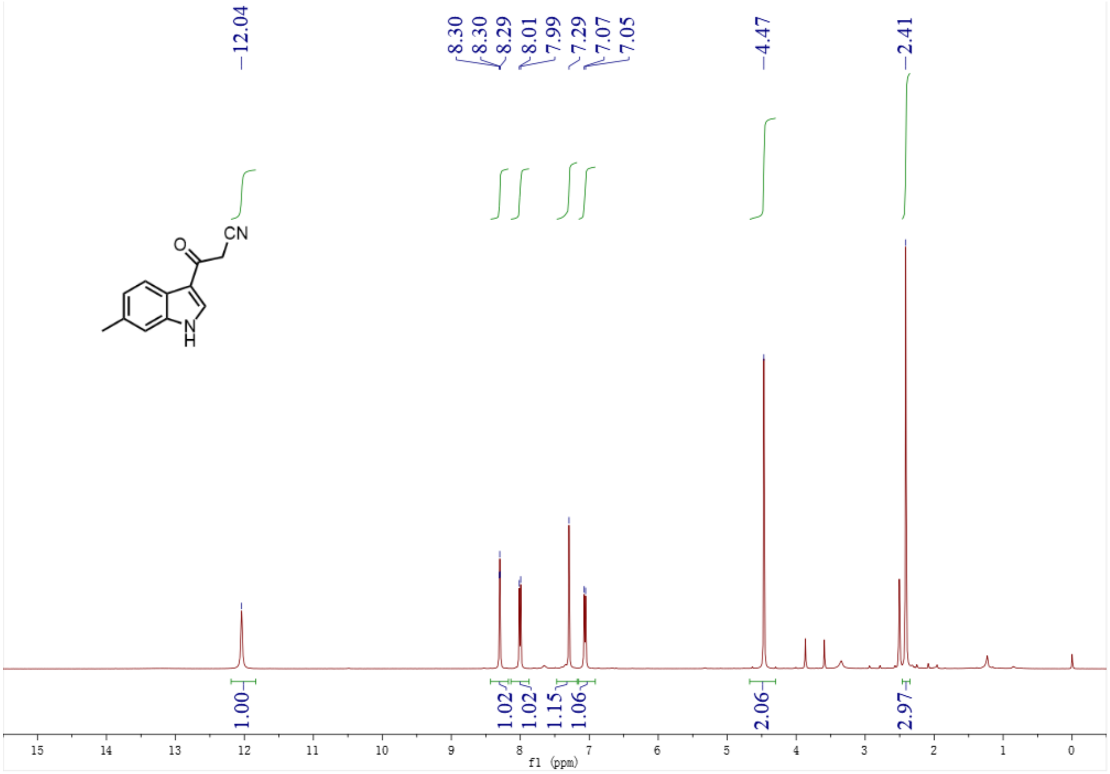

^{13}C NMR spectrum of **4h** (DMSO-d_6)

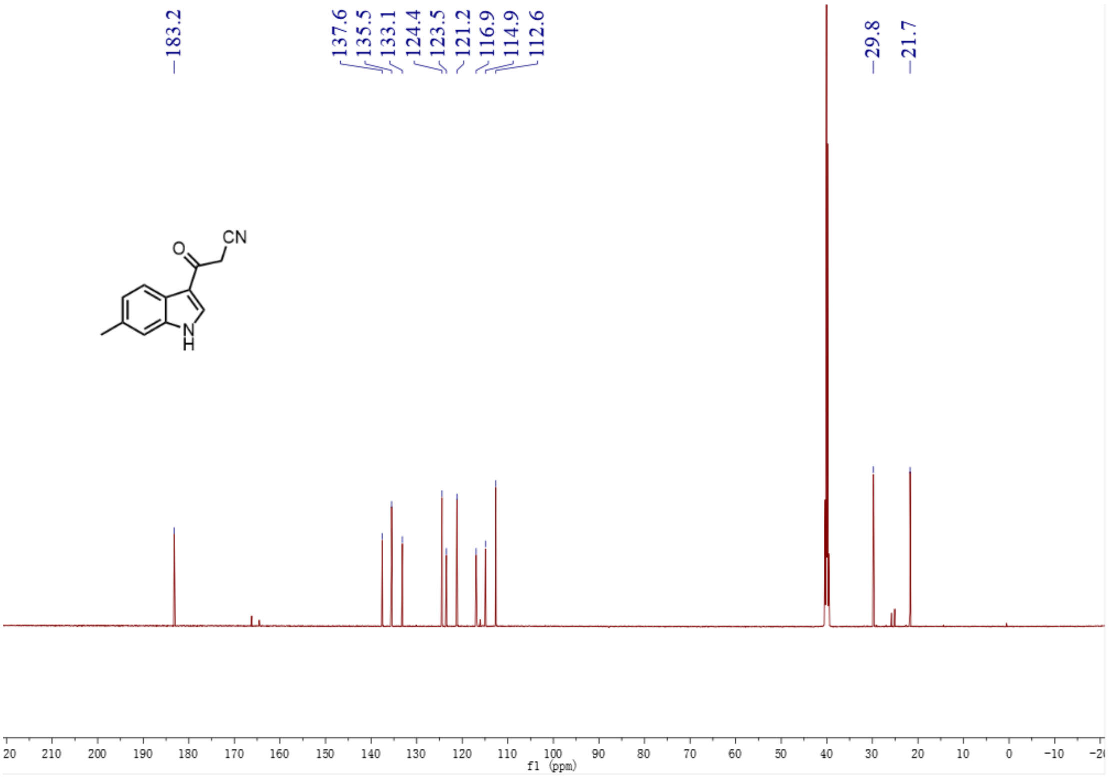

[1]**H NMR** spectrum of **4i** (DMSO-d_6)

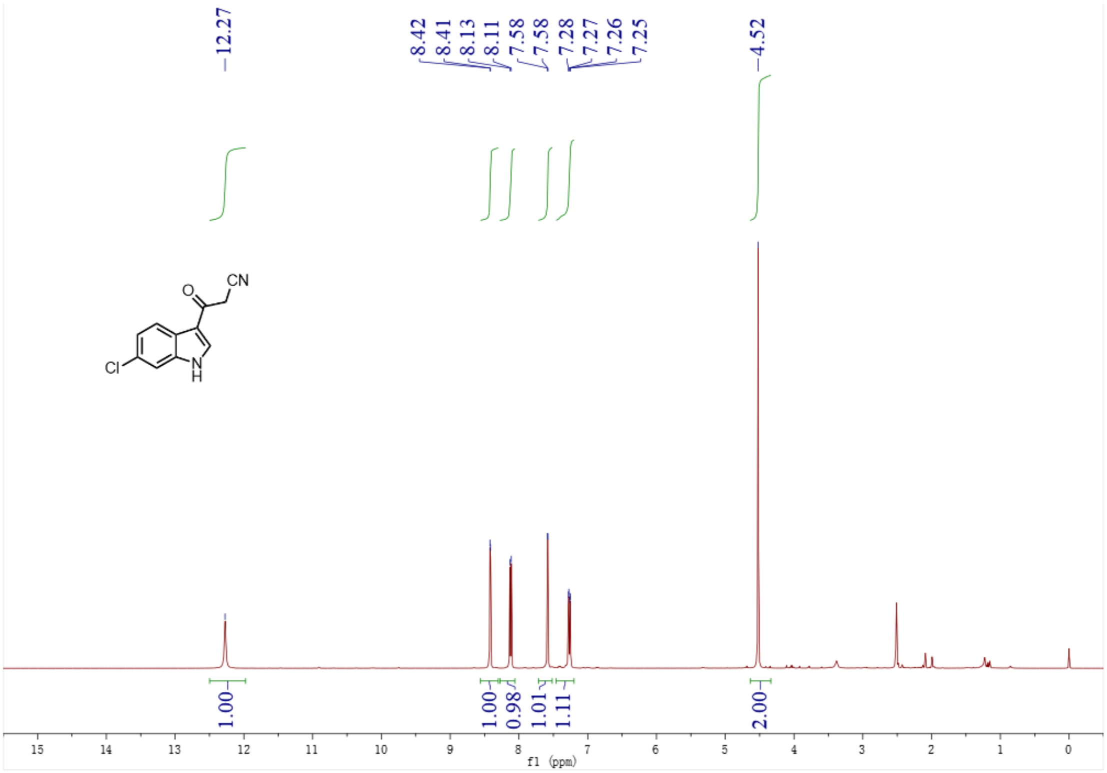

[13]**C NMR** spectrum of **4i** (DMSO-d_6)

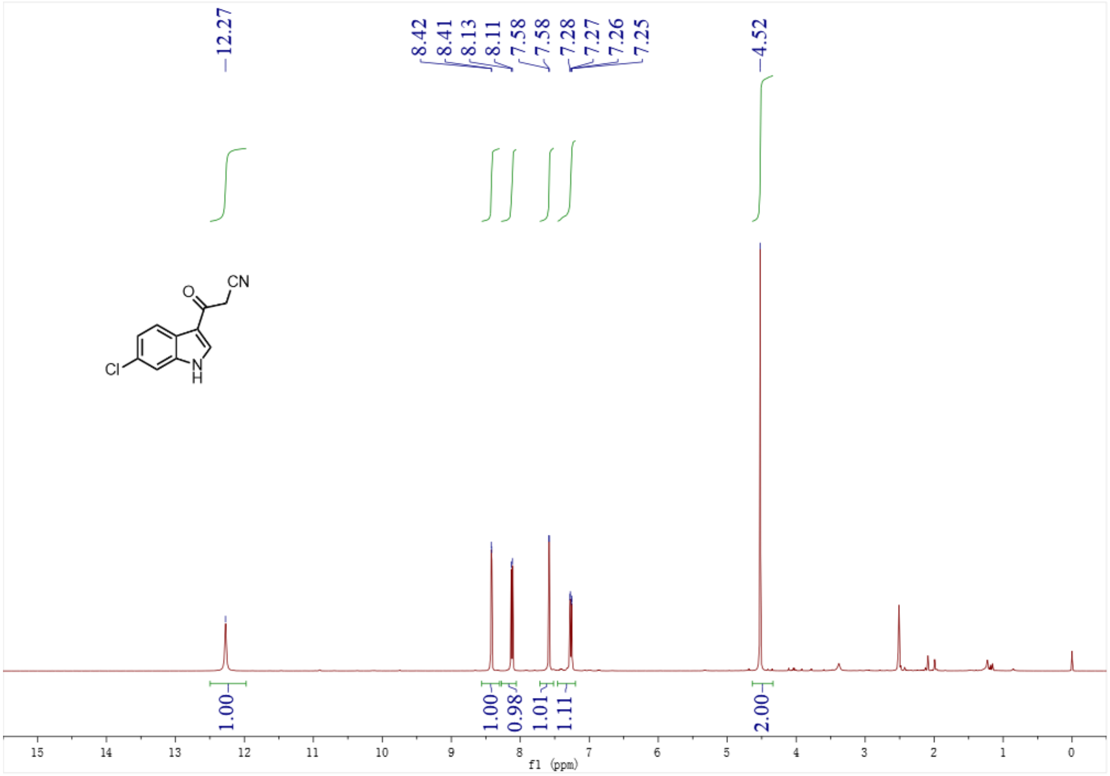

^{1}**H NMR** spectrum of **4j** (DMSO-d_6)

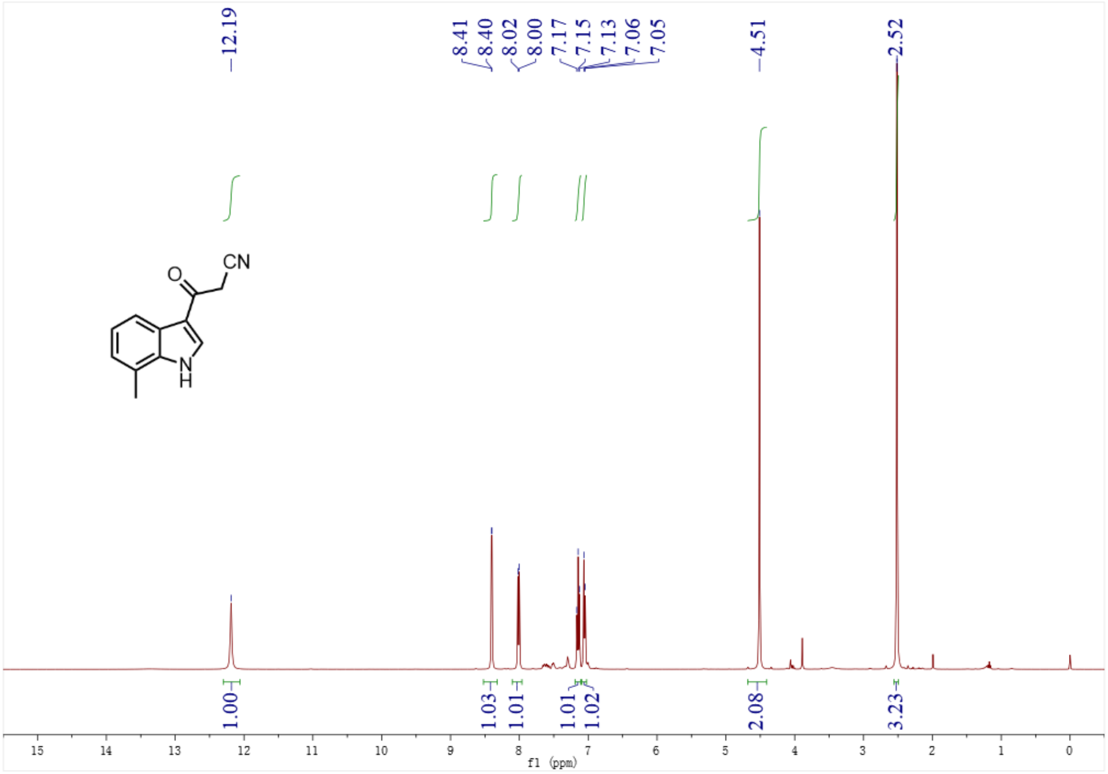

^{13}**C NMR** spectrum of **4j** (DMSO-d_6)

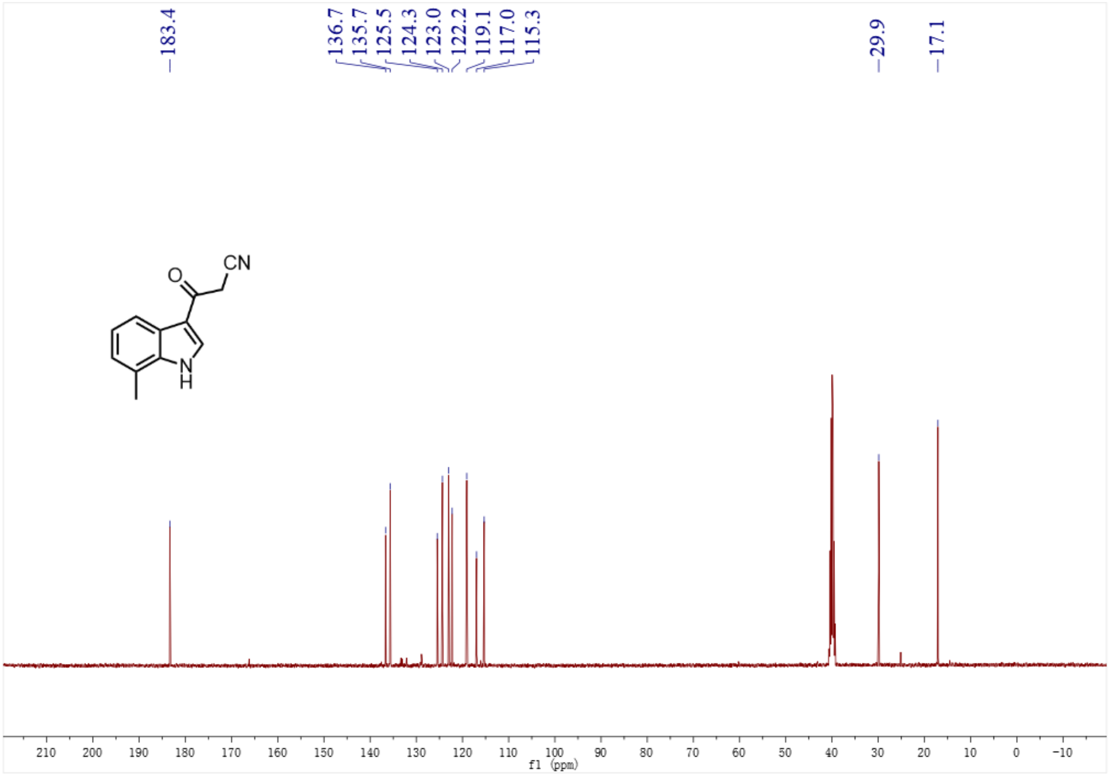

^{1}H NMR spectrum of **5a** ($CDCl_3$)

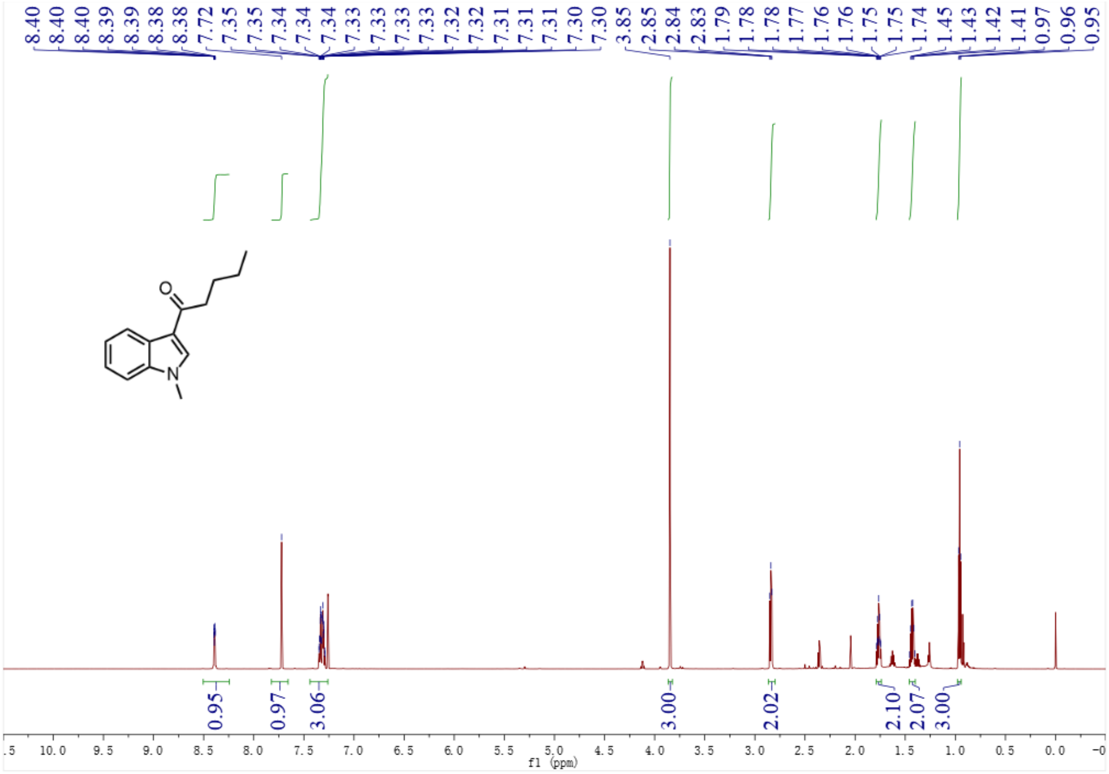

^{13}C NMR spectrum of **7a** ($CDCl_3$)

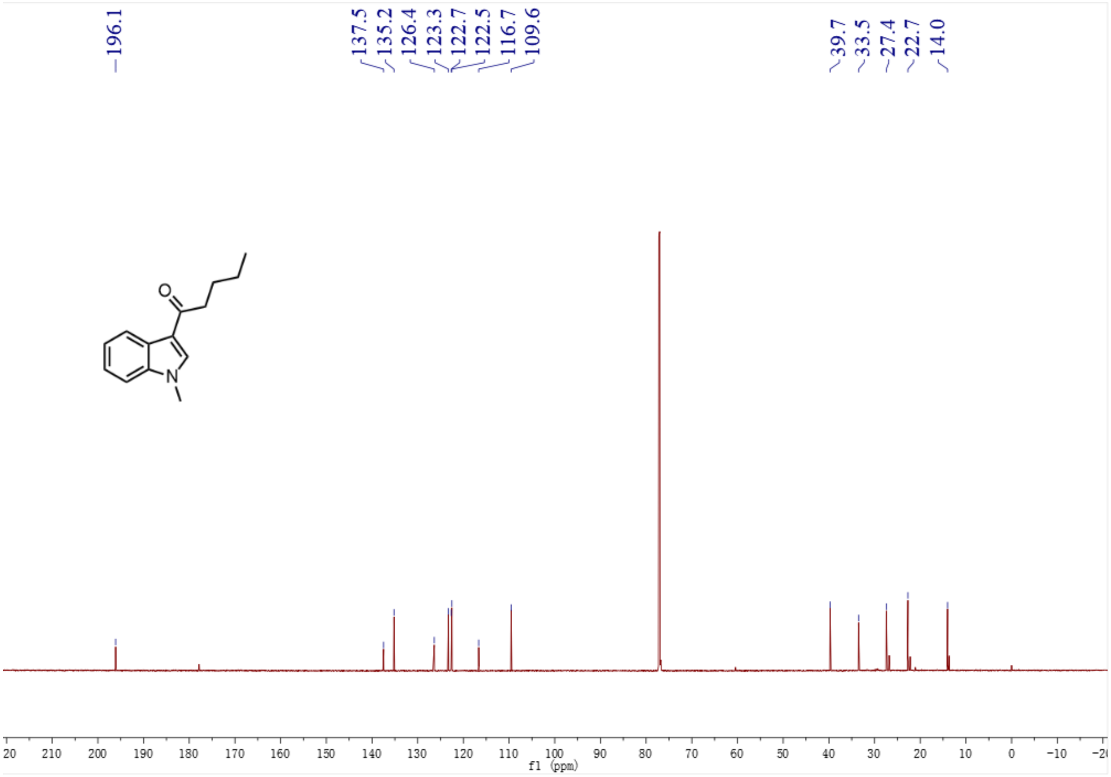

^{1}H NMR spectrum of 5c ($CDCl_3$)

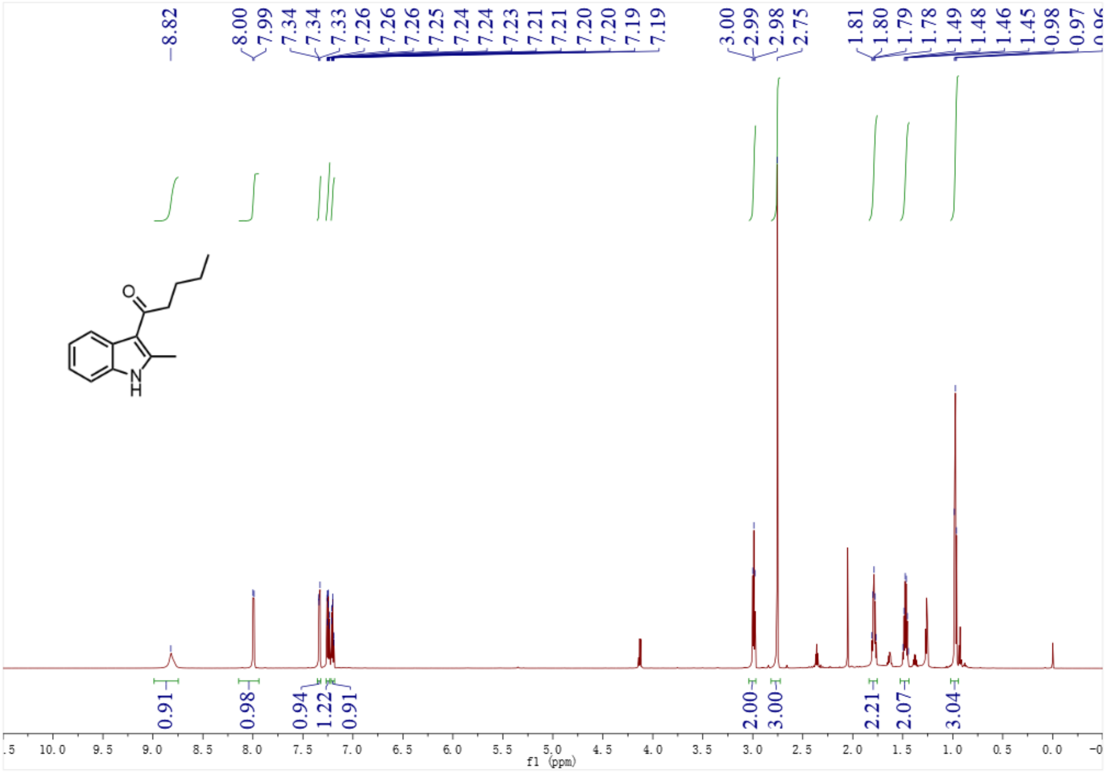

^{13}C NMR spectrum of 5c ($CDCl_3$)

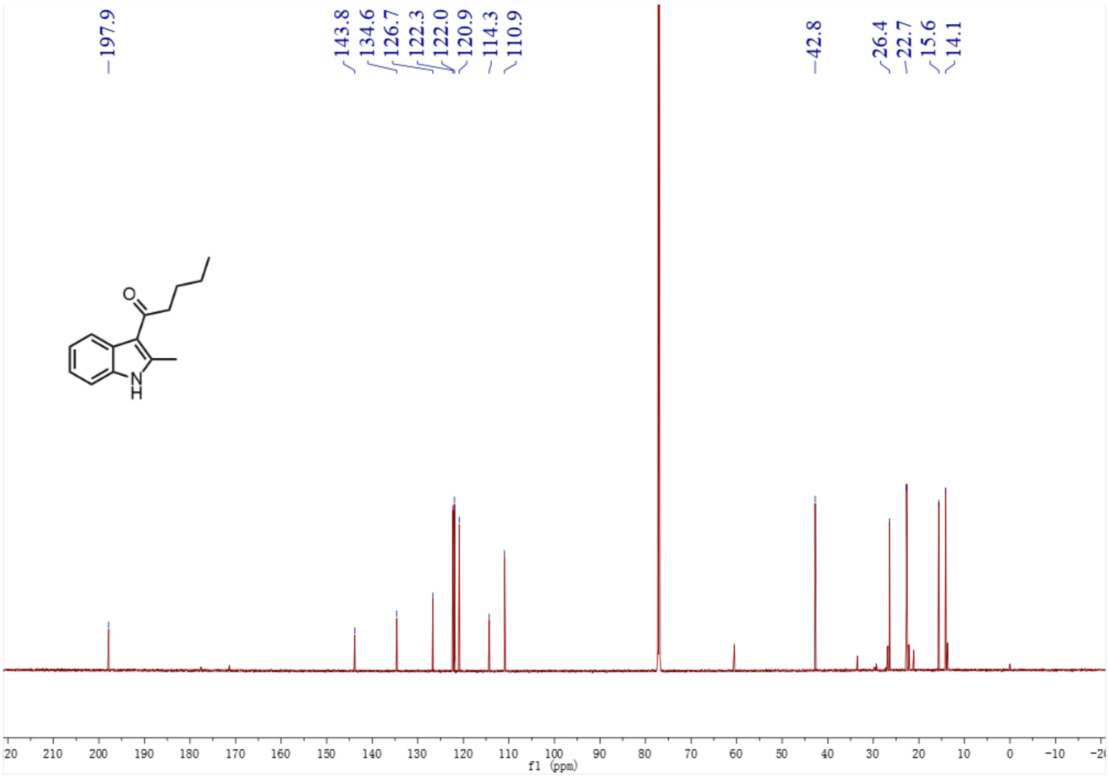

^{1}H NMR spectrum of **5d** ($CDCl_3$)

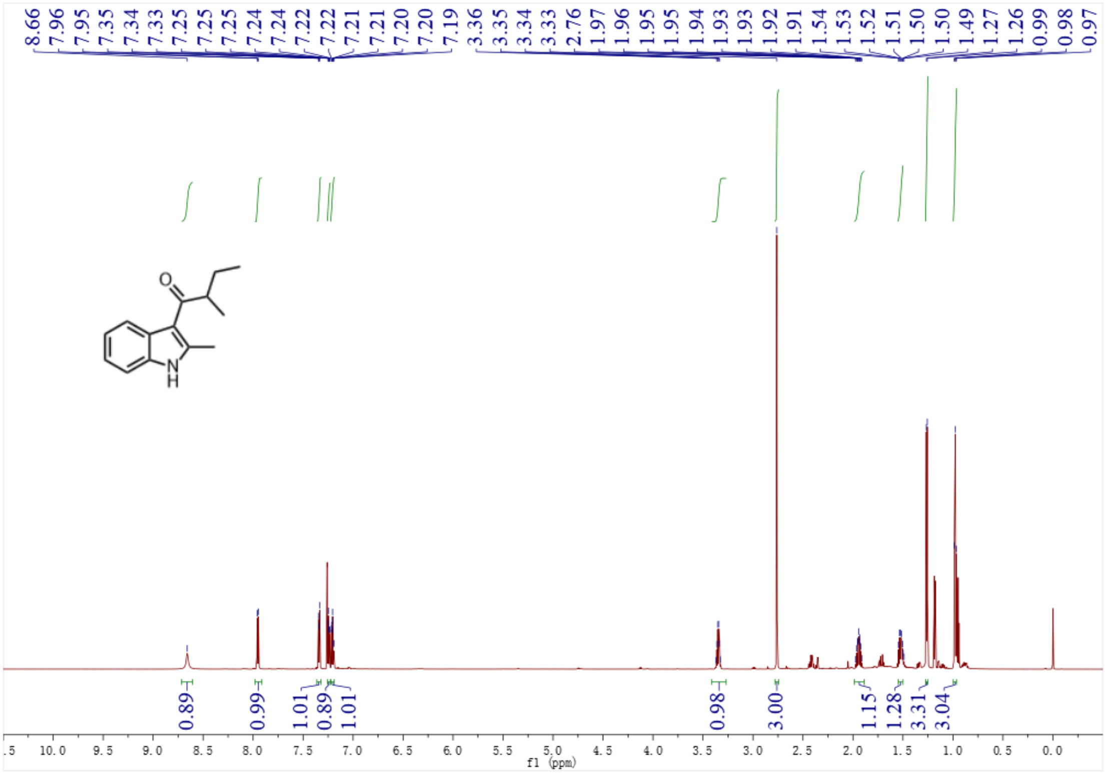

^{13}C NMR spectrum of **5d** ($CDCl_3$)

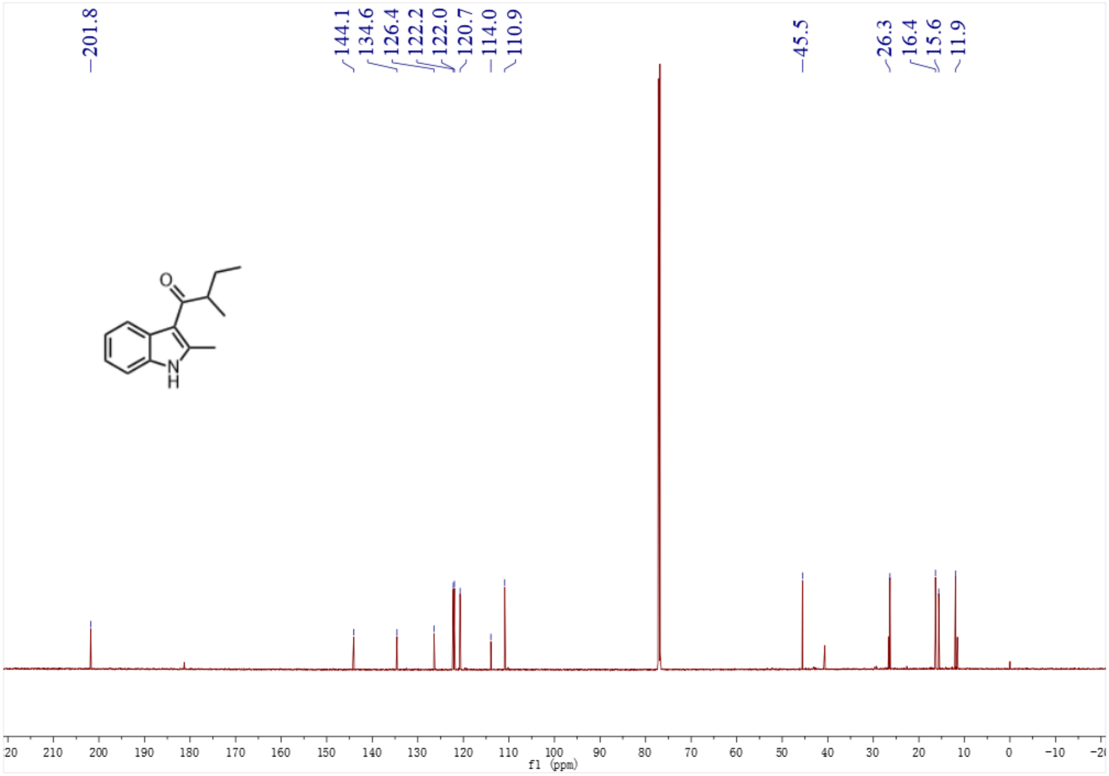

^{1}**H NMR** spectrum of **4aa** (DMSO-d_6)

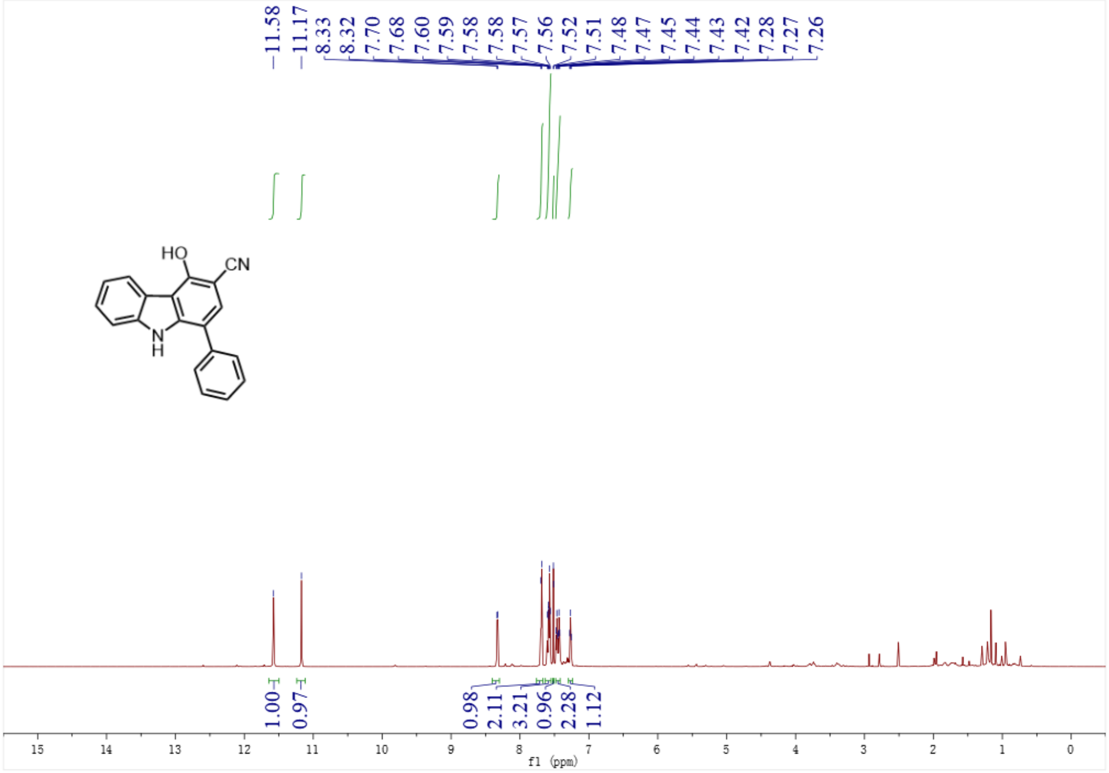

^{13}**C NMR** spectrum of **4aa** (DMSO-d_6)

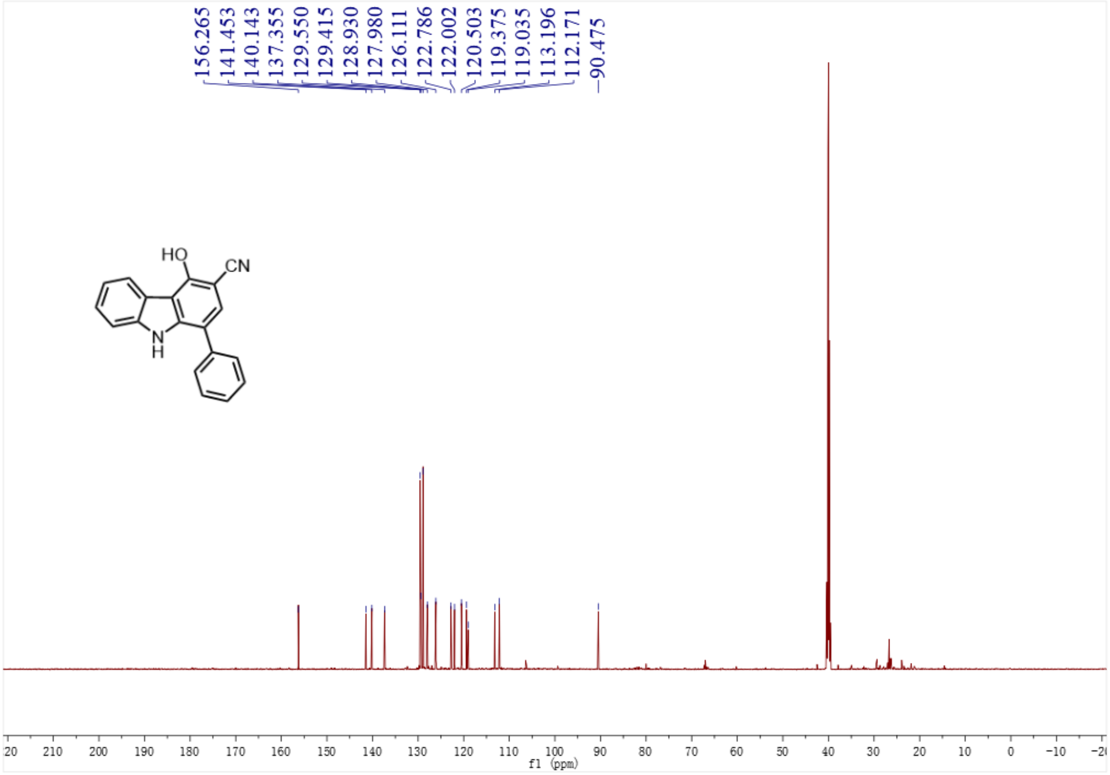

^{1}H NMR spectrum of **6c** ($CDCl_3$)

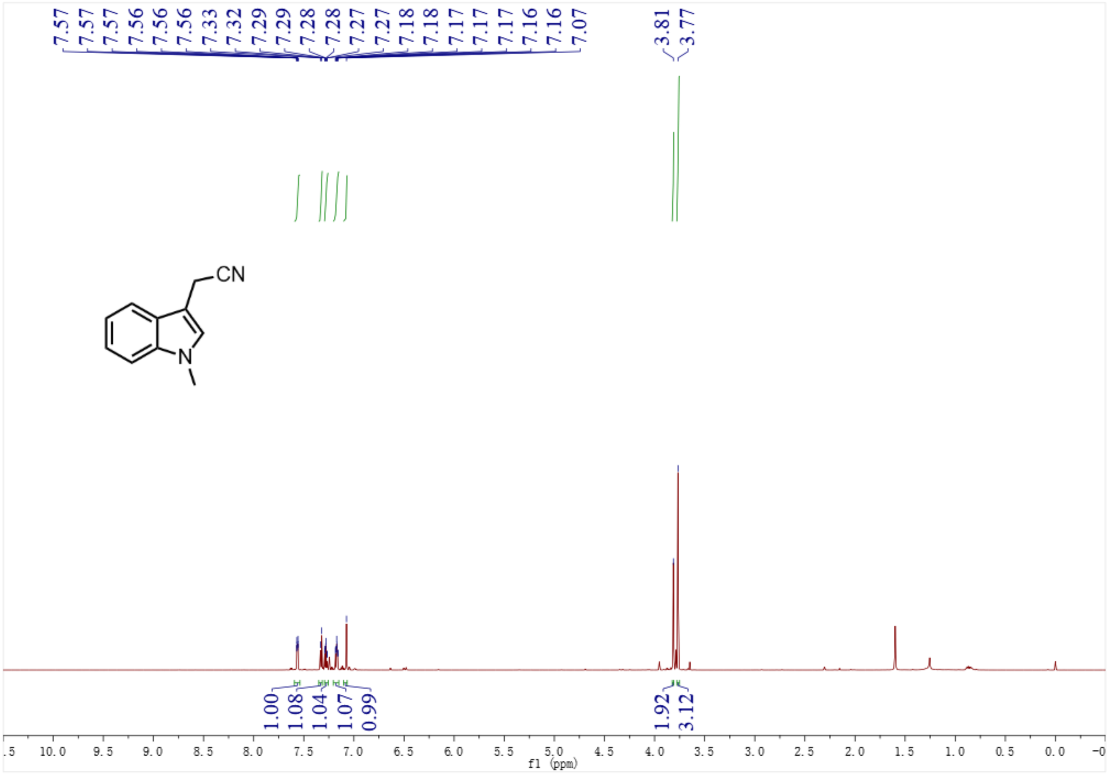

^{13}C NMR spectrum of **6c** ($CDCl_3$)

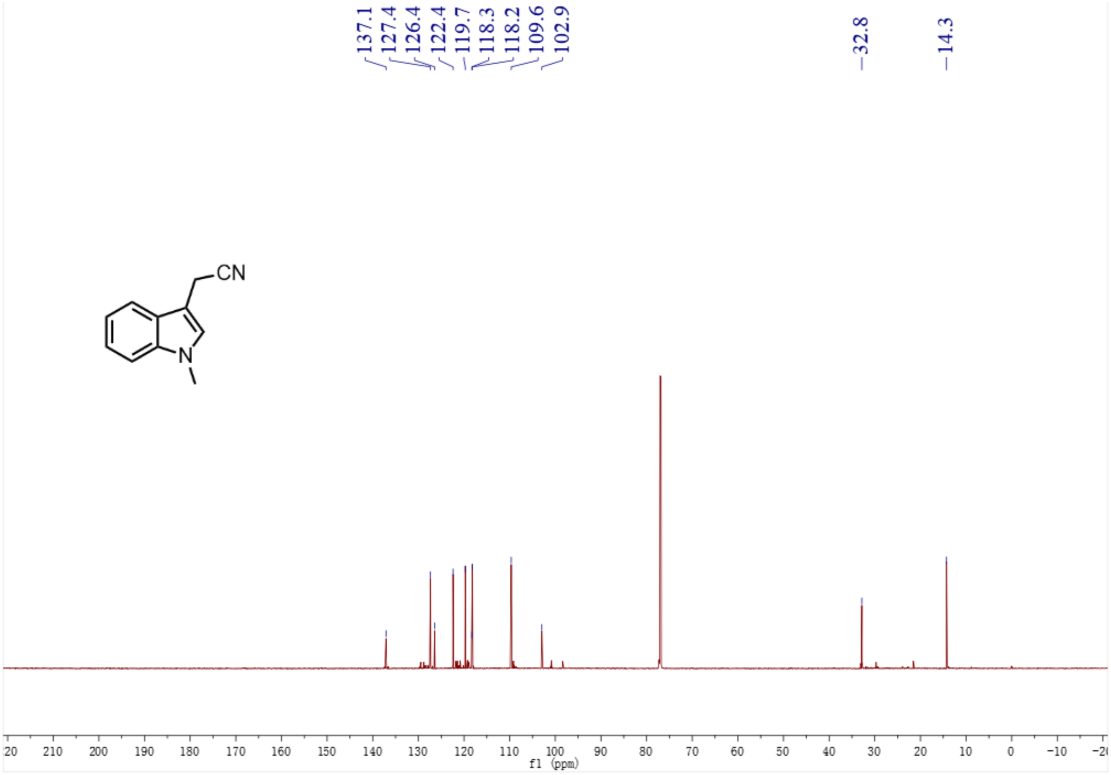

^{1}**H NMR** spectrum of **6d** ($CDCl_3$)

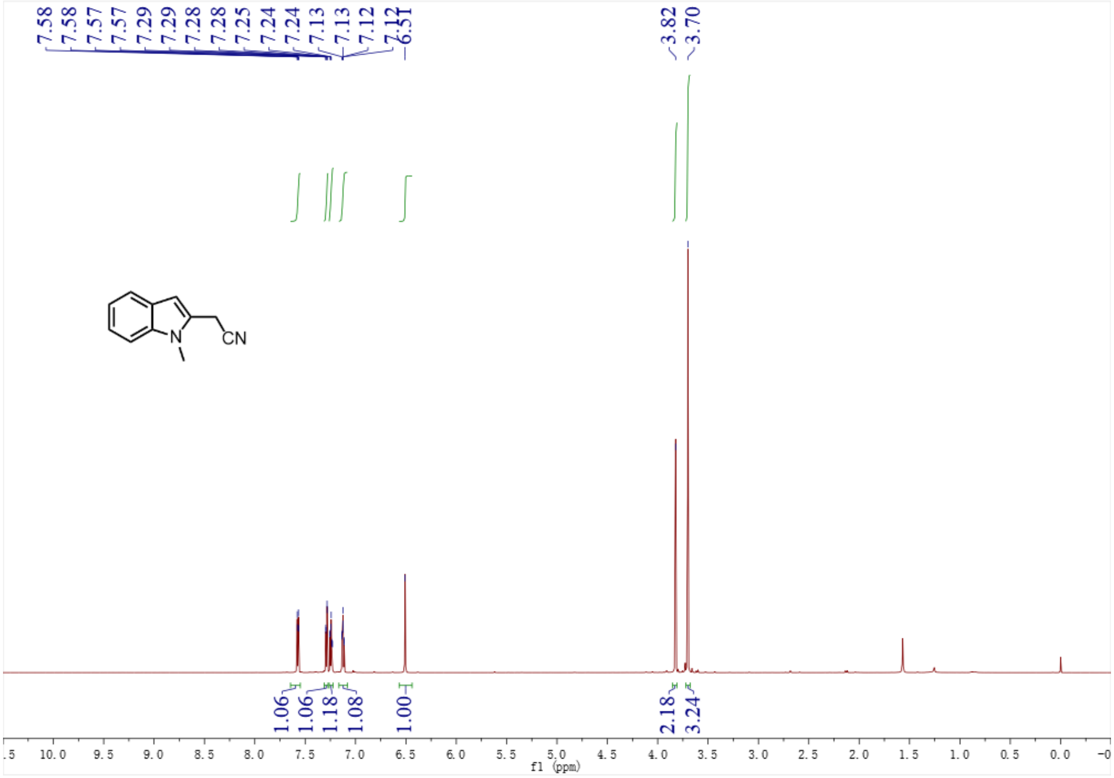

^{13}**C NMR** spectrum of **6d** ($CDCl_3$)

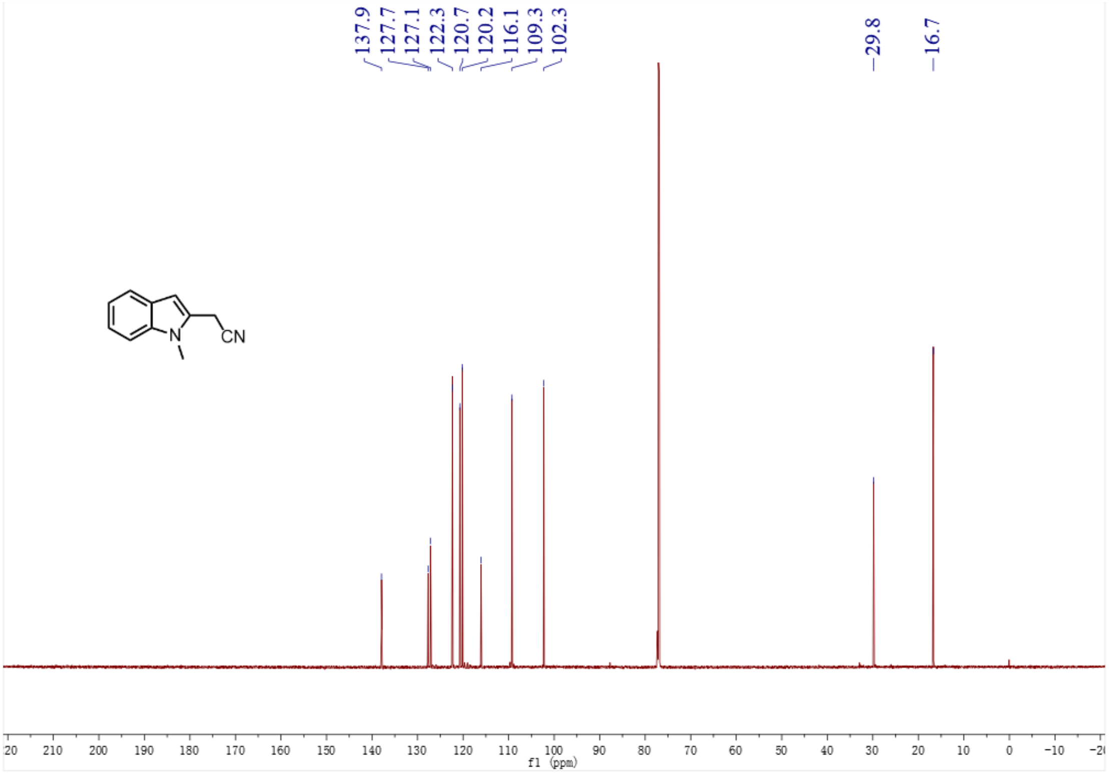

^{1}H **NMR** spectrum of **6e** ($CDCl_3$)

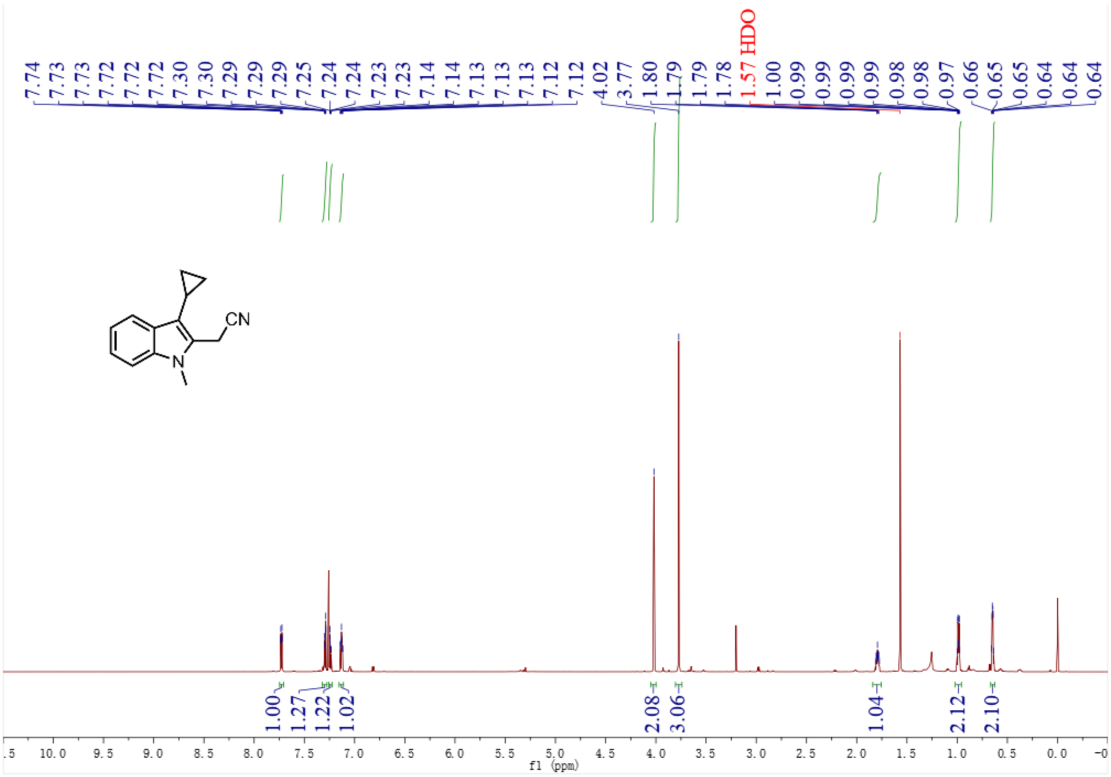

^{13}C **NMR** spectrum of **6e** ($CDCl_3$)

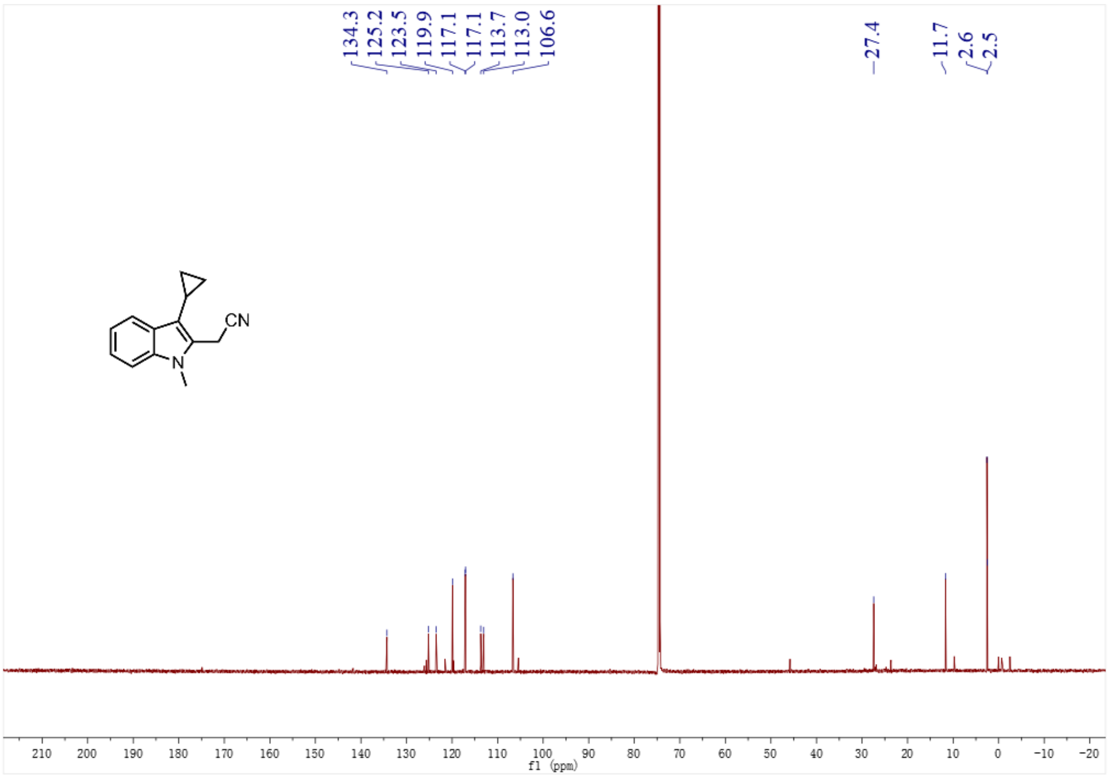

Supplement: SC-016-D5SC05810D-s001 [file SC-016-D5SC05810D-s001.pdf]
